# Prolonged wait time is associated with increased mortality for Chilean waiting list patients with non-prioritized conditions

**DOI:** 10.1186/s12889-019-6526-6

**Published:** 2019-02-26

**Authors:** Diego A. Martinez, Haoxiang Zhang, Magdalena Bastias, Felipe Feijoo, Jeremiah Hinson, Rodrigo Martinez, Jocelyn Dunstan, Scott Levin, Diana Prieto

**Affiliations:** 1 Johns Hopkins University School of Medicine, 1800 Orleans St, Baltimore, MD 21287 USA; 2 Johns Hopkins University Whiting School of Engineering, 3400 N Charles St, Baltimore, MD 21218 USA; 3 University of Chile School of Public Health, Av. Independencia 939, Independencia, Región Metropolitana Chile; 4 Pontifical Catholic University of Valparaíso School of Engineering, Brasil, 2950 Valparaíso, Región de Valparaíso Chile; 5 Johns Hopkins University Carey School of Business, 100 International Drive, Baltimore, MD 21202 USA

**Keywords:** Waiting lists, Health equity, Mortality, Delivery of health care, Engineering

## Abstract

**Background:**

Most data on mortality and prognostic factors of universal healthcare waiting lists come from North America, Australasia, and Europe, with little information from South America. We aimed to determine the relationship between medical center-specific waiting time and waiting list mortality in Chile.

**Method:**

Using data from all new patients listed in medical specialist waitlists for non-prioritized health problems from 2008 to 2015 in three geographically distant regions of Chile, we constructed hierarchical multivariate survival models to predict mortality risk at two years after registration for each medical center. Kendall rank correlation analysis was used to measure the association between medical center-specific mortality hazard ratio and waiting times.

**Result:**

There were 987,497 patients waiting for care at 77 medical centers, including 33,546 (3.40%) who died within two years after registration. Male gender (hazard ratio [HR] = 1.17, 95% confidence interval [CI] 1.1–1.24), older age (HR = 2.88, 95% CI 2.72–3.05), urban residence (HR = 1.19, 95% CI 1.09–1.31), tertiary care (HR = 2.2, 95% CI 2.14–2.26), oncology (HR = 3.57, 95% CI 3.4–3.76), and hematology (HR = 1.6, 95% CI 1.49–1.73) were associated with higher risk of mortality at each medical center with large region-to-region variations. There was a statistically significant association between waiting time variability and death (Z = 2.16, *P* = 0.0308).

**Conclusion:**

Patient wait time for non-prioritized health conditions was associated with increased mortality in Chilean hospitals.

**Electronic supplementary material:**

The online version of this article (10.1186/s12889-019-6526-6) contains supplementary material, which is available to authorized users.

## Background

Universal access to high-quality healthcare is a goal many countries strive for [1–4]. To optimize allocation and distribution of spending, countries have implemented large reforms that build capacity, prioritize resources, and set explicit waiting time targets for conditions defined through cost-benefit analysis [5]. Results of such health-system strengthening efforts and their effects on the health of people suffering non-prioritized health problems in South America are relevant for other low- and middle-income countries advancing towards universal healthcare [6, 7]. Chilean experience has generated evidence of particular relevance for countries in the region seeking to achieve universal healthcare (e.g., see evidence from Bolivia [8], Brazil [9, 10], Colombia [11], Cuba [12], and Mexico [13]).

Chile’s Health System is a two-tier system with 78% of the population under public insurance and the remaining covered by other mechanisms including private and military insurance [14]. The public system consists of the Ministry of Health (MINSAL) and its 33 Regional Health Services (RHS), which are administrative agencies overseeing the provision of healthcare at tertiary, secondary, and primary medical centers. Since 2005, the public system guarantees access to care with limited waiting time and out-of-pocket payment for a specific set of health problems under the Health Explicit Guarantees (GES) Act (previously named “Plan AUGE”) [15–17]. The prioritized health problems were selected on the basis of disease burden and social preference [18], and they consume the vast majority of installed capacity resulting in prolonged waiting times for the remaining “non-prioritized” health problems. There were over three million new specialty referrals for patients with non-prioritized conditions in 2016, out of which 43% remained in the waiting list [14]. As such, Chilean waiting lists have been intensely scrutinized and criticized in the press and by policy experts. Much of the criticism has focused on a perceived higher waiting list morbidity and mortality as well as on the wide variations in the time that patients must wait [19]. In 2017, in response to these concerns, the Congress of Chile commissioned medicine and public health experts to study the wait list, with a focus on patients who died while waiting or soon after [20].

The longest waiting lists in Chile are those for conditions not prioritized by the GES Act. Therefore, the present study of waiting list outcomes focuses on non-prioritized patients referred to a specialist for the first time. The objective of this study was to analyze how medical center-specific waiting time performance is related to medical center-specific mortality risk. We hypothesized that patients who wait longer to see a specialist were at an increased risk of death.

## Methods

**Fig. 1:**
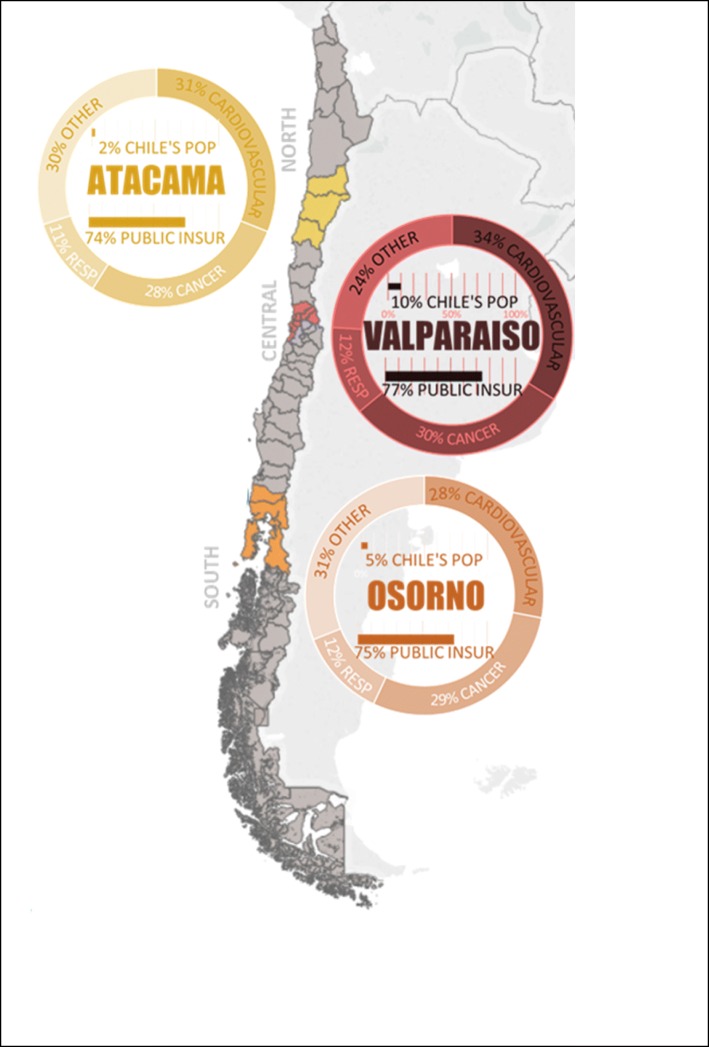
Map of Chile and the Location of the Regional Health Services under Study. Death causes data source: Death Causes – Health Statistics and Information Department 2014. Public health insurance data source: FONASA 2017. Population size data source: Population Projections 2018

Our study cohort consisted of all patients listed on a non-prioritized waiting list between January 1, 2008, and December 31, 2015. Data were collected from de-identified and publically available waiting list databases of three geographically distant RHS (Atacama, Valparaiso-San Antonio, and Osorno). We chose this cohort of patients from a recent ten-year period during which waiting list policy was stable and relatively current (see Policy Timeline in the Additional file 1). The three RHS selected for this study were located in three distinct natural regions: North, Central, and South Chile (see Fig. 1). Sufficient follow-up time was included to accurately determine the risk of death within 2 years of registration. During our study period, entry onto the waiting list was at the discretion of the medical centers, and hence we chose to assess medical center-level analyses of waiting time and mortality risk.

**Fig. 2:**
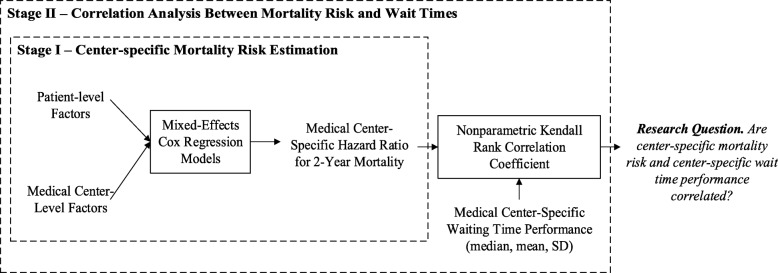
Study Design. Abbreviations: SD, standard deviation

In initial explorations (see the Extension and Robustness of Main Results in the Additional file 1), we found patients who died had consistently shorter wait times, which can be explained by subconscious triage effect. Hence, to isolate the effect of waiting on outcomes, we measured the association of mortality and waiting at the medical center-level following the two-stage study design depicted in Fig. 2 (following the study designs presented in [21–25]). Stage I provides hazard ratio (HR) estimates for each medical center’s risk of death within 2 years from patient registration on the waiting list. Stage II measures the association between these HR and the central tendency and dispersion of waiting time at each medical center. This two-stage approach allows us profile the outcome performance of medical centers considering the patient characteristics of the population they serve.

In stage I, seven covariates were used to create our model including patient’s age at listing, sex, insurance coverage, area of residence, consulted medical specialty, and referring and accepting medical center type. Mixed-effects Cox regression models (also referred to as frailty Cox models, hierarchical, or random effects survival models) were constructed to estimate the HR for waiting list mortality at each medical center and the various covariates previously described [26, 27]. We selected a mixed-effects modeling approach to account for clustered measures, i.e., those patients waiting at medical centers of lower complexity (e.g., primary) are more similar than those waiting at medical centers of higher complexity (e.g., secondary, tertiary). These clustered observations were specified in the models as crossed random effects on the RHS and Accepting Medical Center covariates. The proportional hazards assumption was assessed graphically for the statistically significant covariates. Factors not satisfying this assumption were included as stratification factors in the model. To comply with the broad opinion of the medical community that the level of care must be taken into account when comparing medical centers’ waiting list outcomes [19]. Patients were stratified at the time of listing, and their HR for death within 2 years of registration was calculated.

In stage II, each medical center’s median wait time was first plotted against their HR. Our working hypothesis was that patients who wait longer to see a specialist were at an increased risk of death; hence, the main explanatory variable is waiting time and is defined as the median wait until the next available appointment at a public healthcare facility. We measured the ordinal association between mortality risk and waiting time using the nonparametric Kendall rank correlation coefficient [28]. The results were plotted as scatterplots to show variation and provide a complete picture of the data. We tested the robustness of our main findings to 1) the exclusion of low-mortality medical specialties, 2) the stratification by level of care provided at each medical center, and 3) the outcome definition of 2-year mortality versus 2·5- and 3-year mortality. Statistical analyses were performed in R version 3.5.1 with the freely available statistical packages *survival* version 2.41–3 [29], *coxme* version 2.2–10 [30], and *frailtypack* version 2.12.6 [31]. To facilitate study replicability, we have included all R scripts built for data cleaning and analysis (see Additional files 2 and 3).

## Results

During our study period, 987,497 patients were added to the non-prioritized waiting list. A total of 161 tertiary, secondary, and primary care medical centers added patients to the waiting list at the three geographically distant RHS under study. All patients were followed up until specialist consultation, surgery, death, or removal from the waiting list due to clinical or administrative reasons. As presented in Table 1, majority of patients were adults between 15 and 45 years old (30%), female (62%), publically insured (99%), urban residing (65%), and waiting to be seen at a tertiary care medical centers (91%). The median waiting time was 68 days (mean 190 days, Q25% 25 days, Q75% 204 days) and the overall 2-year mortality rate was 3‧4%. Specialties with the largest waiting lists were Dentistry (13%), Traumatology (11%), and Ophthalmology (12%) followed by Obstetrics and Gynecology (10%), Adult General Surgery (7%), and Otorhinolaryngology (7%).

**Table 1:** Hazard Ratio for Mortality Within Two Years of Listing According-Patient Characteristics

	Overall^b^ *N* = 987,497		Atacama^a^ *N* = 264,756		Valparaiso-San Antonio^a^ *N* = 457,928		Osorno^a^ *N* = 264,813	
	N (%)	HR (95% CI)	N (%)	HR (95% CI)	N (%)	HR (95% CI)	N (%)	HR (95% CI)
2-Year Mortality Rate	33,546 (3.40)		7334 (2.77)		18,408 (4.02)		7804 (2.95)	
Age (15–45 comparator)								
0–3	68,028 (7)	0.76 (0.69–0.84)^c^	18,405 (7)	0.77 (0.6–0.99)^e^	34,224 (7)	0.78 (0.69–0.87)^c^	15,399 (6)	0.31 (0.21–0.45)^c^
4–7	53,865 (5)	0.16 (0.12–0.2)^c^	14,893 (6)	0.21 (0.13–0.34)^c^	24,158 (5)	0.15 (0.11–0.21)^c^	14,814 (6)	0.09 (0.04–0.19)^c^
8–11	43,053 (4)	0.1 (0.07–0.14)^c^	12,793 (5)	0.08 (0.04–0.19)^c^	19,580 (4)	0.11 (0.07–0.16)^c^	10,680 (4)	0.05 (0.02–0.16)^c^
12–14	35,723 (4)	0.18 (0.14–0.24)^c^	10,178 (4)	0.22 (0.12–0.39)^c^	16,895 (4)	0.16 (0.11–0.23)^c^	8650 (3)	0.17 (0.09–0.35)^c^
15–45	293,892 (30)		84,265 (32)		125,589 (27)		84,038 (32)	
46–55	146,814 (15)	2.88 (2.72–3.05)^c^	39,404 (15)	2.94 (2.59–3.34)^c^	66,267 (14)	2.79 (2.58–3.01)^c^	41,143 (16)	2.93 (2.6–3.31)^c^
56–65	139,099 (14)	5.47 (5.19–5.76)^c^	35,391 (13)	6.49 (5.8–7.27)^c^	67,223 (15)	4.88 (4.55–5.24)^c^	36,485 (14)	5.77 (5.17–6.43)^c^
66–75	122,319 (12)	9.11 (8.66–9.57)^c^	29,264 (11)	12.22 (10.96–13.61)^c^	61,731 (13)	7.58 (7.08–8.1)^c^	31,324 (12)	10.15 (9.14–11.26)^c^
76–85	70,835 (7)	16.11 (15.32–16.94)^c^	17,130 (6)	20.14 (18.06–22.46)^c^	35,495 (8)	13.63 (12.74–14.59)^c^	18,210 (7)	18.13 (16.34–20.12)^c^
85+	13,869 (1)	31.77 (30.01–33.63)^c^	3033 (1)	43.11 (38.1–48.79)^c^	6766 (1)	27.18 (25.15–29.37)^c^	4070 (2)	32.16 (28.63–36.12)^c^
Sex (Female comparator)								
Female	613,499 (62)		166,045 (63)		282,601 (62)		164,853 (62)	
Male	373,998 (38)	1.65 (1.61–1.69)^c^	98,711 (37)	1.75 (1.67–1.84)^c^	175,327 (38)	1.66 (1.61–1.71)^c^	99,960 (38)	1.51 (1.44–1.58)^c^
Residence (Other comparator)								
Rural	20,271 (2)		16,267 (6)		1854 (< 1)		2150 (1)	
Other	320,563 (32)	1.72 (1.56–1.89)^c^	36,555 (14)	1.98 (1.77–2.23)^c^	36,258 (8)	1.5 (1.21–1.84)^c^	247,750 (94)	1.44 (0.91–2.26)
Urban	646,663 (65)	1.19 (1.09–1.31)^c^	211,934 (80)	1.13 (1.02–1.26)^e^	419,816 (92)	1.2 (0.98–1.47)	14,913 (6)	0.85 (0.52–1.39)
Health Service (Atacama comparator)								
Atacama	264,756 (27)	Std Dev = 0.02		Not applicable		Not Applicable		Not Applicable
Osorno	457,928 (46)							
Valparaiso-San Antonio	264,813 (27)							
Health Insurance (Public comparator)								
Public	979,666 (99)		263,420 (99)		453,985 (99)		262,261 (99)	
Other (Private, Military)	7831 (1)	0.85 (0.73–0.99)^e^	1336 (1)	0.75 (0.5–1.12)	3943 (1)	0.73 (0.58–0.92)^d^	2552 (1)	1.15 (0.91–1.47)
Specialty (Internal Medicine comparator)								
Internal Medicine	46,767 (5)		19,054 (7)		20,829 (5)		6884 (3)	
Adult Surgery	71,148 (7)	0.67 (0.64–0.7)^c^	16,667 (6)	0.91 (0.83–1)^e^	37,534 (8)	0.55 (0.52–0.58)^c^	16,947 (6)	0.74 (0.66–0.82)^c^
Anesthesiology	3978 (< 1)	0.38 (0.33–0.44)^c^	0 (< 1)	Not applicable	3365 (1)	0.34 (0.29–0.39)^c^	613 (< 1)	0.28 (0.17–0.47)^c^
Breast Surgery	1478 (< 1)	0.55 (0.39–0.77)^c^	0 (< 1)	Not applicable	1478 (< 1)	0.52 (0.37–0.73)^c^	0 (< 1)	Not applicable
Pulmonary	17,427 (2)	1.17 (1.1–1.24)^c^	3028 (1)	2.05 (1.8–2.32)^c^	9222 (2)	1.02 (0.94–1.1)	5177 (2)	0.96 (0.84–1.09)
Cardiology	31,480 (3)	0.66 (0.63–0.7)^c^	6473 (2)	0.87 (0.77–0.98)^e^	16,583 (4)	0.6 (0.56–0.64)^c^	8424 (3)	0.59 (0.53–0.67)^c^
Cardiovascular Surgery	15,197 (2)	0.63 (0.58–0.68)^c^	2453 (1)	1.05 (0.88–1.24)	5687 (1)	0.56 (0.51–0.62)^c^	7057 (3)	0.51 (0.44–0.58)^c^
Dentistry	128,505 (13)	0.33 (0.31–0.36)^c^	17,538 (7)	0.5 (0.43–0.57)^c^	74,765 (16)	0.37 (0.32–0.42)^c^	36,202 (14)	0.22 (0.19–0.25)^c^
Dermatology	28,987 (3)	0.42 (0.38–0.46)^c^	11,076 (4)	0.62 (0.54–0.72)^c^	6261 (1)	0.39 (0.34–0.46)^c^	11,650 (4)	0.3 (0.25–0.36)^c^
Endocrinology	17,854 (2)	0.43 (0.39–0.49)^c^	3836 (1)	0.48 (0.36–0.65)^c^	9662 (2)	0.42 (0.36–0.48)^c^	4356 (2)	0.36 (0.28–0.47)^c^
Gastroenterology	25,745 (3)	1.03 (0.98–1.09)	5057 (2)	1.3 (1.14–1.48)^c^	13,282 (3)	0.93 (0.86–1)^e^	7406 (3)	0.97 (0.86–1.1)
Genetics	3348 (< 1)	0.64 (0.46–0.89)^d^	2267 (1)	0.54 (0.33–0.89)^e^	1081 (< 1)	1.06 (0.69–1.64)	0 (< 1)	–
Hematology	5868 (1)	1.6 (1.49–1.73)^c^	0 (< 1)	Not applicable	2929 (1)	1.14 (1.02–1.27)^e^	2939 (1)	1.89 (1.68–2.13)^c^
Infectious Disease	3304 (< 1)	0.86 (0.7–1.05)	628 (< 1)	0.61 (0.34–1.1)	2676 (1)	0.8 (0.65–0.99)^e^	0 (< 1)	Not applicable
Maxillofacial Surgery	18,140 (2)	0.37 (0.31–0.43)^c^	3472 (1)	0.37 (0.25–0.55)^c^	4494 (1)	0.3 (0.22–0.41)^c^	10,174 (4)	0.39 (0.31–0.48)^c^
Neonatology	282 (< 1)	1.61 (0.84–3.12)	0 (< 1)	Not applicable	282 (< 1)	1.32 (0.68–2.56)	42 (< 1)	0.00 (0–9.99)
Nephrology	11,208 (1)	1.02 (0.96–1.1)	1975 (1)	1.01 (0.85–1.2)	6659 (1)	1 (0.91–1.08)	2574 (1)	0.89 (0.76–1.04)
Neurology	47,087 (5)	0.82 (0.78–0.86)^c^	12,899 (5)	1.13 (1.01–1.26)^e^	23,795 (5)	0.72 (0.66–0.77)^c^	10,393 (4)	0.75 (0.67–0.85)^c^
Neurosurgery	15,238 (2)	0.5 (0.46–0.55)^c^	2856 (1)	0.96 (0.78–1.18)	7667 (2)	0.43 (0.38–0.48)^c^	4715 (2)	0.43 (0.35–0.51)^c^
Nutrition	1856 (< 1)	2.16 (1.81–2.57)^c^	0 (< 1)	Not applicable	1702 (< 1)	1.82 (1.52–2.17)^c^	154 (< 1)	0 (0–4.334E+ 252)
Obstetrics & Gynecology	93,979 (10)	0.42 (0.39–0.45)^c^	24,228 (9)	0.47 (0.4–0.56)^c^	48,395 (11)	0.41 (0.38–0.45)^c^	21,356 (8)	0.35 (0.29–0.42)^c^
Oncology	6080 (1)	3.57 (3.4–3.76)^c^	1080 (< 1)	3.71 (3.24–4.26)^c^	3285 (1)	2.64 (2.47–2.83)^c^	1715 (1)	5.19 (4.68–5.75)^c^
Ophthalmology	113,848 (12)	0.34 (0.32–0.36)^c^	43,852 (17)	0.5 (0.46–0.55)^c^	30,438 (7)	0.32 (0.29–0.34)^c^	39,558 (15)	0.25 (0.22–0.28)^c^
Other	1063 (< 1)	1.04 (0.76–1.44)	428 (< 1)	2.2 (1.51–3.2)^c^	329 (< 1)	0.41 (0.2–0.82)^e^	306 (< 1)	1.3 (0.18–9.23)
Otorhinolaryngology	67,646 (7)	0.43 (0.4–0.45)^c^	19,378 (7)	0.59 (0.53–0.65)^c^	28,970 (6)	0.39 (0.36–0.43)^c^	19,298 (7)	0.34 (0.3–0.38)^c^
Pediatrics	18,177 (2)	0.41 (0.31–0.54)^c^	8863 (3)	0.66 (0.44–0.98)^e^	5519 (1)	0.28 (0.17–0.47)^c^	3795 (1)	0.85 (0.44–1.63)
Physical Med & Rehabilitation	10,429 (1)	0.59 (0.53–0.65)^c^	0 (< 1)	Not applicable	7015 (2)	0.53 (0.48–0.6)^c^	3414 (1)	0.42 (0.31–0.56)^c^
Plastic Surgery	1858 (< 1)	0.53 (0.43–0.67)^c^	426 (< 1)	0.97 (0.63–1.52)	1432 (< 1)	0.42 (0.33–0.55)^c^	0 (< 1)	Not applicable
Colorectal Surgery	3114 (< 1)	0.57 (0.47–0.69)^c^	0 (< 1)	Not applicable	328 (< 1)	0.44 (0.27–0.72)^d^	2786 (1)	0.54 (0.44–0.67)^c^
Psychiatry	13,135 (1)	0.74 (0.65–0.85)^c^	4068 (2)	1.24 (0.99–1.54).	6744 (1)	0.62 (0.51–0.74)^c^	2323 (1)	0.66 (0.45–0.97)^e^
Rheumatology	7341 (1)	0.4 (0.35–0.47)^c^	0 (< 1)	Not applicable	2898 (1)	0.46 (0.38–0.55)^c^	4443 (2)	0.3 (0.25–0.38)^c^
Sexual Transmitted Disease	1807 (< 1)	0.3 (0.17–0.53)^c^	420 (< 1)	0.37 (0.09–1.47)	1250 (< 1)	0.28 (0.15–0.53)^c^	137 (< 1)	0 (0–6.538E+ 282)
Traumatology	107,111 (11)	0.34 (0.32–0.36)^c^	33,661 (13)	0.46 (0.41–0.51)^c^	50,156 (11)	0.3 (0.28–0.32)^c^	23,294 (9)	0.32 (0.28–0.36)^c^
Urology	47,102 (5)	0.58 (0.55–0.61)^c^	19,073 (7)	0.76 (0.69–0.84)^c^	21,066 (5)	0.54 (0.5–0.58)^c^	6963 (3)	0.44 (0.39–0.51)^c^
Referring Medical Center (Primary comparator)								
Primary	463,119 (47)		10,180 (4)		283,745 (62)		169,194 (64)	
Secondary	3455 (< 1)	0.74 (0.52–1.04)	0 (< 1)	Not applicable	3455 (1)	0.74 (0.53–1.05)	0 (< 1)	Not applicable
Tertiary	685,483 (69)	2.2 (2.14–2.26)^c^	254,576 (96)	0.59 (0.47–0.73)^c^	170,728 (37)	2.39 (2.31–2.47)^c^	260,179 (98)	1.71 (1.63–1.8)^c^
Accepting Medical Center (Primary comparator)								
Primary	29,622 (3)	Std Dev = 0.79	9359 (4)		15,629 (3)		4634 (2)	
Secondary	59,672 (6)		0 (< 1)	Not applicable	59,672 (13)	2.62 (1.94–3.53)^c^	0 (< 1)	Not applicable
Tertiary	898,203 (91)		255,397 (96)	4.95 (3.42–7.16)^c^	382,627 (84)	3.83 (2.94–4.99)^c^	260,179 (98)	5.55 (2.26–13.6)^c^

Abbreviations: *N* number of patients, *HR* hazard ratio, *CI* confidence interval, *Std Dev* standard deviation, *Med* medicine

^a^ Results are from Cox proportional hazard models fit by maximum likelihood

^b^ Results are from mixed-effects Cox proportional hazard models with Regional Health Service and Accepting Medical Center included as a crossed random effect

^c^ Significant at the 0.1% level, ^d^ Significant at the 1% level, ^e^ Significant at the 5% level

Table 1 also shows that HR for death increased with age, with the exception of patients aged 0–3 years old who were at higher risk than other pediatric populations. Male patients were at higher risk of death as compared to female (HR = 1·65, 95% CI 1·61–1·69). Patients listed with residency in rural areas were at lower risk of death as compared to those living in urban areas (HR = 1·19, 95% CI 1·09–1·31). Patients referred from tertiary care centers were at higher risk of death compared to those referred from primary and secondary care centers (HR = 2·2, 95% CI 2·14–2·26).

Large region-to-region variations were found. High-risk specialty referrals among all regions were oncology (HR 3·57 95% CI 3·4–3·76) and hematology (HR = 1·6, 95% CI 1·49–1·73). In Atacama, however, additional high-risk specialties included pulmonary (HR = 2·05, 95% CI 1·8–2·32) and gastroenterology (HR = 1·3, 95% CI 1·14–1·48). The amount of variability of RHS and accepting medical center types were expressed by the model as standard deviations in the HR. The standard deviation of the HR associated with RHS was relatively small (Std Dev 0·02), indicating RHS that are one standard deviations away from the mean (HR 1·0) would have 2% lower or higher mortality risk. In contrast, the standard deviation of the HR associated with accepting medical center was large (Std Dev 0·79), indicating that medical centers that are one standard deviations way from the mean (HR 1·0) would have 220% lower or higher risk of death. Similar results were obtained upon examination of 2·5- and 3-year mortality (see Robustness to outcome definition in the Additional file 1).

**Fig. 3:**
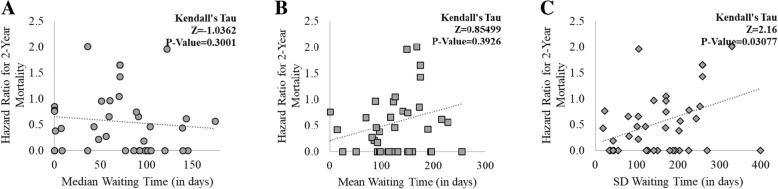
Association between Medical Center-Specific Hazard Ratio (HR) for Death and Medical Center-Specific Waiting Time Performance. Panel **a**, median waiting time against HR; Panel **b**, mean waiting time against HR; Panel **c**, standard deviation of waiting time against HR. Kendall rank correlation coefficient measured no statistically significant association between the medical centers’ median and mean waiting times and the medical centers’ HR for death (Panel **a** and **b**). The same examination found a statistically significant positive correlation between the medical centers’ standard deviation of waiting time and the medical centers’ HR for death (Panel **c**). Medical centers with < 30 patients in their waiting lists during the study timeframe were excluded from the analyses. Abbreviations: SD, standard deviation

As shown in Fig. 3, medical centers’ waiting time performance was plotted against the HR for death on the waiting list for those same medical center’s patients. Overall, we found a statistically significant association between the high waiting time variability at each medical center and their associated HR for death (Z = 2·16, *P*-Value = 0·0308). We found no statistically significant association between the median (and mean) waiting time at each medical center and their HR for death (mean waiting time and HR, Z = − 1·0362, P-Value = 0·3001; median waiting time and HR, Z = 0·8550, P-Value = 0·3926). The result that waiting time variability is associated with increased HR for death remain when stratifying by the level of care provided at each medical center (see Correlation Analysis by Level of Care in the Additional file 1). However, when concentrating the analyses on a subset of high-mortality medical specialties (mortality rate > 50th percentile), we found a statistically significant association between prolonged waiting and increased HR for death (mean waiting time and HR, Z = 2·3690, P-Value = 0·0178; waiting time SD and HR, Z = 2·1600, P-Value = 0·02591) (see Correlation Analysis for High-Risk Medical Specialties in the Additional file 1). In our patient cohort, these high-mortality specialties included cardiology, cardiovascular surgery, gastroenterology, nephrology, neurology, and urology. The few outlier points seen in Fig. 3 were caused by a few medical centers listing < 30 patients during our study period. These patients were upgraded to higher acuity medical centers, making their waiting times extremely long. When these outliers were excluded from the analysis, we found no significant change in the results (data not shown).

## Discussion

Whether low- and middle-income countries can meet universal healthcare needs fairly is a prevalent policy question [1]. In a large and representative sample of the Chilean waiting list population, our analysis distinctly indicates several individual and geographical factors associated with increased risk of death. By far, the age and sex of the patient as well as the type of medical specialty for which the patient was listed were the most important factors overall associated with increased mortality risk. Additionally, we found a strong positive correlation between medical centers’ waiting time performance and mortality while controlling for patient, medical center, and geographical factors. This suggests that, if the sole goal is to reduce mortality risk in waiting lists not prioritized by the GES Act, how long patients wait and the variability of this waiting time at each medical center should influence the waiting list management system. Furthermore, that both our study and that of the MINSAL found waiting list mortality to be highly correlated with medical specialty indicates that these criteria have higher validity in predicting waiting list mortality (and therefore urgency for prioritization) for this cohort [32].

There are several possible explanations for the strong association of medical specialty with subsequent waiting list outcome. Cancer is the leading cause of death in Chilean waiting lists [32]. Recent data show a disproportionate prevalence and mortality of lung and gallbladder cancer, potentially linked to both genetic and socioeconomic factors [32–36]. While the implementation of the GES Act, previously known as the AUGE Act, has been associated with improvements in breast cancer management [37], our data show that further resources should be directed towards addressing persistent inequalities for other highly prevalent cancers (see Survival Analysis on Oncology in the Additional file 1). Recent proposals include strengthening the prevention and treatment of cancer by creating specialized medical centers across the nation, initiating prevention programs to decrease obesity and smoking for specific age and sex groups, and increasing the cancer and palliative care workforce [34, 38, 39]. Further research is needed to understand the epidemiology of non-prioritized cancer waitlists, in addition to investigate and anticipate workforce requirements for cancer and palliative care specialists.

Although there is evidence of improvements in access, quality, and costs due to the GES Act, there are persistent inequalities [40–51]. Our data show higher mortality risk for men, which might be due to several reasons: Chilean male patients have a higher prevalence of high-mortality conditions compared to women [18], they consult less [52], and they have not been targeted by the large and recent governmental health programs. The exact cause of increased risk for men is unknown to us and deserves further investigation. Our data also show adults older than 45 years are at higher risk of death compared to children, which again can be related to under-consultation, under-prioritization, or expected as it mirrors the natural age-specific mortality [53]. These findings suggest that a policy designed to protect a particularly vulnerable population [15–17], women and children, may have had unanticipated and untoward effects on a group traditionally thought of as low-risk: male adults. Whether or not Chile can meet the health needs of male adults continues to be a prevailing challenge that requires immediate action.

Another significant finding was that prolonged waiting time on the list is strongly associated with an increased risk of death. Furthermore, patients listed for high-mortality specialties (cardiology, cardiovascular surgery, gastroenterology, nephrology, neurology, and urology) are at even higher risk when exposed to prolonged waiting. Therefore, our data directly support even more priority being given to these more risky patients to reduce their waiting time than was prevailing during our study timeframe. Moreover, waiting time by itself is an outcome of interest related to patient experience. High-income countries, such as Finland, have used penalty mechanisms for medical centers with prolonged waiting times [54]. Multinational comparisons have shown England’s investments in infrastructure, workforce, and health problem prioritization strategies have been successful in reducing waiting lists [5]. All these strategies, after careful adaptation to Chile’s context, may offer sustained and long-term solutions to the Chilean waiting list challenges.

We found significant variation in the risk of death on the waiting list among medical centers and geographical regions. Again, this variation may be the source of substantial controversy and might be used to advocate changes to the waiting list system to equalize inequalities. That we observed different waiting list mortality risks suggests geographic factors influence waiting list mortality. However, many of these factors are not necessarily affected by, or likely to be corrected with, changes in the waiting list system. Previous data indicate wide variations in waiting list system efficiency across the nation and local variations in specialist availability [55–57]. Furthermore, tertiary care centers operating alone in a single region may have very different listing criteria compared with those operating in close proximity to other tertiary centers. All these factors are subject to change at any time and probably contribute to the variations in mortality risk we have described. In addition, differences in physician behavior over time make it difficult to quantify the results of diagnosis and referrals and predict results in the future. This might make generalization of our findings to the current waiting list system difficult because of changes in the waiting list sharing agreements between care centers. However, it is important to note that waiting list mortality risk variations among the three regions under study cannot be ascribed to greater proportions of more medically complex cases being cared for by the medical centers because we have controlled for the level of care provided by each center. Furthermore, whatever the shortcomings of the level of care definition prevailing during our study period, it is clear from our analysis that, in our patient cohort of non-prioritized health problems, the patient’s age and sex, as well as the consulted medical specialty, have as much association with death as does the medical center waiting time performance.

Our analysis right censored patients still on the list at the close of the study. The fate of these patients could potentially skew our results. Nonetheless, the majority of medical centers (81%) had median waiting times less than the 2-year follow up period used in our study and sensitivity analyses revealed no change in our findings due to longer follow up periods. For younger patients, it is possible that longer follow-up period would lead to fewer censored patients, but it is unlikely that this would cause the relationship between mortality and waiting time to change.

This study has limitations. First, given that systematic ascertainment as to why a patient is removed from a waiting list is still challenging in Chile and elsewhere, it is likely that the presented mortality risks are underestimated. Other common issues such as not being able to contact the patient and subsequent attrition are familiar to most longitudinal studies [58]. Second, the Chilean waiting list electronic reporting system has deficiencies that might potentially bias our results. In systematic data cleaning (see Additional file 2), we excluded 4.07% of the records due to administrative reasons including “listed patient correspond to a prioritized health problem”, “digitation error”, “duplicated request”, and “missing patient contact information”. This is an inherent limitation of the current waiting list system and has been acknowledged in recent studies and reports from the MINSAL [19]. Third, as in any observational study, we cannot exclude the risk of confounding by other factors. Avenues for future exploration include examining the mediating role of comorbidities, demographics, clinical care, and other risk factors that could influence the association between waiting time and mortality. Finally, this study only analyzed events before or up to the visit to the specialist and it did not take into account the results after the visit. There is abundant evidence that organ transplantation, for example, of more medically urgent patients may result in poorer patient outcomes. Thus, waiting list reduction must balance between the need to equalize short-term and long-term survival. In addition to our finding that prolonged waiting time is associated with higher risk of death, we want to emphasize that prolonged wait to see a specialist is not a benign inconvenience—especially not when they are as long and systematic as those found in this study. Many patients face physical pain, mental distress, and loss of economic productivity while waiting to see their doctor.

## Conclusion

In summary, this study provides evidence of a strong association between prolonged waiting and increased risk of death among patients not protected by the GES Act. We believe that our data demonstrate the inability of the current waiting list system to identify patients who can safely wait, and thus underscore the need for rigorous wait time monitoring and for continued implementation of programs to prioritize access and to build hospital capacity. Chile is actively monitoring and working towards reducing delays, which has created valuable evidence to other low- and middle-income countries seeking to accomplish universal access to high-quality healthcare.

## Additional files

Additional file 1: Extension and Robustness of Main Results. (PDF 1480 kb)

Additional file 2: Data Cleaning Procedures. (PDF 116 kb)

Additional file 3: Data Analysis Procedures. (PDF 163 kb)

## References

[1] World Health Organization. The world health report: health systems financing: the path to universal coverage: executive summary (No. WHO/IER/WHR/10.1). Geneva: World Health Organization; 2010. 10.2471/BLT.10.078741 PMC2878164 20539847

[2] Reddy KS, Patel V, Jha P, Paul VK, Kumar AKS, Dandona L (2011). Towards achievement of universal health care in India by 2020: a call to action. Lancet (London, England).

[3] Backman G, Hunt P, Khosla R, Jaramillo-Strouss C, Fikre BM, Rumble C (2008). Health systems and the right to health: an assessment of 194 countries. Lancet (London, England).

[4] Birn A-E, Nervi L (2015). Political roots of the struggle for health justice in Latin America. Lancet (London, England).

[5] Willcox S, Seddon M, Dunn S, Edwards RT, Pearse J, Tu JV. Measuring and reducing waiting times: a cross-national comparison of strategies. Health Aff (Millwood). 26:1078–87. 10.1377/hlthaff.26.4.1078 17630450

[6] Atun R, de Andrade LOM, Almeida G, Cotlear D, Dmytraczenko T, Frenz P (2015). Health-system reform and universal health coverage in Latin America. Lancet (London, England).

[7] Titelman D, Cetrángolo O, Acosta OL (2015). Universal health coverage in Latin American countries: how to improve solidarity-based schemes. Lancet (London, England).

[8] Medici A (2015). Challenges and perspectives for tertiary level hospitals in Bolivia: the case of Santa Cruz de La Sierra Department. World Hosp Health Serv.

[9] Cesena FHY, Favarato D, César LAM, de Oliveira SA, da Luz PL (2004). Cardiac complications during waiting for elective coronary artery bypass graft surgery: incidence, temporal distribution and predictive factors. Eur J Cardiothorac Surg.

[10] Haddad N, Bittar OJNV, Pereira AAM, da Silva MB, Amato VL, Farsky PS (2002). Consequences of the prolonged waiting time for patient candidates for heart surgery. Arq Bras Cardiol.

[11] de Vries E, Buitrago G, Quitian H, Wiesner C, Castillo JS (2018). Access to cancer care in Colombia, a middle-income country with universal health coverage. J Cancer Policy.

[12] De Vos P, Orduñez-García P, Santos-Peña M, Van der Stuyft P (2010). Public hospital management in times of crisis: lessons learned from Cienfuegos, Cuba (1996-2008). Health Policy.

[13] Contreras-Loya D, Gómez-Dantés O, Puentes E, Garrido-Latorre F, Castro-Tinoco M, Fajardo-Dolci G. Waiting times for surgical and diagnostic procedures in public hospitals in Mexico. Salud Publica Mex. 57:29–37 http://www.ncbi.nlm.nih.gov/pubmed/25629277. 10.21149/spm.v57i1.7400 25629277

[14] FONASA. Boletin Estadistico 2016-2017. 2018. https://www.fonasa.cl/sites/fonasa/institucional/archivos.

[15] Letelier LM, Bedregal P (2006). Health reform in Chile. Lancet..

[16] MINSAL. Garantias Explicitas en Salud (AUGE o GES). 2018. http://www.supersalud.gob.cl/difusion/665/w3-propertyvalue-1962.html#. Accessed 14 Jun 2018.

[17] Bastías G, Pantoja T, Leisewitz T, Zárate V (2008). Health care reform in Chile. CMAJ..

[18] Vargas V, Poblete S. Health prioritization: the case of Chile. Health Aff (Millwood). 27:782–92. 10.1377/hlthaff.27.3.782 18474972

[19] MINSAL. Propuestas Ministerio de Salud por Informe de Comision Medica Asesora de Liastas de Espera. 2018. http://www.minsal.cl/comision-asesora-por-listas-de-espera/.

[20] MINSAL. Dispone Creación de Comisión Médica Asesora para Analizar la Situación de Personas que Fallecen Habiendo Estado en una Lista de Espera o con una Garantía de Oportunidad GES Retrasada. 2017. https://www.leychile.cl/Navegar?idNorma=1103060.

[21] Freeman RB, Edwards EB (2000). Liver transplant waiting time does not correlate with waiting list mortality: implications for liver allocation policy. Liver Transpl.

[22] Porell F, Caro FG, Silva A, Monane M (1998). A longitudinal analysis of nursing home outcomes. Health Serv Res.

[23] Mukamel DB, Spector WD (2000). Nursing home costs and risk-adjusted outcome measures of quality. Med Care.

[24] Porell F, Caro FG (1998). Facility-level outcome performance measures for nursing homes. Gerontologist..

[25] Burgess JF, Shwartz M, Stolzmann K, Sullivan JL (2018). The relationship between costs and quality in veterans health administration community living centers: an analysis using longitudinal data. Health Serv Res.

[26] Begg M, Parides M (2003). Separation of individual-level and cluster-level covariate effects in regression analysis of correlated data. Stat Med.

[27] Enders C, Tofighi D (2007). Centering predictor variables in cross-sectional multilevel models: a new look at an old issue. Psychol Methods.

[28] Kendall M (1938). A new measure of rank correlation. Biometrika..

[29] Therneau T. coxme: mixed effects Cox models. R package version 2.2-3. Vienna: R Foundation for Statistical Computing; 2012.

[30] Terry M T. Package “coxme.” 2012.

[31] Rondeau V, Gonzalez J (2005). frailtypack: A computer program for the analysis of correlated failure time data using penalized likelihood estimation. Comput Methods Programs Biomed.

[32] MINSAL. Estado de Situacion Personas Fallecidas en Listas de Espera No-GES y Garantias Retrasadas GES. 2017. http://www.minsal.cl/comision-asesora-por-listas-de-espera/.

[33] Develpment CM of S. Sintesis de Resultados en Salud CASEN. 2015.

[34] de la Jimenez Jara J, Bastias G, Ferreccio C, Moscoso C, Sagues S, Cid C (2015). A snapshot of cancer in Chile: analytical frameworks for developing a cancer policy. Biol Res.

[35] Izarzugaza MI, Fernández L, Forman D, Sierra MS (2016). Burden of gallbladder cancer in central and South America. Cancer Epidemiol.

[36] Andia ME, Hsing AW, Andreotti G, Ferreccio C (2008). Geographic variation of gallbladder cancer mortality and risk factors in Chile: a population-based ecologic study. Int J Cancer.

[37] Del Castillo SC, Cabrera CME, Derio PL, Gaete VF, Cavada CG (2017). Impact of the Chilean Explicit Guaranties Health System (GES) on breast cancer treatment. Rev Med Chil.

[38] Wild CP, Bucher JR, de Jong BWD, Dillner J, von Gertten C, Groopman JD (2015). Translational cancer research: balancing prevention and treatment to combat cancer globally. J Natl Cancer Inst.

[39] Juan Carlos Roa S, Catterina Ferreccio R, Juan Francisco Miquel P (2011). Cáncer de la vesícula biliar: estudios necesarios para el diseño de estrategias de prevención y diagnóstico precoz. Rev Médica Clínica Las Condes.

[40] Bitrán R, Escobar L, Gassibe P (2010). After Chile’s health reform: increase in coverage and access, decline in hospitalization and death rates. Health Aff (Millwood).

[41] Nazzal NC, Campos TP, Corbalán HR, Lanas ZF, Bartolucci JJ, Sanhueza CP (2008). The impact of Chilean health reform in the management and mortality of ST elevation myocardial infarction (STEMI) in Chilean hospitals. Rev Med Chil.

[42] Wang Y, Álvarez G, Salinas R, Ramírez G, Catalán M, Díaz C (2011). Compliance with Chilean diagnostic guidelines among patients with ischemic stroke admitted to a public hospital. Rev Med Chil.

[43] Paraje G, Vásquez F (2012). Health equity in an unequal country: the use of medical services in Chile. Int J Equity Health.

[44] Concha DF, Pastén VN, Espinosa FV, López AF. Impacto de la implementación del Plan AUGE en la detección antenatal de cardiopatías congénitas. Rev Chil Obstet Ginecol. 2008;73.

[45] Frenz P, Delgado I, Kaufman JS, Harper S (2014). Achieving effective universal health coverage with equity: evidence from Chile. Health Policy Plan.

[46] González F (2003). Implementation of AUGE plan for chronic renal failure patients. The implementation of universal access and explicit guaranties plan (AUGE), generates longer waiting lists among patients with chronic renal failure. Rev Med Chil.

[47] VEGA J, HOLLSTEIN RD, DELGADO I, PEREZ JC, CARRASCO S, MARSHALL G, et al. Chile: socioeconomic differentials and mortality in a middle-income nation. In: Challenging Inequities in Health. Oxford University Press; 2001. p. 122–137.

[48] Burrows J (2008). Inequalities and healthcare reform in Chile: equity of what?. J Med Ethics.

[49] Román O, Muñoz F (2008). A critical appraisal to “AUGE” health plan in Chile. Rev Med Chil.

[50] Nazzal C, Lanas F, Garmendia ML, Bugueño C, Mercadal E, Garcés E (2013). Universal health coverage and accomplishment of secondary prevention goals among patients with acute myocardial infarction. Rev Med Chil.

[51] Sánchez H, Albala C, Dangour AD, Uauy R (2009). Compliance with guidelines for the management of community acquired pneumonia at primary health care centers. Rev Med Chil.

[52] Vega J, Bedregal P, Jadue L, Delgado I (2003). Gender inequity in the access to health care in Chile. Rev Med Chil.

[53] GBD 2016 Mortality Collaborators (2017). Global, regional, and national under-5 mortality, adult mortality, age-specific mortality, and life expectancy, 1970–2016: a systematic analysis for the Global Burden of Disease Study 2016. Lancet (London, England).

[54] Siciliani L, Borowitz M, Moran V. Waiting Time Policies in the Health Sector: OECD Publishing; 2013.

[55] Guillou M, Carabantes CJ, Bustos FV (2011). Availability of physicians and specialists in Chile. Rev Med Chil.

[56] Román AO (2010). Evolution of the availability of physicians in Chile. Rev Med Chil.

[57] Román O, Acuña M, Señoret M (2006). Availability of physicians in Chile at the year 2004. Rev Med Chil.

[58] Banks J, Muriel A, Smith JP. Attrition and health in ageing studies: evidence from ELSA and HRS. Longit Life Course Stud. 2011;2. 10.14301/llcs.v2i2.115 PMC3872999 24376472

