# Supplementary material for: Prolonged wait time is associated with increased mortality for Chilean waiting list patients with non-prioritized conditions
Source: BMC Public Health. 2019 Feb 26;19:233. doi: 10.1186/s12889-019-6526-6 (PMC6390314; doi:10.1186/s12889-019-6526-6)
Supplement: Supplementary file 1 — Extension and Robustness of Main Results. (PDF 1480 kb) [file 12889_2019_6526_MOESM1_ESM.pdf]

Prolonged Wait Time is Associated with Increased Mortality for Chilean Waiting List Patients with Non-Prioritized Conditions

Diego A. Martinez, PhD,[1] Haoxiang Zhang, MSc,[1] Magdalena Bastias, PhD,[2] Felipe Feijoo,[3] PhD, Jeremiah Hinson, MD, PhD,[1] Rodrigo Martinez, MD,[2] Jocelyn Dunstan,[2] PhD, Scott Levin, PhD,[1] Diana Prieto, PhD[1]

[1]Johns Hopkins University
[2]University of Chile
[3]Pontifical Catholic University of Valparaíso

Additional File 1: Extension and Robustness of Main Results

Contents

Robustness of Main Results **2**
Survival Analysis on Oncology 2
Survival Analysis on 2-, 2.5-, and 3-Year Mortality 8
Correlation Analysis by Level of Care 10
Correlation Analysis for High-Risk Medical Specialties 11
Extensions to Main Analysis **12**
Methodological Justification of the Selected Study Design 12
Extra Data Visualizations **13**
Age- and Sex-Adjusted Mortality Rate 13
Specialty- and Sex-Adjusted Mortality Rates 16
Death-Adjusted Waiting Time Distribution 17
Policy Timeline **20**
Health Problems Prioritized by the GES Act 20
Data Collection and Analysis **21**

ROBUSTNESS OF MAIN RESULTS

Survival Analysis on Oncology

It is possible that our effects are driven by a set of few high-mortality medical specialties. We examine this possibility by restricting our sample to those registrants waiting for a high-mortality medical specialty: Oncology. Table S1 shows all variables included into the final model are statistically significant. Table S2 shows the HR associated with 2-year death since registration. Kaplan Meier estimator and Cox proportional model are depicted in Figure S1 and S2. While the confidence intervals are wider throughout due to the smaller sample size, the main patterns remain.

Table S1. ANOVA Analysis for Mortality Within 2 Years of Listing According to Covariates for Oncology specialty.

	loglik	*Chisq*	*df*	*Pr(>|Chi|)*	
NULL	-18771				
Age	-18619	304.7032	5	< 2.2e-16	***
Sex	-18592	54.6891	1	1.412E-13	***
Residence	-18532	118.9046	2	< 2.2e-16	***
Health Service	-18487	90.0868	2	< 2.2e-16	***
Referring Medical Center	-18530	5.1341	1	0.023460	*
Age : Sex	-18482	9.9105	5	7.78E-02	.
Age : Residence	-18472	19.7244	10	3.20E-02	*
Age : Health Service	-18451	43.3013	10	4.39E-06	***
Sex : Health Service	-18444	12.7144	6	4.78E-02	*
Age : Referring Medical Center	-18438	12.1335	2	2.32E-03	**

***Significant at the 0.1% level, ** Significant at the 1% level, *Significant at the 5% level.

Table S2. Hazard Risk for Mortality Within 2 Years of Listing According to Covariates: Oncology Patient Cohort.

	**N**	**HR (95% CI)**
**Age (reference level 85+)**		
15-45	827	0.017 (0.002-0.143)***
46-55	942	0.017 (0.002-0.122)***
56-65	1286	0.049 (0.009-0.273)***
66-75	1476	0.145 (0.029-0.732)*
76-85	863	0.256 (0.047-1.383)
**Sex (reference level female)**		
Male	2335	1.334 (0.926-1.920)
**Residence (reference level urban)**		
other	1818	0.387 (0.114-1.313)
rural	80	1.691 (0.373-7.664)
**Health Service (reference level Atacama)**		
Atacama	1003	0.116 (0.033-0.411)***
Vaparaiso-SanAntonio	2975	0.214 (0.060-0.767)*
**Referring Medical Center (reference level primary)**		
Tertiary	5459	0.509 (0.270-0.959)*
**Age : Sex**		
15-45:Male	288	0.653 (0.409-1.043).
46-55:Male	310	0.990 (0.644-1.522)
56-65:Male	506	1.019 (0.683-1.520)
66-75:Male	741	1.022 (0.690-1.514)
76-85:Male	420	0.740 (0.495-1.106)
**Age : Residency**		
15-45:other	240	3.885 (0.789-19.118).
46-55:other	267	3.863 (1.018-14.662)*
56-65:other	387	3.455 (0.928-12.858).
66-75:other	480	2.897 (0.795-10.564)
76-85:other	339	2.619 (0.667-10.288)
15-45:rural	3	2.082 (0.151-28.605)
46-55:rural	15	0.852 (0.139-5.226)
56-65:rural	16	0.498 (0.086-2.877)
66-75:rural	23	0.198 (0.033-1.201).
76-85:rural	21	0.458 (0.084-2.489)
**Age : Health Service**		
15-45:Atacama	165	1.499 (0.272-8.266)
46-55:Atacama	190	3.070 (0.754-12.504)

56-65:Atacama	200	4.186 (1.083-16.180)*
66-75:Atacama	250	3.643 (0.966-13.735).
76-85:Atacama	160	2.142 (0.527-8.702)
15-45:Vaparaiso-SanAntonio	444	7.309 (1.409-37.927)*
46-55:Vaparaiso-SanAntonio	534	6.241 (1.550-25.126)**
56-65:Vaparaiso-SanAntonio	744	4.404 (1.122-17.293)*
66-75:Vaparaiso-SanAntonio	802	3.026 (0.787-11.638)
76-85:Vaparaiso-SanAntonio	391	2.103 (0.509-8.686)
**Age : Referring Medical Center**		
15-45:tertiary	810	3.293 (0.890-12.188).
46-55:tertiary	926	4.351 (1.181-16.026)*
56-65:tertiary	1266	2.340 (0.909-6.019).
66-75:tertiary	1442	1.109 (0.519-2.370)
76-85:tertiary	834	1.307 (0.600-2.847)
**Sex : Health Service**		
Male:Atacama	387	1.627 (1.206-2.195)**
Male:Vaparaiso-SanAntonio	1236	1.101 (0.916-1.324)

***Significant at the 0.1% level, ** Significant at the 1% level, *Significant at the 5% level.

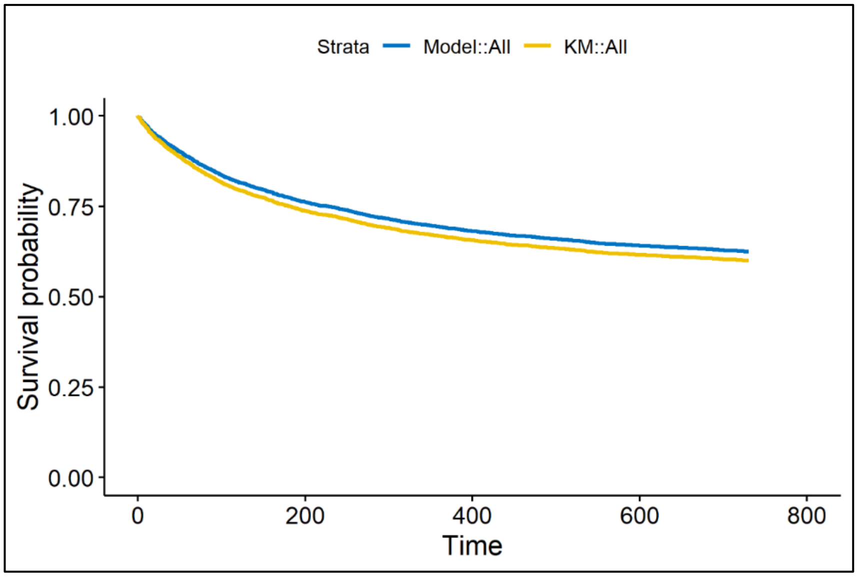

Figure S1. Kaplan Meier curve estimator (yellow) and Cox proportional model (blue) fitted to Oncology specialty. Time in calendar days.

A

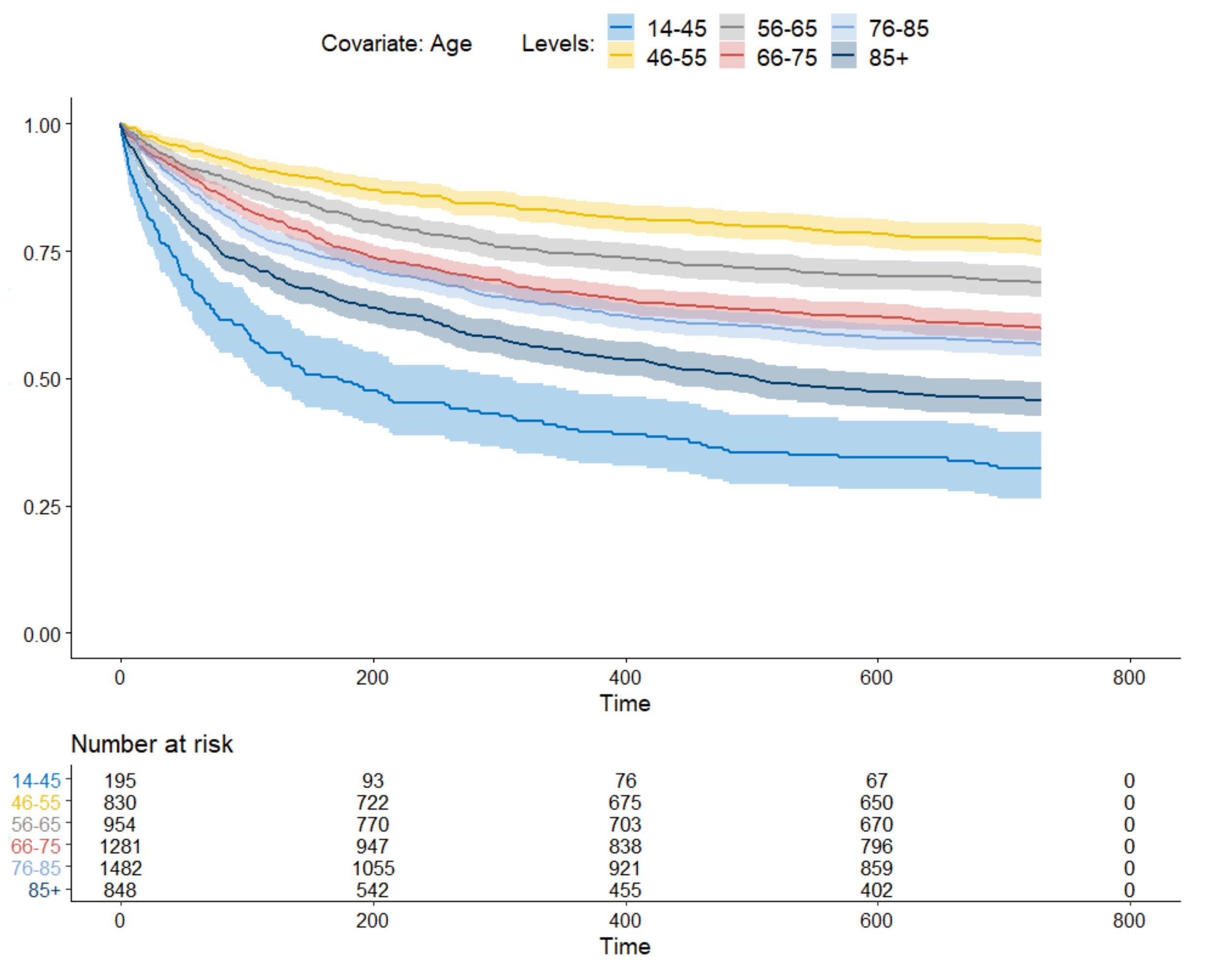
Covariate: Age
Levels:
14-45
46-55
56-65
66-75
76-85
85+
1.00
0.75
0.50
0.25
0.00
0
200
400
600
800
Time
Number at risk
14-45 195 93 76 67 0
46-55 830 722 675 650 0
56-65 954 770 703 670 0
66-75 1281 947 838 796 0
76-85 1482 1055 921 859 0
85+ 848 542 455 402 0
0
200
400
600
800
Time

B

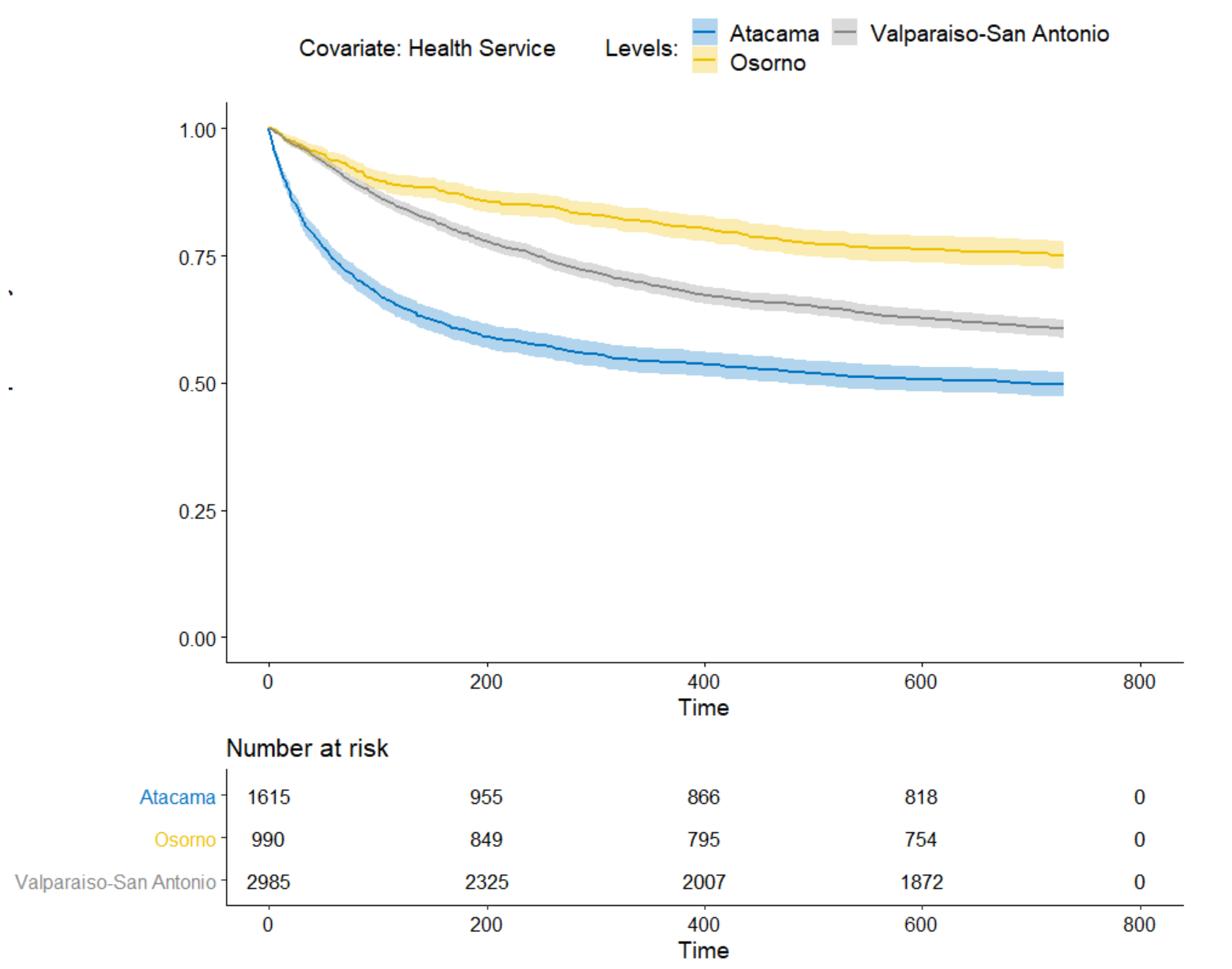
Covariate: Health Service
Levels:
Atacama
Valparaiso-San Antonio
Osorno
1.00
0.75
0.50
0.25
0.00
0
200
400
600
800
Time
Number at risk
Atacama
1615
955
866
818
0
Osorno
990
849
795
754
0
Valparaiso-San Antonio
2985
2325
2007
1872
0
0
200
400
600
800
Time

C

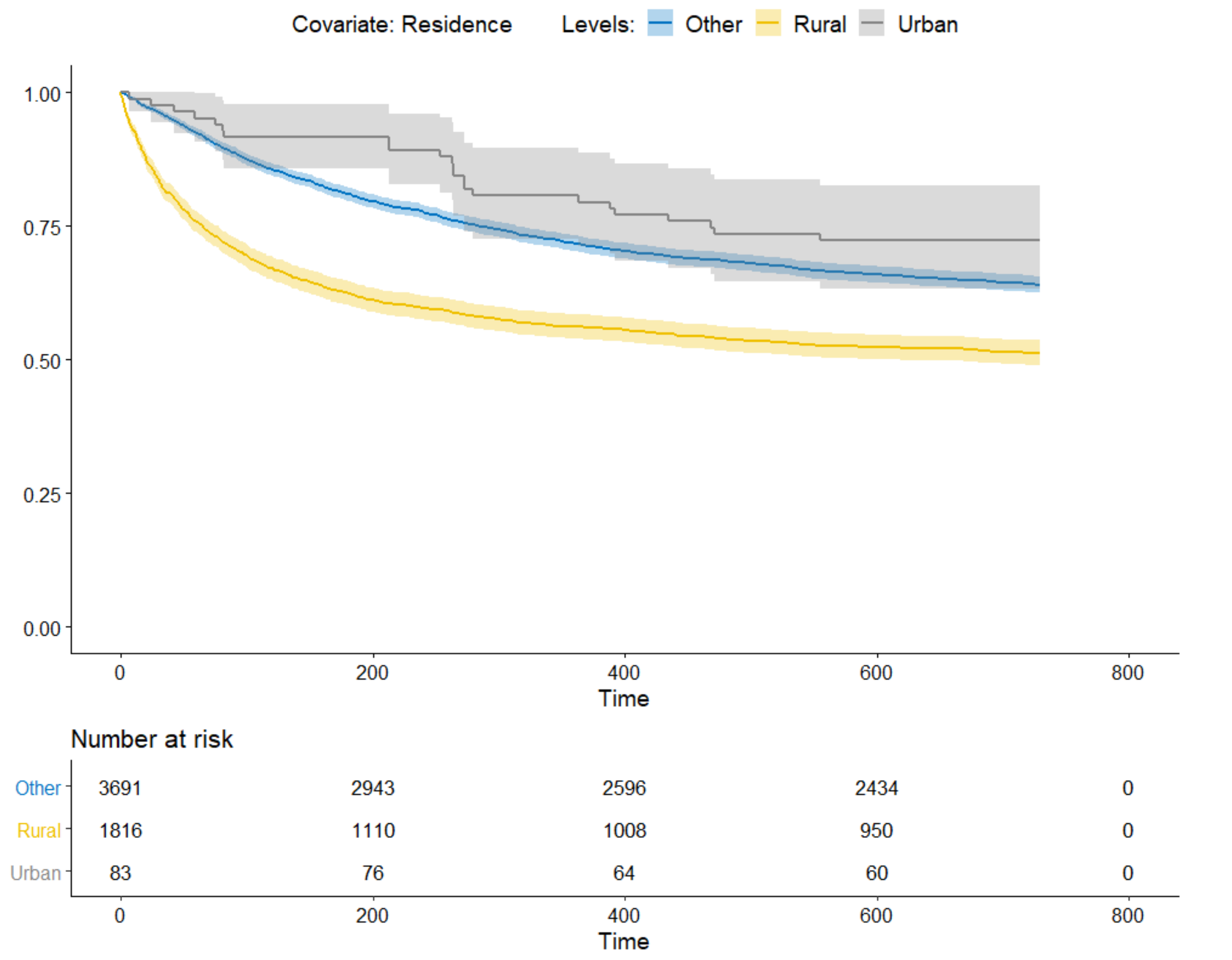
Covariate: Residence
Levels: Other Rural Urban
1.00
0.75
0.50
0.25
0.00
0
200
400
600
800
Time
Number at risk
Other 3691 2943 2596 2434 0
Rural 1816 1110 1008 950 0
Urban 83 76 64 60 0
0
200
400
600
800
Time

D

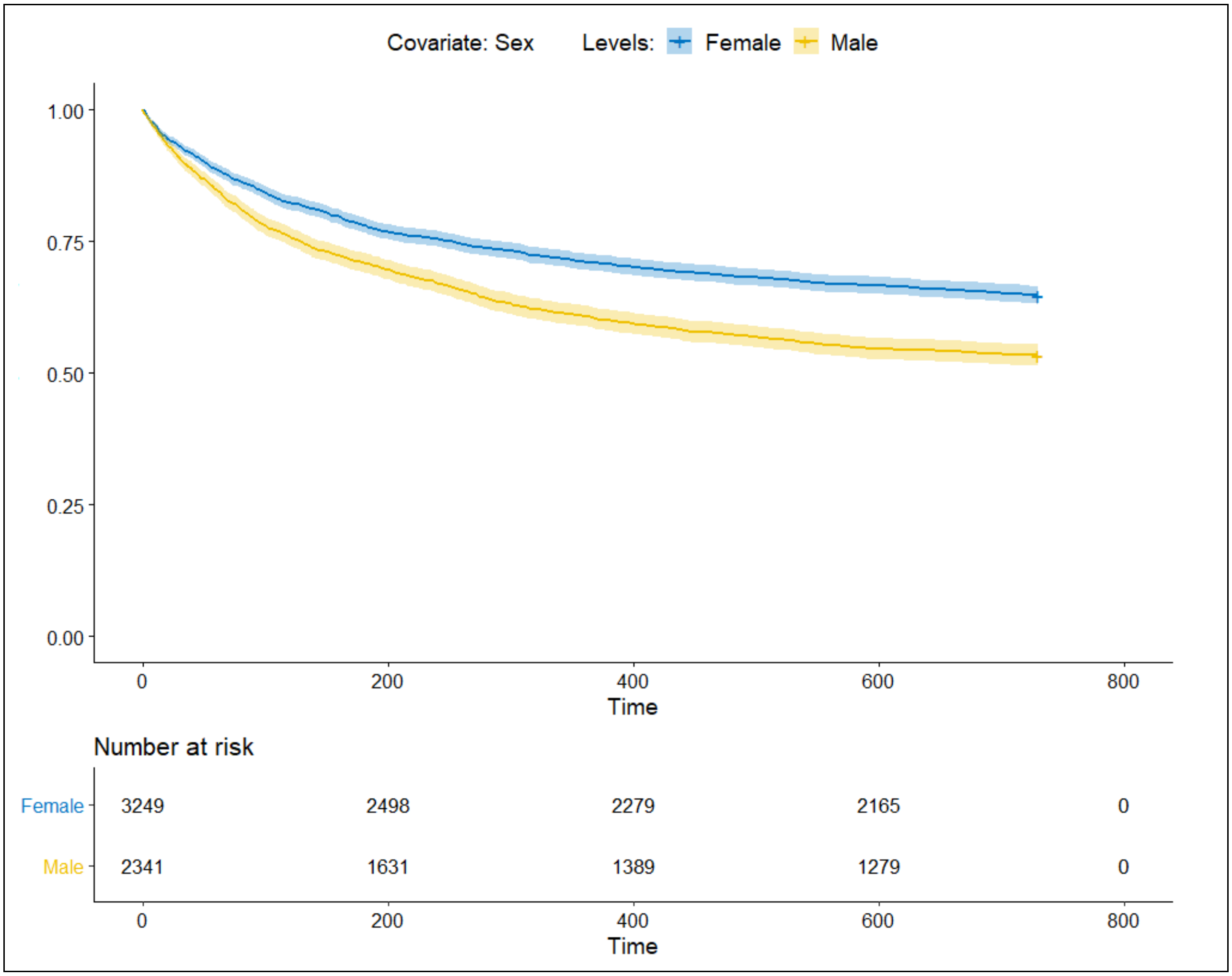

Figure S2. Kaplan Meier curve estimators for Oncology specialty model's covariates. Panel A by age group, panel B by Regional Health Service, panel C by patient's residence, and panel D by sex. Time in calendar days.

Survival Analysis on 2-, 2.5-, and 3-Year Mortality

It is possible that our effects are underestimated due to our main outcome defined as 2-year mortality. We investigate further by re-defining our outcome and estimating odds ratios for each of the covariates under study. Table S3 below present the hazard ratio for mortality within 2, 2.5, and 3 years of listing. The main patterns presented and discussed in the main manuscript remain.

Table S3. Hazard Risk Comparison for Mortality Within 2, 2.5, and 3 Years of Listing According to Covariates.

	N (%)	Overall 2 Year Mortality† HR (95% CI)	Overall 2.5 Year Mortality† HR (95% CI)	Overall 3 Year Mortality† HR (95% CI)
Age (15 to 45 comparator)				
0 to 3	68,028 (7)	0.76 (0.69 to 0.84)***	0.73 (0.66 to 0.8)***	0.7 (0.64 to 0.77)***
4 to 7	53,865 (5)	0.16 (0.12 to 0.2)***	0.16 (0.13 to 0.2)***	0.15 (0.12 to 0.19)***
8 to 11	43,053 (4)	0.1 (0.07 to 0.14)***	0.1 (0.08 to 0.14)***	0.12 (0.09 to 0.15)***
12 to 14	35,723 (4)	0.18 (0.14 to 0.24)***	0.19 (0.14 to 0.24)***	0.2 (0.16 to 0.26)***
15 to 45	293,892 (30)			
46 to 55	146,814 (15)	2.88 (2.72 to 3.05)***	2.88 (2.73 to 3.05)***	2.93 (2.78 to 3.09)***
56 to 65	139,099 (14)	5.47 (5.19 to 5.76)***	5.54 (5.27 to 5.82)***	5.67 (5.41 to 5.94)***
66 to 75	122,319 (12)	9.11 (8.66 to 9.57)***	9.41 (8.98 to 9.87)***	9.76 (9.33 to 10.21)***
76 to 85	70,835 (7)	16.11 (15.32 to 16.94)***	16.95 (16.16 to 17.77)***	17.63 (16.85 to 18.44)***
85+	13,869 (1)	31.77 (30.01 to 33.63)***	32.95 (31.22 to 34.77)***	34.22 (32.5 to 36.02)***
Sex (Female comparator)				
Female	613,499 (62)			
Male	373,998 (38)	1.65 (1.61 to 1.69)***	1.64 (1.6 to 1.67)***	1.63 (1.6 to 1.66)***
Residence (Other comparator)				
Rural	20,271 (2)			
Other	320,563 (32)	1.72 (1.56 to 1.89)***	1.68 (1.53 to 1.84)***	1.62 (1.49 to 1.76)***
Urban	646,663 (65)	1.19 (1.09 to 1.31)***	1.18 (1.08 to 1.28)***	1.14 (1.05 to 1.23)**
Health Service (Atacama comparator)				
Atacama	264,756 (27)			
Osorno	457,928 (46)	Std Dev=0.02	Std Dev=0.02	Std Dev=0.02
Valparaiso-San Antonio	264,813 (27)			
Health Insurance (Public comparator)				
Public	979,666 (99)			
Other (Private, Military)	7,831 (1)	0.85 (0.73 to 0.99)*	0.84 (0.73 to 0.97)*	0.84 (0.73 to 0.97)*
Specialty (Internal Medicine comparator)				
Internal Medicine	46,767 (5)			
Adult Surgery	71,148 (7)	0.67 (0.64 to 0.7)***	0.68 (0.65 to 0.71)***	0.68 (0.66 to 0.71)***
Anesthesiology	3,978 (0)	0.38 (0.33 to 0.44)***	0.39 (0.34 to 0.45)***	0.4 (0.36 to 0.46)***
Breast Surgery	1,478 (0)	0.55 (0.39 to 0.77)***	0.62 (0.46 to 0.84)**	0.68 (0.51 to 0.89)**
Bronchopulmonary	17,427 (2)	1.17 (1.1 to 1.24)***	1.19 (1.12 to 1.26)***	1.19 (1.13 to 1.25)***
Cardiology	31,480 (3)	0.66 (0.63 to 0.7)***	0.69 (0.65 to 0.72)***	0.71 (0.67 to 0.74)***
Cardiovascular Surgery	15,197 (2)	0.63 (0.58 to 0.68)***	0.67 (0.63 to 0.72)***	0.68 (0.64 to 0.73)***
Dentistry	128,505 (13)	0.33 (0.31 to 0.36)***	0.34 (0.32 to 0.37)***	0.36 (0.33 to 0.38)***
Dermatology	28,987 (3)	0.42 (0.38 to 0.46)***	0.44 (0.4 to 0.48)***	0.45 (0.42 to 0.49)***
Endocrinology	17,854 (2)	0.43 (0.39 to 0.49)***	0.48 (0.44 to 0.54)***	0.5 (0.46 to 0.55)***
Family Medicine	150 (0)	0.19 (0.03 to 1.32)	0.33 (0.08 to 1.31)	0.44 (0.14 to 1.37)
Gastroenterology	25,745 (3)	1.03 (0.98 to 1.09)	1.03 (0.97 to 1.08)	1.01 (0.96 to 1.06)
Genetics	3,348 (0)	0.64 (0.46 to 0.89)**	0.68 (0.5 to 0.91)*	0.66 (0.5 to 0.89)**

Hematology	5,868 (1)	1.6 (1.49 to 1.73)***	1.61 (1.5 to 1.73)***	1.58 (1.47 to 1.7)***
Infectious Disease	3,304 (0)	0.86 (0.7 to 1.05)	0.89 (0.74 to 1.08)	0.92 (0.77 to 1.1)
Maxillofacial Surgery	18,140 (2)	0.37 (0.31 to 0.43)***	0.36 (0.31 to 0.42)***	0.36 (0.31 to 0.42)***
Neonatology	282 (0)	1.61 (0.84 to 3.12)	1.58 (0.82 to 3.05)	1.55 (0.8 to 2.99)
Nephrology	11,208 (1)	1.02 (0.96 to 1.1)	1.06 (1 to 1.13)	1.08 (1.01 to 1.15)*
Neurology	47,087 (5)	0.82 (0.78 to 0.86)***	0.84 (0.8 to 0.89)***	0.85 (0.81 to 0.89)***
Neurosurgery	15,238 (2)	0.5 (0.46 to 0.55)***	0.51 (0.47 to 0.56)***	0.5 (0.46 to 0.55)***
Nutrition	1,856 (0)	2.16 (1.81 to 2.57)***	2.2 (1.86 to 2.6)***	2.2 (1.87 to 2.59)***
Obstetrics & Gynecology	93,979 (10)	0.42 (0.39 to 0.45)***	0.43 (0.4 to 0.46)***	0.44 (0.41 to 0.47)***
Oncology	6,080 (1)	3.57 (3.4 to 3.76)***	3.5 (3.33 to 3.67)***	3.37 (3.21 to 3.54)***
Ophthalmology	113,848 (12)	0.34 (0.32 to 0.36)***	0.38 (0.36 to 0.39)***	0.4 (0.38 to 0.41)***
Other	781 (0)	1.04 (0.76 to 1.44)	1.09 (0.81 to 1.47)	0.99 (0.73 to 1.34)
Otorhinolaryngology	67,646 (7)	0.43 (0.4 to 0.45)***	0.45 (0.43 to 0.47)***	0.47 (0.44 to 0.49)***
Pediatrics	18,177 (2)	0.41 (0.31 to 0.54)***	0.45 (0.35 to 0.58)***	0.46 (0.37 to 0.59)***
Physical Medicine & Rehabilitation	10,429 (1)	0.59 (0.53 to 0.65)***	0.62 (0.56 to 0.68)***	0.64 (0.58 to 0.7)***
Plastic Surgery	1,858 (0)	0.53 (0.43 to 0.67)***	0.56 (0.46 to 0.69)***	0.56 (0.46 to 0.69)***
Colorectal Surgery	3,114 (0)	0.57 (0.47 to 0.69)***	0.59 (0.49 to 0.7)***	0.57 (0.48 to 0.67)***
Psychiatry	13,135 (1)	0.74 (0.65 to 0.85)***	0.82 (0.73 to 0.93)**	0.86 (0.77 to 0.96)**
Rheumatology	7,341 (1)	0.4 (0.35 to 0.47)***	0.42 (0.36 to 0.47)***	0.42 (0.38 to 0.48)***
Sexual Transmitted Disease	1,807 (0)	0.3 (0.17 to 0.53)***	0.34 (0.2 to 0.56)***	0.33 (0.2 to 0.54)***
Traumatology	107,111 (11)	0.34 (0.32 to 0.36)***	0.37 (0.35 to 0.39)***	0.39 (0.37 to 0.4)***
Urology	47,102 (5)	0.58 (0.55 to 0.61)***	0.59 (0.56 to 0.62)***	0.6 (0.57 to 0.62)***
Referring Medical Center (Primary comparator)				
Primary	463,119 (47)			
Secondary	3,455 (0)	0.74 (0.52 to 1.04)	0.68 (0.49 to 0.94)*	2.02 (1.97 to 2.07)***
Tertiary	685,483 (69)	2.2 (2.14 to 2.26)***	2.09 (2.04 to 2.15)***	0.68 (0.5 to 0.92)*
Accepting Medical Center (Primary comparator)				
Primary	29,622 (3)			
Secondary	59,672 (6)	Std Dev=0.79	Std Dev=0.88	Std Dev=0.97
Tertiary	898,203 (91)			

Abbreviations: N, number of registrants; HR, hazard ratio; CI, confidence interval; Std Dev, standard deviation.

†Results are from mixed-effects Cox proportional hazard models with Health Service & Accepting Medical Center included as a crossed random effect.

***Significant at the 0.1% level, ** Significant at the 1% level, *Significant at the 5% level.

Correlation Analysis by Level of Care

It might be possible the positive correlation we found between waiting variability and mortality is overestimated due to the inclusion of all medical center types (primary, secondary, and tertiary) into the correlation analysis discussed in the main manuscript. Here, we investigate the association between waiting and mortality further by focusing only in primary care centers. Figure S3 shows no strong positive correlation between center-specific mortality risk and waiting time performance for secondary and tertiary medical centers.

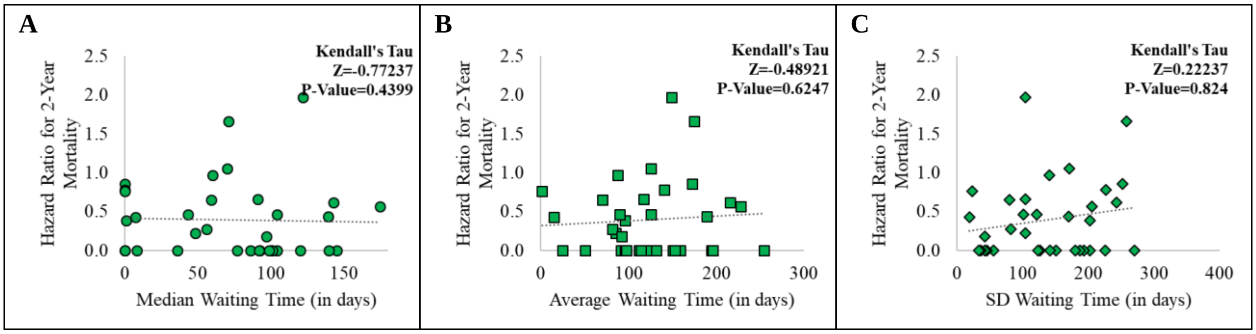

Figure S3. Association between primary medical center-specific hazard ratio (HR) for death and primary care center-specific waiting time performance (Panel A, median waiting time against HR; Panel B, average waiting time against HR; Panel C, standard deviation of waiting time against HR). Kendall rank correlation coefficient measured no statistically significant association between the medical centers' waiting times and the medical centers' HR for death. Medical centers with <30 patients in their waiting lists during the study timeframe were excluded from the analyses. Abbreviations: SD, standard deviation.

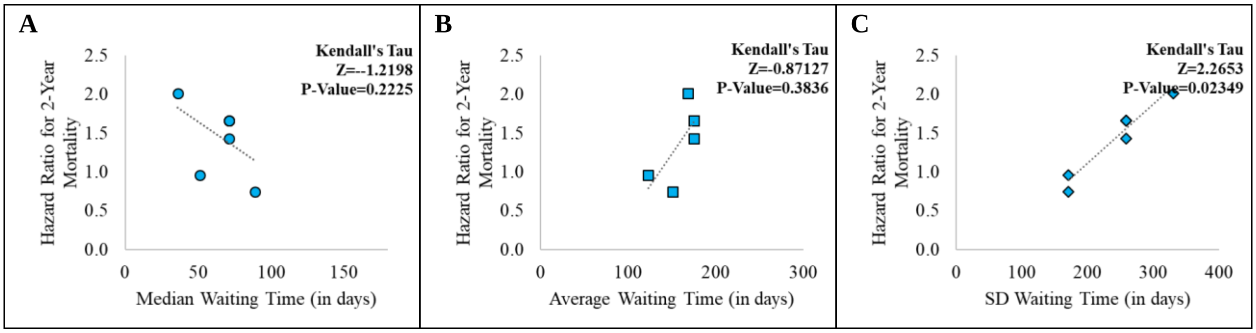

Figure S3. Association between secondary and tertiary medical center-specific hazard ratio (HR) for death and medical center-specific waiting time performance (Panel A, median waiting time against HR; Panel B, average waiting time against HR; Panel C, standard deviation of waiting time against HR). Kendall rank correlation coefficient measured no statistically significant association between the medical centers' median and average waiting times and the medical centers' HR for death (Panel A and B). The same examination found a statistically significant positive correlation between the medical centers' standard deviation of waiting time and the medical centers' HR for death (Panel C). Medical centers with <30 patients in their waiting lists during the study timeframe were excluded from the analyses. Abbreviations: SD, standard deviation.

Correlation Analysis for High-Risk Medical Specialties

It might be possible that the positive correlation we found between waiting variability and mortality is underestimated due to the inclusion of all medical specialties. Here, we investigate the association between center-specific waiting and mortality focusing only in high-risk specialties (mortality rate over 50th percentile). As presented in Figure S4, for the subset of high mortality and high waiting time variability specialties, we found a strong positive correlation between waiting time and mortality.

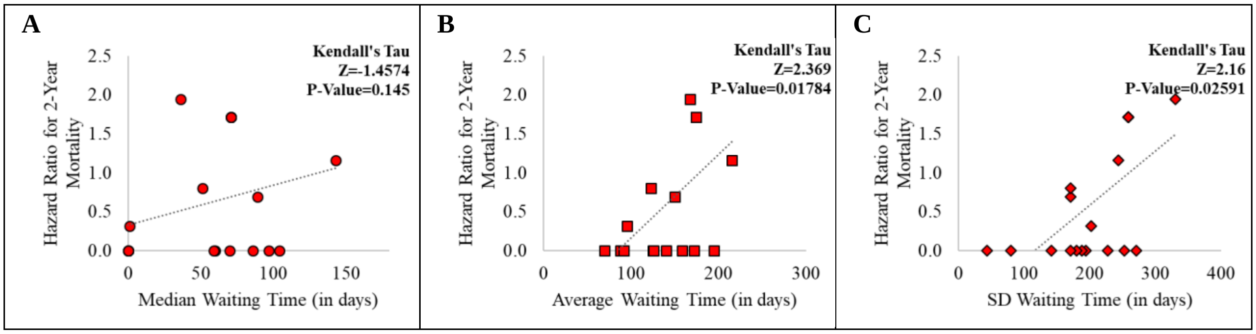

Figure S4. Association between medical center-specific hazard ratio (HR) for death and medical center-specific waiting time performance (Panel A, median waiting time against HR; Panel B, average waiting time against HR; Panel C, standard deviation of waiting time against HR). Kendall rank correlation coefficient measured statistically significant associations between the medical centers' waiting time performance and mortality risk. Medical centers with <30 patients in their waiting lists during the study timeframe were excluded from the analyses. Abbreviations: SD, standard deviation.

EXTENSIONS TO MAIN ANALYSIS

Methodological Justification of The Selected Study Design

The availability of each patient's waiting time could be used to study the association between waiting and death for patients not prioritized by the GES Act. However, such study design is problematic due to unobserved health status differences that are likely to confound our attempts to measure the effect of waiting on mortality. In initial data explorations presented in Table S4, we found patients who died had consistently shorter wait times, which can be explained by subconscious triage effect. To isolate the effect of waiting on mortality, we therefore measured the association at the medical center level. We calculate a mortality risk associated to each medical center, and then we measured the association between these mortality risks with the respective waiting time performance of each medical center. The results of the proposed medical center-specific analyses are presented in the main manuscript.

Table S4. Distribution of waiting times by specialty and mortality status

Specialty	Median Waiting Time		P-Value†
	Dead within two years	Alive	
Internal Medicine	37	13	<0.0001
Adult Surgery	45	77	<0.0001
Anesthesiology	21	21	0.7731
Breast Surgery	14	37	0.000933
Bronchopulmonary	27	25	0.0148
Cardiology	56	61	<0.0001
Cardiovascular Surgery	78	205.5	<0.0001
Dentistry	142	129	0.2984
Dermatology	40	47	0.0092
Endocrinology	41	51	0.0013
Family Medicine	25	55	NA (sample size = 1)
Gastroenterology	50	71	<0.0001
Genetics	51	84	0.0024
Hematology	21	21	0.2330
Infectious Disease	37	13	<0.0001
Maxillofacial Surgery	34	50	<0.0001
Neonatology	19	29	0.2379
Nephrology	78	60	0.0018
Neurology	94	76	0.4240
Neurosurgery	36	36	0.1309
Nutrition	34	31	0.1607
Obstetrics & Gynecology	31	41	<0.0001
Oncology	17	22	<0.0001
Ophthalmology	103	97	0.0245
Other	65	108	0.3403
Otorhinolaryngology	67.5	80	<0.0001
Pediatrics	51	35	0.3243
Physical Medicine & Rehabilitation	32	39	<0.0001
Plastic Surgery	35.5	49	0.0554
Colorectal Surgery	33	50	<0.0001
Psychiatry	45	49	0.0438
Rheumatology	43	42	0.8913
Sexual Transmitted Disease	104.5	58	0.3558
Traumatology	45	51	<0.0001
Urology	42	82	<0.0001

†Results are from Wilcoxon rank sum test with continuity correction

EXTRA DATA VISUALIZATIONS

Age- and Sex-Adjusted Mortality Rate

Age-adjusted and sex-adjusted mortality rates for our non-GES new specialty waitlist and each specific age cohort are presented in Figure S5, Figure S6, and Figure S7.

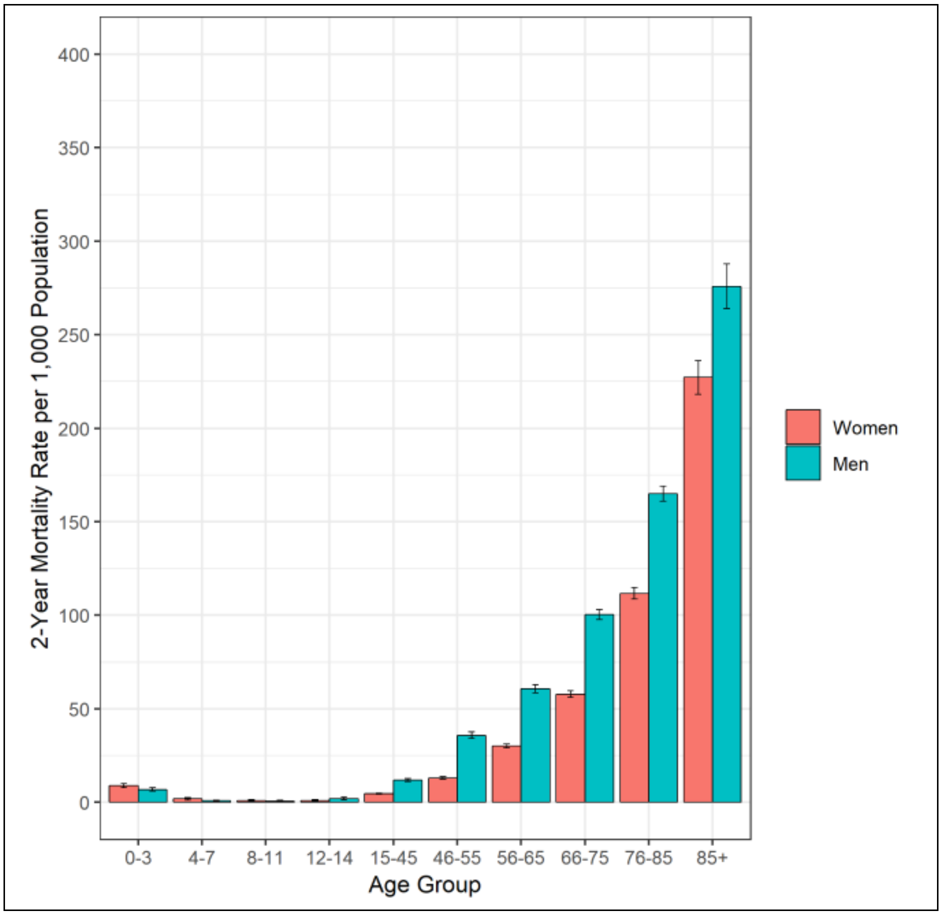

Figure S5. 2-year crude death rate by sex and age-groups in the non-GES waitlist for new specialty: Atacama, Valparaiso-San Antonio, and Osorno, 2008-2017. Error bars indicate 95% confidence intervals.

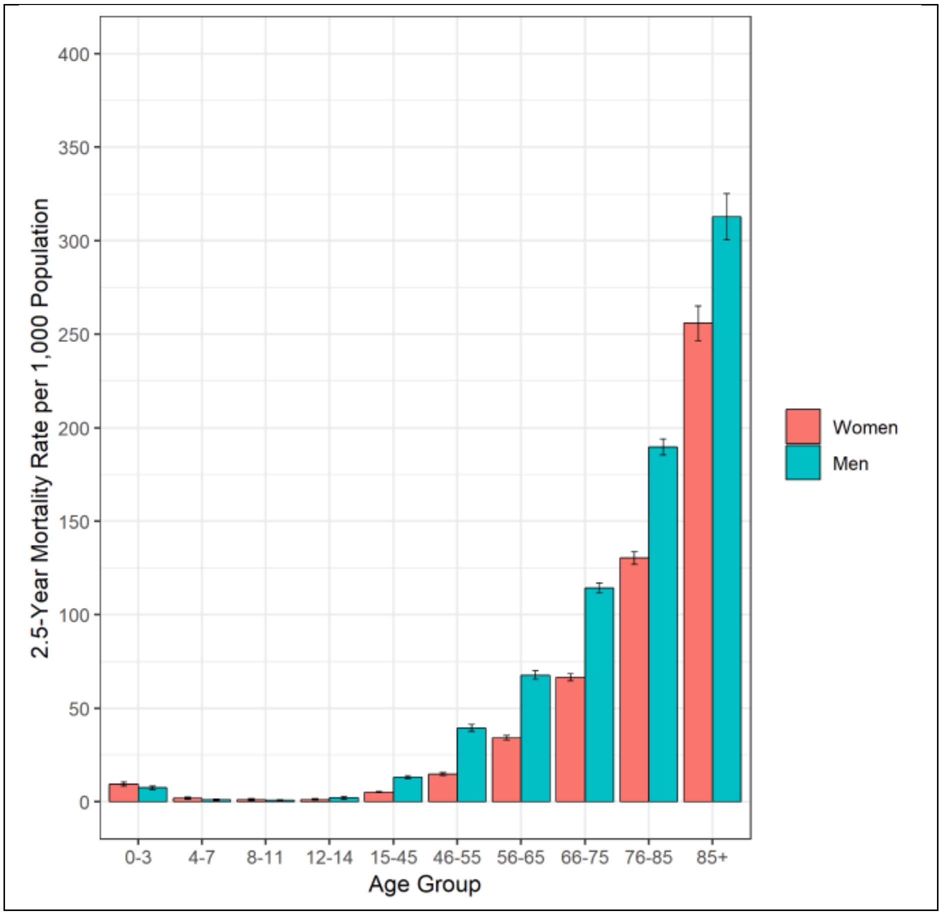

Figure S6. 2.5-year crude death rate by sex and age-groups in the non-GES waitlist for new specialty: Atacama, Valparaiso-San Antonio, and Osorno, 2008-2017. Error bars indicate 95% confidence intervals.

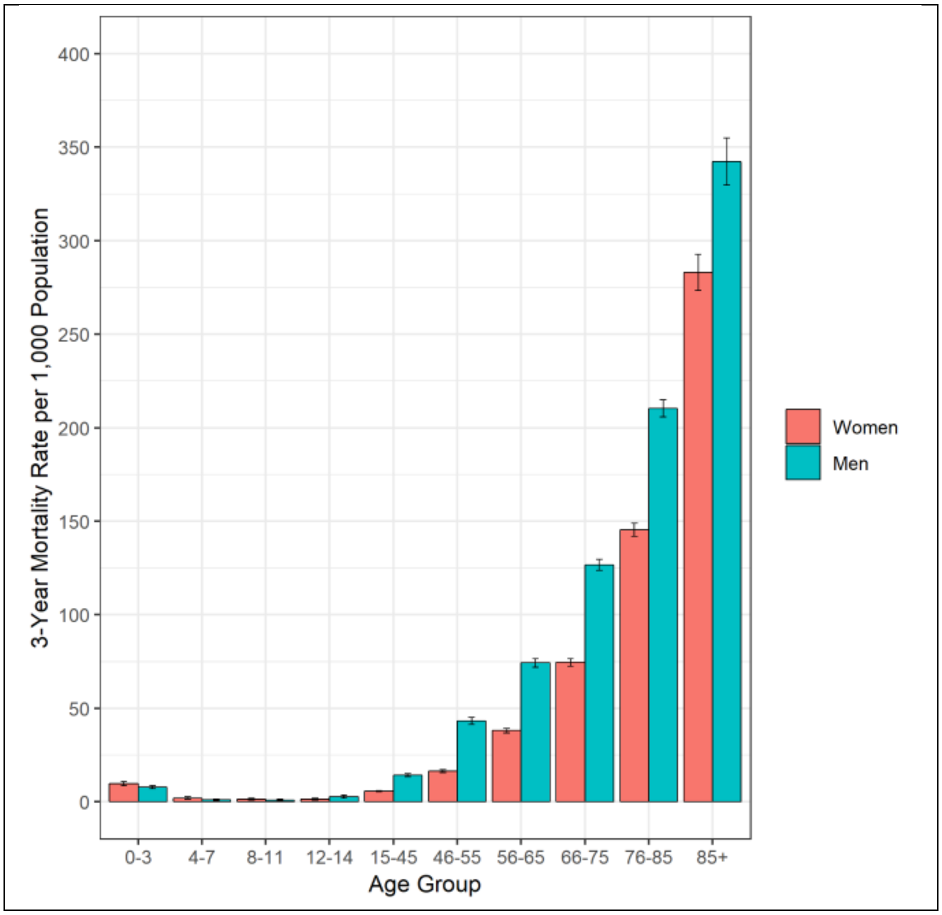

Figure S7. 3-year crude death rate by sex and age-groups in the non-GES waitlist for new specialty: Atacama, Valparaiso-San Antonio, and Osorno, 2008-2017. Error bars indicate 95% confidence intervals.

Specialty- and Sex-Adjusted Mortality Rates

Age-adjusted and sex-adjusted mortality rates for our non-GES new specialty waitlist and each specific age cohort are presented in Figure S8.

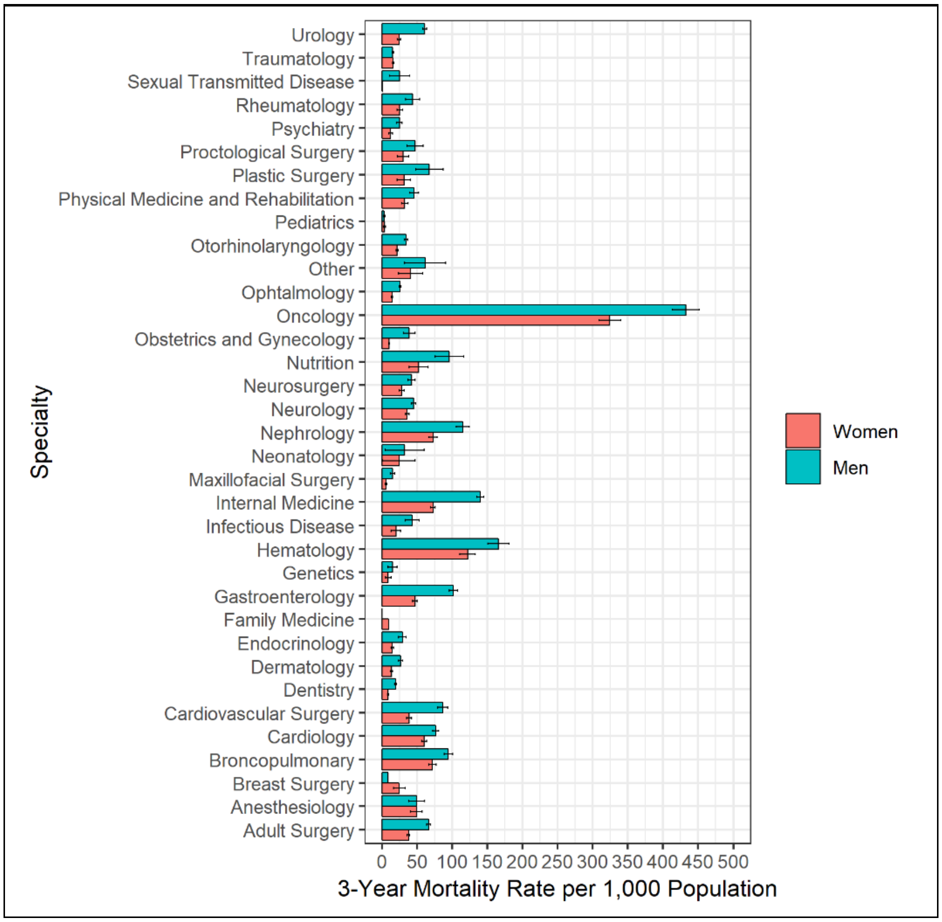

Figure S8. 2-year crude death rate by specialty and age-groups in the non-GES waitlist for new specialty: Atacama, Valparaiso-San Antonio, and Osorno, 2008-2017. Error bars indicate 95% confidence intervals.

Death-Adjusted Waiting Time Distribution

Death-adjusted waiting times for our non-GES new specialty waitlist are presented in Figure S9, S10, and S11.

Figure S9. Probability density estimation of the waiting time by 2-year death status for the non-GES waitlist for new specialty: Atacama, Valparaiso-San Antonio, and Osorno, 2008-2017.

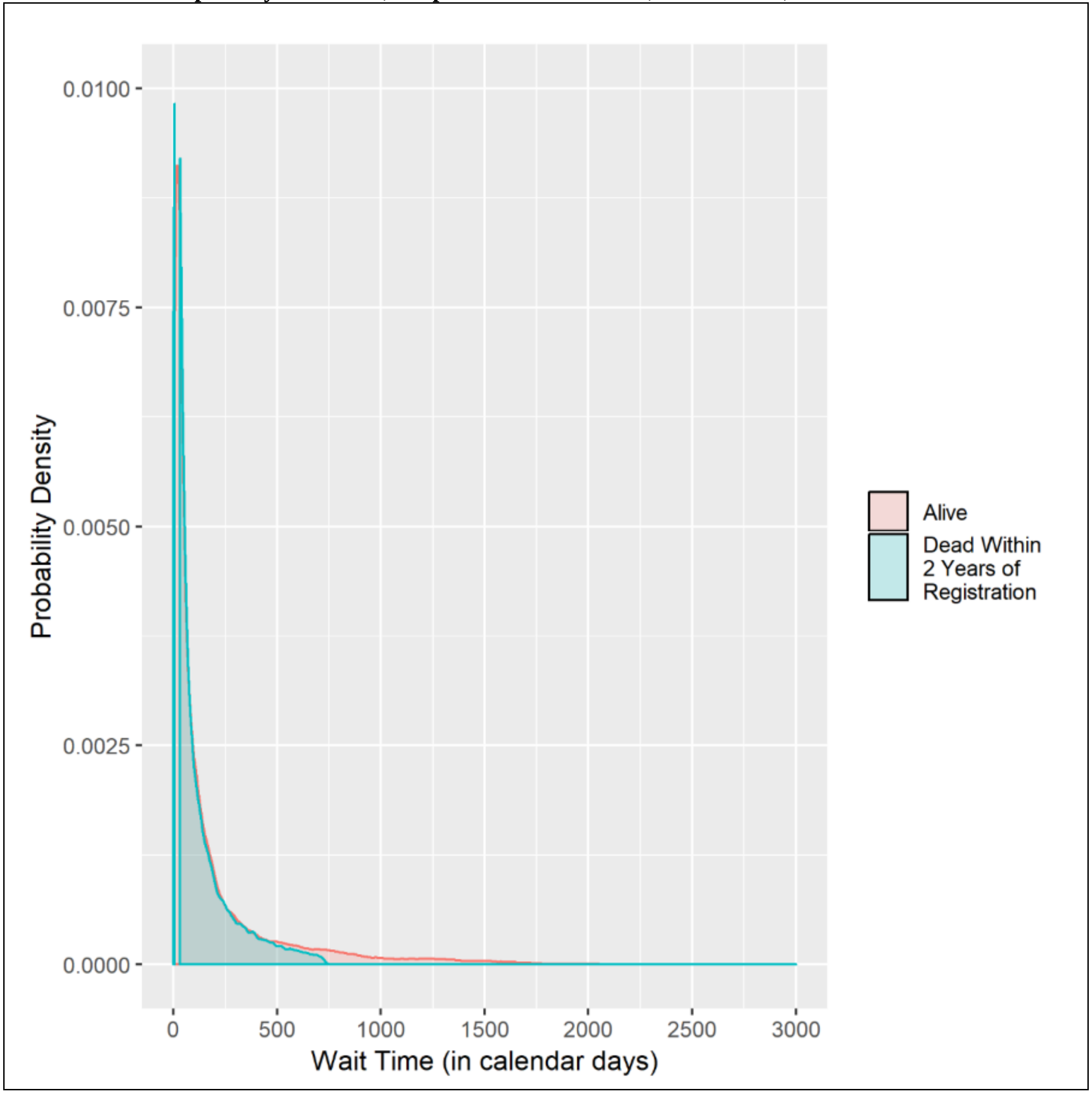

Figure S10. Probability density estimation of the waiting time by 2.5-year death status for the non-GES waitlist for new specialty: Atacama, Valparaiso-San Antonio, and Osorno, 2008-2017.

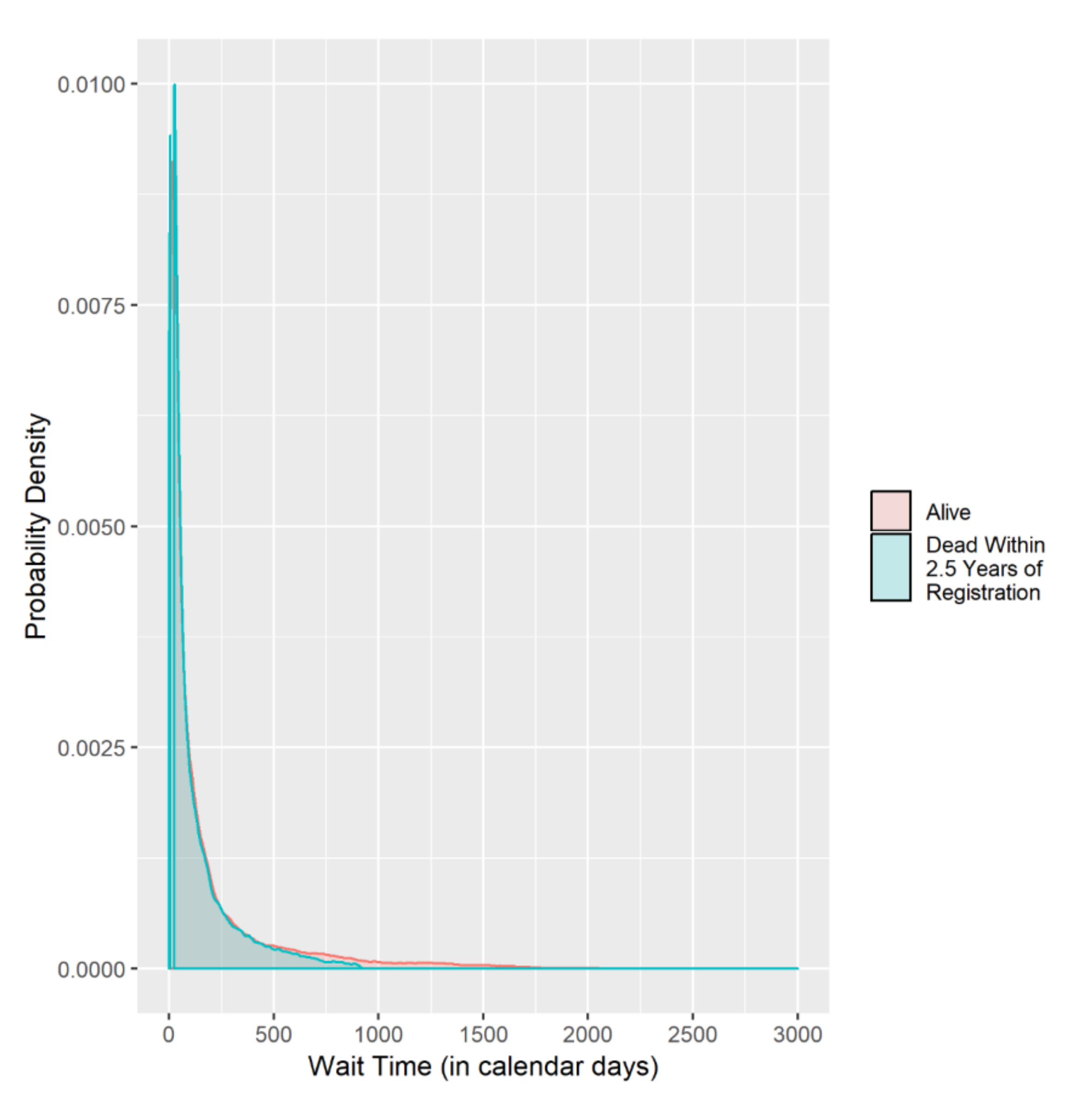

Figure S11. Probability density estimation of the waiting time by 3-year death status for the non-GES waitlist for new specialty: Atacama, Valparaiso-San Antonio, and Osorno, 2008-2017.

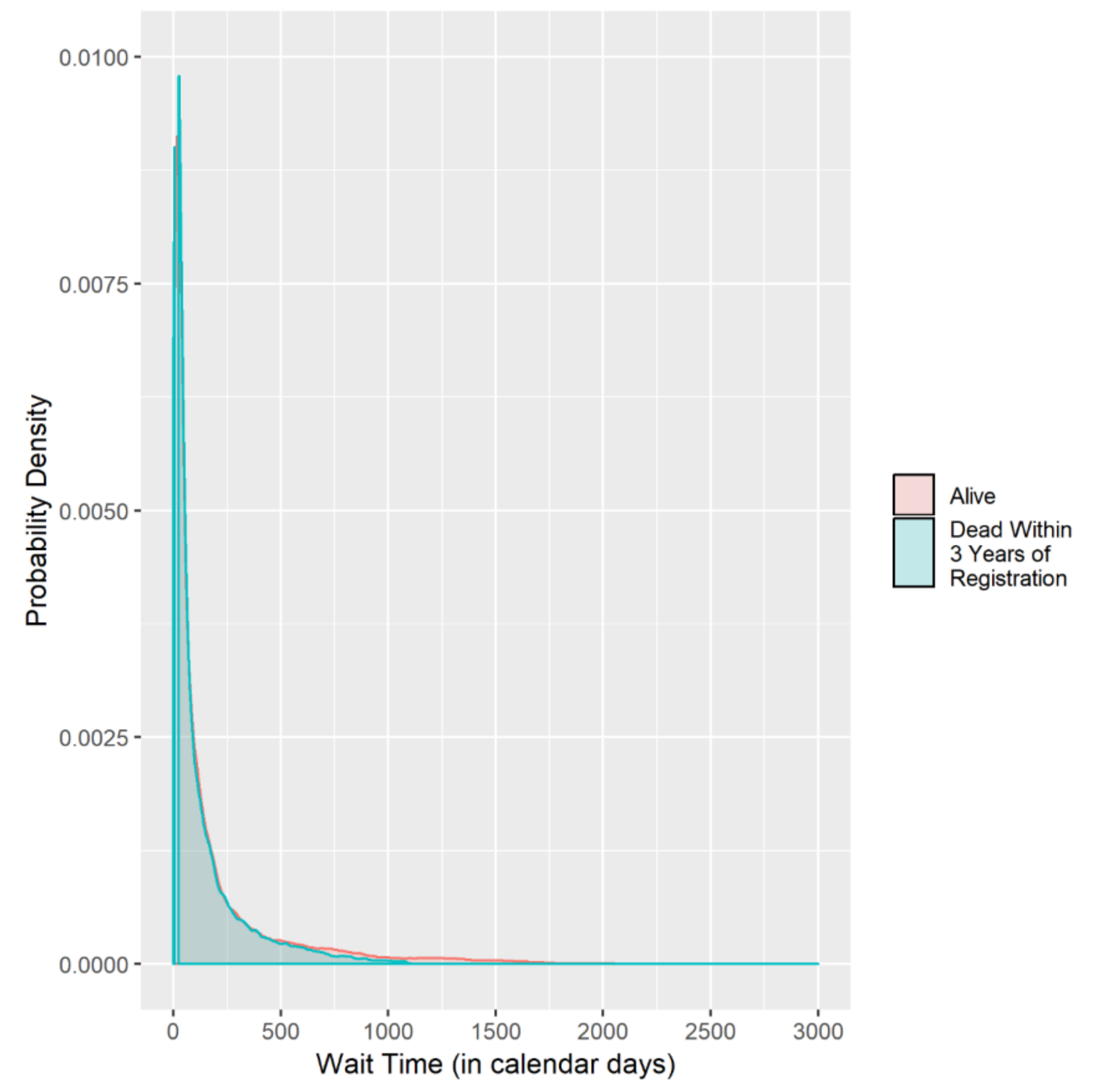

1

POLICY TIMELINE

Health Problems Prioritized by the GES Act

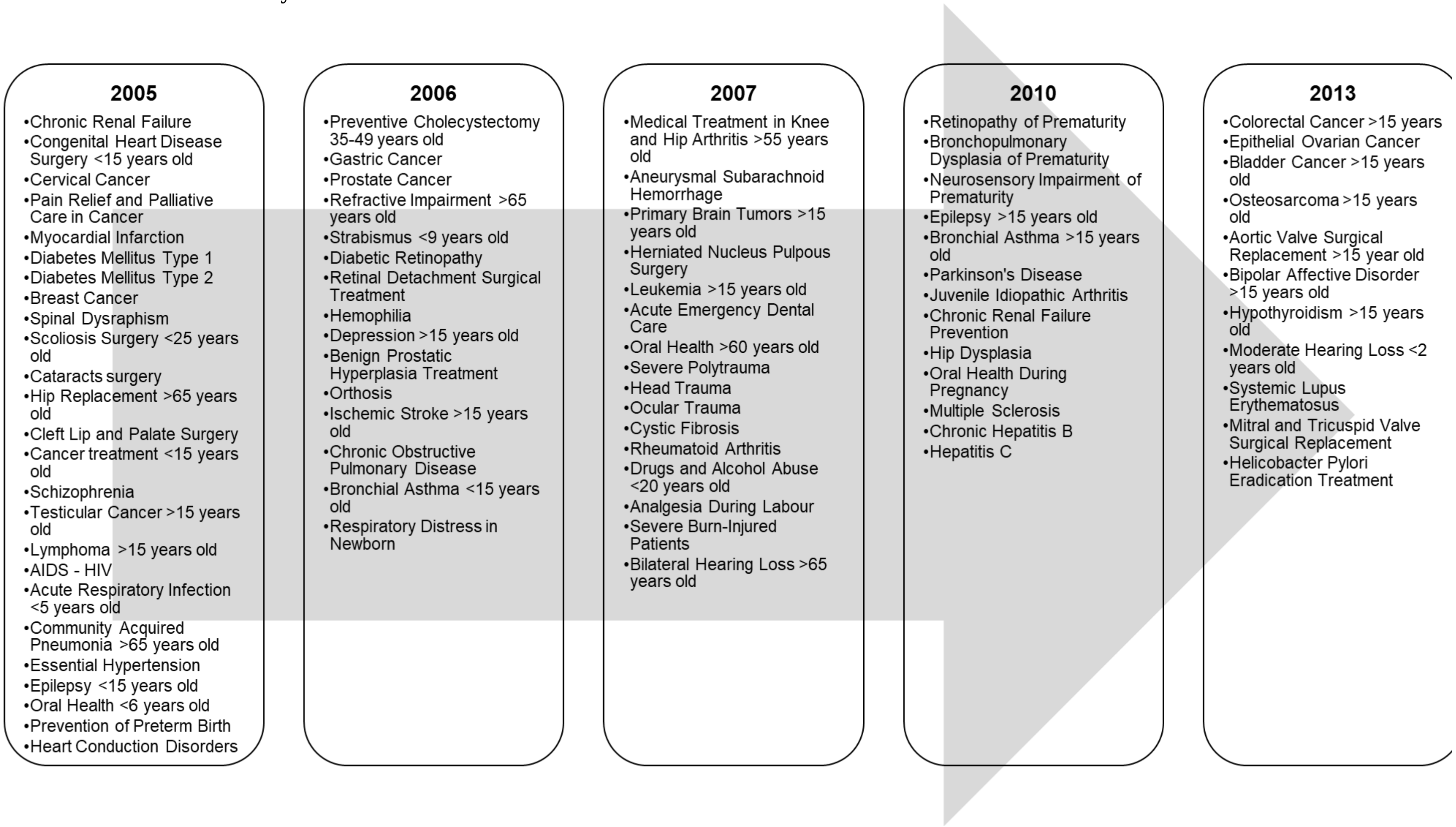

Figure S12. List of health problems prioritized the GES Act.

DATA COLLECTION AND ANALYSIS

Our group requested the non-GES new specialty waiting list data by means of the Chile Transparency Act on March 15, 2018. We requested patient-level data to all 33 Chilean Regional Health Services (RHS) through the Transparency Law website (https://portaltransparencia.cl/PortalPdT/). Our data request included:

- A masked unique identifier for each registrant,
- Date of birth,
- Sex,
- Type of health coverage,
- Type of service,
- Location of healthcare entity that creates the referral,
- Is the entity that creates the referral is a rural area?
- Location of healthcare entity where patient is referred,
- Specialty where the patient is referred,
- Waiting list input date,
- Waiting list output date,
- Reason for output from the waiting list,
- PRAIS coverage status,
- Suspected diagnosis,
- Confirmed diagnosis,
- Appointment date,
- Attendance to appointment status,
- Date of death,
- Alert, and
- Reason of the alert.

Out of the 33 requests, three RHS sent the requested datasets (Atacama, Valparaiso-San Antonio, and Osorno), three RHS sent datasets with data elements that were different from our original request, and the remaining 27 RHS either rejected the request or asked for an extension. Therefore, our analyses were based on the datasets shared by the RHS of Atacama, Valparaiso-San Antonio, and Osorno. The request codes assigned by the Transparency Law website are AO019T0000319 for Atacama, AO022T0000406 for Valparaíso-San Antonio, and AO034T0000266 for Osorno. Figure S13 depicts waitlist resgistrants collected and reasons for exclusion.

Figure S13. Registrant selection in the study of non-GES waitlist for new specialty: Atacama, Valparaiso, and Osorno, 2008-2017.

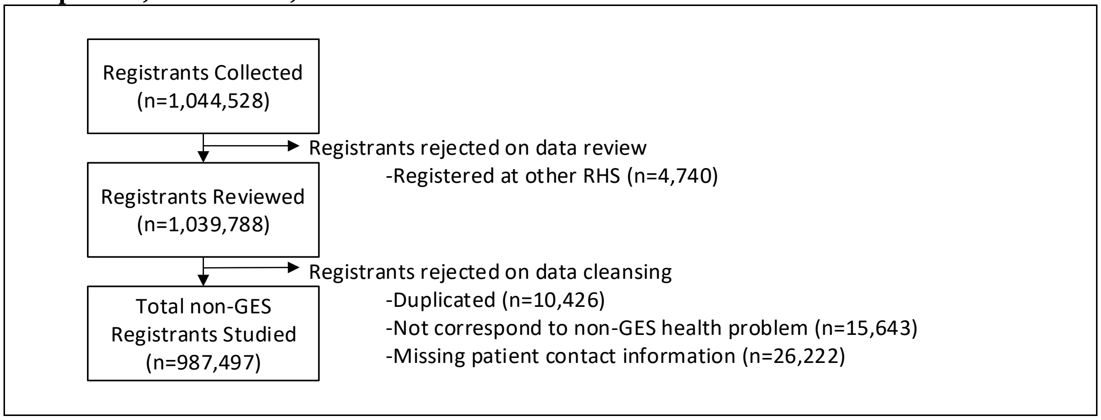

Table S5. Structure of the study sample				
**Year**	**Number of Cases**	**Number of Response**	**Response Rate**	**Attrition Rate**
2008	14,344	13,616	94.92%	5.08%
2009	34,487	34,274	99.38%	0.62%
2010	83,604	78,320	93.68%	6.32%
2011	146,839	140,131	95.43%	4.57%
2012	127,089	120,408	94.74%	5.26%
2013	119,317	113,882	95.44%	4.56%
2014	124,307	120,613	97.03%	2.97%
2015	139,651	134,729	96.48%	3.52%
2016	140,451	135,482	96.46%	3.54%
2017	99,273	96,042	96.75%	3.25%
**Overall**	**1,029,362**	**987,497**	**95.93%**	**4.07%**
