# Supplementary material for: Prolonged wait time is associated with increased mortality for Chilean waiting list patients with non-prioritized conditions
Source: BMC Public Health. 2019 Feb 26;19:233. doi: 10.1186/s12889-019-6526-6 (PMC6390314; doi:10.1186/s12889-019-6526-6)
Supplement: Supplementary file 2 — Data Cleaning Procedures. (PDF 116 kb) [file 12889_2019_6526_MOESM2_ESM.pdf]

Prolonged Wait Time is Associated with Increased Mortality for Chilean Waiting List Patients with Non-Prioritized Conditions

Diego A. Martinez, PhD,[1] Haoxiang Zhang, MSc,[1] Magdalena Bastias, PhD,[2] Felipe Feijoo,[3] PhD, Jeremiah Hinson, MD, PhD,[1] Rodrigo Martinez, MD,[2] Jocelyn Dunstan,[2] PhD, Scott Levin, PhD,[1] Diana Prieto, PhD[1]

[1]Johns Hopkins University
[2]University of Chile
[3]Pontifical Catholic University of Valparaíso

Additional File 2: Data Cleaning Procedures

```
#DATA CLEANING ATACAMA####
##1. LOAD R PACKAGES####
library(dplyr)
library(openxlsx)
library(lubridate)
library(stringr)
#############################################################################################################
```

```
#2. SET WORKING DIRECTORY####
setwd("UNIQUE TO THE USER RUNNING THIS PROCEDURE")
```

```
#3. LOAD DATASETS####
d1<-read.xlsx("./Raw Data/NO-GES_NewSpecialty_Atacama_2008.xlsx",sheet = 1,startRow = 2,colNames = F)
```

```
d2<-read.xlsx("./Raw Data/NO-GES_NewSpecialty_Atacama_2009.xlsx",sheet = 1,startRow = 2,colNames = F)
d3<-read.xlsx("./Raw Data/NO-GES_NewSpecialty_Atacama_2010.xlsx",sheet = 1,startRow = 2,colNames = F)
d4<-read.xlsx("./Raw Data/NO-GES_NewSpecialty_Atacama_2011.xlsx",sheet = 1,startRow = 2,colNames = F)
d5<-read.xlsx("./Raw Data/NO-GES_NewSpecialty_Atacama_2012.xlsx",sheet = 1,startRow = 2,colNames = F)
d6<-read.xlsx("./Raw Data/NO-GES_NewSpecialty_Atacama_2013.xlsx",sheet = 1,startRow = 2,colNames = F)
d7<-read.xlsx("./Raw Data/NO-GES_NewSpecialty_Atacama_2014.xlsx",sheet = 1,startRow = 2,colNames = F)
d8<-read.xlsx("./Raw Data/NO-GES_NewSpecialty_Atacama_2015.xlsx",sheet = 1,startRow = 2,colNames = F)
d9<-read.xlsx("./Raw Data/NO-GES_NewSpecialty_Atacama_2016.xlsx",sheet = 1,startRow = 2,colNames = F)
d10<-read.xlsx("./Raw Data/NO-GES_NewSpecialty_Atacama_2017.xlsx",sheet = 1,startRow = 2,colNames = F)
# merge
dirtydata<-rbind(d1,d2,d3,d4,d5,d6,d7,d8,d9,d10)
# rename
names<-c("id_orig","healthdept","birthdate","sex","insurance","health_service","PRESTA_MIN","areaofcare",
         "entrydate","ref_from","ref_to","exitdate","Dias_Espera","exit_reason","E_OTOR_AT",

"PRESTA_MIN_SALIDA","program","region","borough","suspect_diag","confirm_diag","city","rural_urban","apptd
ate","deathdate")
colnames(dirtydata)<-names
write.csv(dirtydata,file = "./Raw Data/dataset during cleanning/Atacama/merged dirty data.csv",row.names =
F)
```

```
#4. DATA CLEANING PROCEDURE####
# General Cleaning
dirtydata<-read.csv("./Raw Data/dataset during cleanning/Atacama/merged dirty data.csv")
head(dirtydata)
str(dirtydata)
dirty<- dirtydata %>%mutate(id_orig = as.character(id_orig),
                            healthdept = as.factor(healthdept),
                            birthdate = as.Date(birthdate,"%d/%m/%Y"),
                            sex =
as.factor(ifelse(sex==1,"Male",ifelse(sex==2,"Female",ifelse(sex==3,"Indetermined",ifelse(dirtydata$sex==4
,"Unknown",NA))))),
                            insurance=
as.factor(ifelse(insurance==1,"public",ifelse(insurance==2,"private",ifelse(insurance==3,"Independent",NA)
))),
                            health_service = as.factor(health_service),
                            borough = as.factor(borough),
                            ref_from = as.factor(ref_from),
                            ref_to = as.factor(ref_to),
                            exit_reason = as.factor(exit_reason),
                            rural_urban =
ifelse(rural_urban==1,"urban",ifelse(rural_urban==2,"rural","other")),
                            entrydate = as.Date(entrydate,"%d/%m/%Y"),
                            year_entry = year(entrydate),
                            month_entry = month(entrydate),
                            exitdate = as.Date(exitdate,"%d/%m/%Y"),
                            program = as.factor(program),
                            city = as.factor(city),
                            region = as.factor(region),
                            Dias_Espera = as.factor(Dias_Espera),
```

```
                            apptdate = as.Date(apptdate,"%d/%m/%Y"),
                            deathdate = as.Date(deathdate,"%d/%m/%Y"),
                            wtime = as.numeric(exitdate - entrydate),
                            age = as.numeric(floor((entrydate-birthdate)/365)),
                            WT = as.factor(ifelse(wtime<31,"0-30",
                                                  ifelse(wtime<61,"31-60",ifelse(wtime<181,"61-180",

ifelse(wtime>180,"181+",NA))))),
                            WT2 = as.factor(ifelse(wtime<90,"<90",ifelse(wtime<121,"90-120",">120"))),
                            Age = as.factor(ifelse(age<15,"<15",
                                                   ifelse(age<46,"15-45",ifelse(age<56,"46-
55",ifelse(age<66,"56-65",

ifelse(age<76,"66-75",ifelse(age<86,"76-85",ifelse(age>85,"85+",NA)))))))),
                            death = ifelse(is.na(deathdate),0,1))
summary(dirty)

#Exclude
## exclude data_exit missing
dirty<-dirty[which(!is.na(dirty$exitdate)),]
## exclude age<0
dirty<-dirty[which(dirty$age>=0),]
## exclude sex==indetermined
dirty<-dirty[which(!(dirty$sex%in%c("Indetermined","Unknown"))),]
## rural urban
dirty$rural_urban[which(is.na(dirty$rural_urban))]<-"other"
dirty$rural_urban<-as.factor(dirty$rural_urban)
## WT and Age
dirty$WT<-ordered(dirty$WT,levels=c("0-30","31-60","61-180","181+"))
dirty$WT2<-ordered(dirty$WT2,levels=c("<90","90-120",">120"))
dirty$Age<-ordered(dirty$Age,levels=c("<15","15-45","46-55","56-65","66-75","76-85","85+"))
## Medical Service
dirty$areaofcare<-gsub("Ã-","i",dirty$areaofcare)
dirty$areaofcare<-gsub("Ã¡","a",dirty$areaofcare)
dirty$areaofcare<-gsub("Ã³","o",dirty$areaofcare)
dirty$areaofcare<-gsub("Ã©","e",dirty$areaofcare)
dirty$areaofcare<-gsub("Ã�","I",dirty$areaofcare)
dirty$areaofcare<-gsub("Ã�","O",dirty$areaofcare)
dirty$areaofcare<-str_to_title(dirty$areaofcare)
dirty$areaofcare<-as.factor(dirty$areaofcare)
## Exit Reason
levels(dirty$exit_reason)<-c("Ges","Completed Cases","Informed Procedure??","Medical
Revaluation","Completed Cases Externally??","Health Coverage Change",
                             "Patient Reject","Patient Spontaneous Recovery","Patient
Missed","Death","Duplicated Request??","Completed Case by Resolutivity",
                             "Completed Case by Telemedicine","Clinical Case Change","Contact Doesn't
Match??","Surgery Not Necessary","Transfer","No pertinencia??",
                             "Digitation Error")
cleanv1<-dirty
summary(cleanv1)
write.csv(cleanv1,"./Raw Data/dataset during cleanning/Atacama/cleanv1.csv",row.names = F)
# Clean    ref_from (E_OTOR_AT)  ,   ref_to   ,   areaofcare
cleanv1<-read.csv("./Raw Data/dataset during cleanning/Atacama/cleanv1.csv")
cleanv1$E_OTOR_AT<-as.factor(cleanv1$E_OTOR_AT)
clean_ref_to<-read.xlsx("./Cleaned Data/Variable Cleaning/Atacama accepting center cleaned.xlsx",cols =
c(1,3,5,6))
clean_E_OTOR_AT<-read.xlsx("./Cleaned Data/Variable Cleaning/E_OTOR_AT cleaned.xlsx",cols = c(1,3,5,6))
clean_areaofcare<-read.xlsx("./Cleaned Data/Variable Cleaning/type of service mapping for Rodrigo
v6.xlsx",cols = c(1,2,4))
colnames(clean_E_OTOR_AT)<-c("E_OTOR_AT","E_OTOR_AT_level","E_OTOR_AT_type","E_OTOR_AT_location")
colnames(clean_ref_to)<-c("ref_to","ref_to_level","ref_to_type","ref_to_location")
colnames(clean_areaofcare)<-c("location","areaofcare","specialty")
clean_E_OTOR_AT$E_OTOR_AT<-as.factor(clean_E_OTOR_AT$E_OTOR_AT)
clean_ref_to$ref_to<-as.factor(clean_ref_to$ref_to)
clean_areaofcare<-clean_areaofcare[clean_areaofcare$location=="Atacama",]
clean_areaofcare$areaofcare<-as.factor(clean_areaofcare$areaofcare)
clean_areaofcare$specialty<-as.factor(clean_areaofcare$specialty)
cleanv2<-left_join(cleanv1,clean_E_OTOR_AT,by = "E_OTOR_AT")
cleanv2<-left_join(cleanv2,clean_ref_to,by = "ref_to")
```

```
cleanv2<-left_join(cleanv2,clean_areaofcare,by = "areaofcare")
## Check if there is any case miss cleaning
sum(is.na(cleanv2$E_OTOR_AT_type))
sum(is.na(cleanv2$ref_to_type))
sum(is.na(cleanv2$specialty))
str(cleanv2)
cleanv2<-cleanv2 %>% mutate(id_orig = as.character(id_orig),
                            healthdept = as.factor(healthdept),
                            accepting_location = as.factor(ref_to_location),
                            referring_location = as.factor(E_OTOR_AT_location),
                            accepting_type = as.factor(ref_to_level),
                            referring_type = as.factor(E_OTOR_AT_level),
                            referring_subtype = as.factor(E_OTOR_AT_type),
                            accepting_subtype = as.factor(ref_to_type),
                            year_entry = as.factor(year_entry),
                            month_entry = as.factor(month_entry),
                            health_service = as.factor(health_service),
                            death = ifelse(is.na(deathdate),0,1),
                            specialty = as.factor(specialty)) %>% filter(!(specialty=="EXCLUDE"))
str(cleanv2)
levels(cleanv2$referring_subtype)<-c("rural primary center","urban primary center","health
service","tertiary high complexity",
                                     "tertiary low complexity","tertiary intermediate complexity")
levels(cleanv2$accepting_subtype)<-c("rural primary center","urban primary center","tertiary high
complexity","tertiary low complexity","tertiary intermediate complexity")
levels(cleanv2$referring_type)<-c("No Matched","primary","tertiary")
levels(cleanv2$accepting_type)<-c("primary","tertiary")
levels(cleanv2$healthdept)<-"Atacama"
cleanv2<-cleanv2[which(cleanv2$accepting_subtype!="0"),]
cleanv2<-cleanv2[which(cleanv2$referring_subtype!="0"),]
cleanv2<-cleanv2[which(cleanv2$referring_type!="No Matched"),]
summary(cleanv2)
write.csv(cleanv2,"./Raw Data/dataset during cleanning/Atacama/cleanv2.csv",row.names = F)
# select what we need for table
cleanv2<-read.csv("./Raw Data/dataset during cleanning/Atacama/cleanv2.csv")
summary(cleanv2)
cleanv2$deathtime<-ifelse(is.na(cleanv2$deathdate),as.Date("2017-12-31")-
as.Date(cleanv2$entrydate),as.Date(cleanv2$deathdate)-as.Date(cleanv2$entrydate))
cleanv3<- cleanv2 %>%
select(c(id_orig,Age,age,sex,WT,WT2,wtime,rural_urban,year_entry,specialty,death,exit_reason,healthdept,in
surance,referring_type,referring_subtype,referring_location,
accepting_type,accepting_subtype,accepting_location,deathtime,ref_to,entrydate,exitdate,deathdate))
str(cleanv3)
colnames(cleanv3)<-
c("id_orig","age_category","age_numeric","sex","waitingtime_category","waitingtime_category2","waitingtime
_numeric","rural_urban","year_entry","specialty",
"death","exit_reason","healthdept","insurance","referring_type","referring_subtype","referring_location","
accepting_type","accepting_subtype","accepting_locaton","deathtime","accepting",
                    "entrydate","exitdate","deathdate")
Atacama<-cleanv3[which(cleanv3$deathtime>=0),]
str(Atacama)
write.csv(Atacama,"./Cleaned Data/Atacama Cleaned.csv",row.names = F)
```

```
#DATA CLEANING VALPARAISO####
#############################################################################################################
#1. LOAD R PACKAGES
# Init
library(dplyr)
library(openxlsx)
library(lubridate)
library(stringr)
setwd("UNIQUE TO THE USER RUNNING THIS PROCEDURE")
#############################################################################################################
#2. LOAD DATASETS
d1<-read.xlsx("./Raw Data/NO-GES_NewSpecialty_Valpo-SanAntonio_1.xlsx",sheet = 2,startRow = 2,colNames =
F)
d2<-read.xlsx("./Raw Data/NO-GES_NewSpecialty_Valpo-SanAntonio_2.xlsx",sheet = 2,startRow = 2,colNames =
F)
d3<-read.xlsx("./Raw Data/NO-GES_NewSpecialty_Valpo-SanAntonio_3.xlsx",sheet = 2,startRow = 2,colNames =
F)
d4<-read.xlsx("./Raw Data/NO-GES_NewSpecialty_Valpo-SanAntonio_4.xlsx",sheet = 2,startRow = 2,colNames =
F)
d5<-read.xlsx("./Raw Data/NO-GES_NewSpecialty_Valpo-SanAntonio_5.xlsx",sheet = 2,startRow = 2,colNames =
F)
# Merge
dirty<-rbind(d1,d2,d3,d4,d5)
write.csv(dirty,file = "./Raw Data/dataset during cleanning/Valparaiso/merged dirty data.csv",row.names =
F)
#############################################################################################################
# 3. DAYA CLEANING
dirty<-read.csv("./Raw Data/dataset during cleanning/Valparaiso/merged dirty data.csv")
colnames(dirty)<-
c("id_orig","healthdept","birthdate","sex","insurance","health_service","ref_from","borough",

"rural_urban","ref_to","areaofcare","entrydate","year_entry","month_entry","exitdate","exit_reason",
                   "program","apptdate","deathdate","admini_alert","admini_alert_desc")
summary(dirty)
dirty1<-dirty %>% mutate(id_orig = as.character(id_orig),
                         healthdept = as.factor(healthdept),
                         birthdate = as.Date(birthdate,"%d/%m/%Y"),
                         sex =
as.factor(ifelse(sex==1,"Male",ifelse(sex==2,"Female",ifelse(sex==3,"Indetermined",NA)))),
                         insurance=
as.factor(ifelse(insurance==1,"public",ifelse(insurance==2,"private",ifelse(insurance==3,"Independent",NA)
))),
                         health_service = as.factor(health_service),
                         borough = as.factor(borough),
                         ref_from = as.factor(ref_from),
                         ref_to = as.factor(ref_to),
                         exit_reason = as.factor(exit_reason),
                         rural_urban =
ifelse(rural_urban==1,"urban",ifelse(rural_urban==2,"rural","other")),
                         entrydate = as.Date(entrydate,"%d/%m/%Y"),
                         year_entry = year(entrydate),
                         month_entry = month(entrydate),
                         exitdate = as.Date(exitdate,"%d/%m/%Y"),
                         program = as.factor(ifelse(program=="",NA,program)),
                         apptdate = as.Date(apptdate,"%d/%m/%Y"),
                         deathdate = as.Date(deathdate,"%d/%m/%Y"),
                         wtime = as.numeric(exitdate - entrydate),
                         age = as.numeric(floor((entrydate-birthdate)/365)),
                         admini_alert_desc=as.factor(admini_alert_desc),
                         WT = as.factor(ifelse(wtime<31,"0-30",
                                               ifelse(wtime<61,"31-60",ifelse(wtime<181,"61-180",
ifelse(wtime>180,"181+",NA))))),
                         WT2 = as.factor(ifelse(wtime<90,"<90",ifelse(wtime<121,"90-120",">120"))),
                         Age = as.factor(ifelse(age<15,"<15",
                                                ifelse(age<46,"15-45",ifelse(age<56,"46-
55",ifelse(age<66,"56-65",
```

```
ifelse(age<76,"66-75",ifelse(age<86,"76-85",ifelse(age>85,"85+","NA")))))))))
summary(dirty1)
## healthdept
levels(dirty1$healthdept)<-c("Iquique","Antofagasta","Atacama","Coquimbo","Vaparaiso-SanAntonio","Vina-
Quillota","Aconcagua","SSMN","SSMOC","SSMO",
                              "SSMS","SSMSO","OHiggins","Maule","Nuble","Concepcion","Valdivia")
## rural urban
dirty1$rural_urban[which(is.na(dirty1$rural_urban))]<-"other"
dirty1$rural_urban<-as.factor(dirty1$rural_urban)
## WT and Age
dirty1$WT<-ordered(dirty1$WT,levels=c("0-30","31-60","61-180","181+"))
dirty1$WT2<-ordered(dirty1$WT2,levels=c("<90","90-120",">120"))
dirty1$Age<-ordered(dirty1$Age,levels=c("<15","15-45","46-55","56-65","66-75","76-85","85+"))
## Medical Service
dirty1$areaofcare<-gsub("Ã-","i",dirty1$areaofcare)
dirty1$areaofcare<-gsub("Ã¡","a",dirty1$areaofcare)
dirty1$areaofcare<-gsub("Ã³","o",dirty1$areaofcare)
dirty1$areaofcare<-gsub("Ã©","e",dirty1$areaofcare)
dirty1$areaofcare<-gsub("Ã�","I",dirty1$areaofcare)
dirty1$areaofcare<-gsub("Ã�","O",dirty1$areaofcare)
dirty1$areaofcare<-str_to_title(dirty1$areaofcare)
dirty1$areaofcare<-as.factor(dirty1$areaofcare)
## Exit Reason
levels(dirty1$exit_reason)<-c("Ges","Completed Cases","Informed Procedure??","Medical
Revaluation","Completed Cases Externally??","Health Coverage Change",
                              "Patient Reject","Patient Spontaneous Recovery","Patient
Missed","Death","Duplicated Request??","Completed Case by Resolutivity",
                              "Completed Case by Telemedicine","Clinical Case Change","Contact Doesn't
Match??","Surgery Not Necessary","Transfer","No pertinencia??",
                              "Digitation Error")
## write data
cleanv1<-dirty1
str(cleanv1)
summary(cleanv1)
write.csv(cleanv1,file = "./Raw Data/dataset during cleanning/Valparaiso/cleanv1.csv",row.names = F)
## exclude data_exit missing
cleanv1<-cleanv1[which(!is.na(cleanv1$exitdate)),]
## exclude Indetermined Sex
cleanv1<-cleanv1[which(!(cleanv1$sex=="Indetermined")),]
## exclude negative age
cleanv1<-cleanv1[which(cleanv1$age>=0),]
cleanv1a<-cleanv1
summary(cleanv1a)
write.csv(cleanv1a,file = "./Raw Data/dataset during cleanning/Valparaiso/cleanv1a.csv",row.names = F)
# Clean    ref_from  ,   ref_to   ,   areaofcare
cleanv1a<-read.csv("./Raw Data/dataset during cleanning/Valparaiso/cleanv1a.csv")
clean_ref_to<-read.xlsx("./Cleaned Data/Variable Cleaning/Valparaiso accepting center cleaned.xlsx",cols =
c(1,3,4,5,6))
clean_ref_from<-read.xlsx("./Cleaned Data/Variable Cleaning/Valparaiso referring center cleaned.xlsx",cols
= c(1,3,4,5,6))
clean_areaofcare<-read.xlsx("./Cleaned Data/Variable Cleaning/type of service mapping for Rodrigo
v6.xlsx",cols = c(1,2,4))
colnames(clean_ref_from)<-
c("ref_from","ref_from_level","ref_from_name","ref_from_type","ref_from_location")
colnames(clean_ref_to)<-c("ref_to","ref_to_level","ref_to_name","ref_to_type","ref_to_location")
colnames(clean_areaofcare)<-c("location","areaofcare","specialty")
clean_ref_from$ref_from<-as.factor(clean_ref_from$ref_from)
clean_ref_to$ref_to<-as.factor(clean_ref_to$ref_to)
## select only Valparaiso
clean_areaofcare<-clean_areaofcare[clean_areaofcare$location=="Valparaiso",]
clean_areaofcare$areaofcare<-as.factor(clean_areaofcare$areaofcare)
clean_areaofcare$specialty<-as.factor(clean_areaofcare$specialty)
## join
cleanv2<-left_join(cleanv1a,clean_ref_from,by = "ref_from")
cleanv2<-left_join(cleanv2,clean_ref_to,by = "ref_to")
cleanv2<-left_join(cleanv2,clean_areaofcare,by = "areaofcare")
## check if there is any case miss cleaning
sum(is.na(cleanv2$ref_from_type))
```

```
sum(is.na(cleanv2$ref_to_type))
sum(is.na(cleanv2$specialty))
## clean new variable & exclude specialty=="EXCLUDE"
str(cleanv2)
cleanv2<-cleanv2 %>% mutate(id_orig = as.character(id_orig),
                            accepting_location = as.factor(ref_to_location),
                            referring_location = as.factor(ref_from_location),
                            accepting_type = as.factor(ref_to_level),
                            referring_type = as.factor(ref_from_level),
                            referring_subtype = as.factor(ref_from_type),
                            accepting_subtype = as.factor(ref_to_type),
                            program = as.factor(program),
                            year_entry = as.factor(year_entry),
                            month_entry = as.factor(month_entry),
                            borough = as.factor(borough),
                            health_service = as.factor(health_service),
                            death = as.factor(ifelse(is.na(deathdate),0,1)),
                            specialty = as.factor(specialty)) %>% filter(!(specialty=="EXCLUDE"))
str(cleanv2)
levels(cleanv2$referring_subtype)<-c("0","diagnostics center","reference medical center","rural primary
center","urban primary center","tertiary high complexity",
                                     "tertiary low complexity","tertiary intermediate complexity","rural
basic primary center","mobile unit")
levels(cleanv2$accepting_subtype)<-c("0","reference medical center","rural primary center","urban primary
center","tertiary high complexity","tertiary low complexity","tertiary intermediate complexity","rural
basic primary care")
levels(cleanv2$referring_type)<-c("primary","secondary","tertiary")
levels(cleanv2$accepting_type)<-c("primary","secondary","tertiary")
cleanv2<-cleanv2[which(cleanv2$accepting_subtype!="0"),]
cleanv2<-cleanv2[which(cleanv2$referring_subtype!="0"),]
summary(cleanv2)
write.csv(cleanv2,file = "./Raw Data/dataset during cleanning/Valparaiso/cleanv2.csv",row.names = F)
# Select what we need for tables
cleanv2<-read.csv(file = "./Raw Data/dataset during cleanning/Valparaiso/cleanv2.csv")
summary(cleanv2)
cleanv2$deathtime<-ifelse(is.na(cleanv2$deathdate),as.Date("2017-12-31")-
as.Date(cleanv2$entrydate),as.Date(cleanv2$deathdate)-as.Date(cleanv2$entrydate))
cleanv3<- cleanv2 %>%
select(c(id_orig,Age,age,sex,WT,WT2,wtime,rural_urban,year_entry,specialty,death,exit_reason,healthdept,in
surance,referring_type,referring_subtype,referring_location,

accepting_type,accepting_subtype,accepting_location,deathtime,ref_to,entrydate,exitdate,deathdate))
str(cleanv3)
colnames(cleanv3)<-
c("id_orig","age_category","age_numeric","sex","waitingtime_category","waitingtime_category2","waitingtime
_numeric","rural_urban","year_entry","specialty",

"death","exit_reason","healthdept","insurance","referring_type","referring_subtype","referring_location","
accepting_type","accepting_subtype","accepting_locaton","deathtime","accepting",
                     "entrydate","exitdate","deathdate")
Valparaiso<-cleanv3[which(cleanv3$deathtime>=0),]
write.csv(Valparaiso,file = "./Cleaned Data/Valparaiso Cleaned.csv",row.names = F)
```

```
#DATA CLEANING OSORNO####
#############################################################################################################
#1. LOAD R PACKAGES
library(openxlsx)
library(dplyr)
library(lubridate)
library(stringr)
#############################################################################################################
#2. LOAD DATASETS
setwd("UNIQUE TO THE USER RUNNING THIS PROCEDURE")
d1<-read.xlsx("Osorno_NSpecialty_2008-2014.xlsx")
d2<-read.xlsx("Osorno_NSpecialty_2015-2017.xlsx")
dirty<-rbind(d1,d2)
#############################################################################################################
3. DATA CLEANING
names<-
c("id_orig","birthdate","sex","insurance","health_service","referring","county","rural_urban","accepting",
"accepting_county","areaofcare","PRESTA_MIN","entrydate","exitdate",
"exit_reason","program","suspected_diag","confirmed_diag","apptdate","assistanceappt","deathdate","admini_
alert","admini_alert_decp")
colnames(dirty)<-names
write.csv(dirty,"./dataset during cleaning/merged dirty data.csv",row.names = F)
dirty<-read.csv("./dataset during cleaning/merged dirty data.csv")
str(dirty)
dirty1<-dirty %>% mutate(id_orig = as.character(id_orig),
                         birthdate = as.Date(birthdate,"%d/%m/%Y"),
                         sex =
as.factor(ifelse(sex==1,"Male",ifelse(sex==2,"Female",ifelse(sex==3,"Indetermined",ifelse(sex==4,"Unknown"
,NA))))),
                         insurance =
as.factor(ifelse(insurance==1,"public",ifelse(insurance==2,"private",ifelse(insurance==3,"Independent",NA)
))),
                         health_service = as.factor(health_service),
                         county = as.factor(county),
                         rural_urban =
ifelse(rural_urban==1,"urban",ifelse(rural_urban==2,"rural","other")),
                         entrydate = as.Date(entrydate,"%d/%m/%Y"),
                         exitdate = as.Date(exitdate,"%d/%m/%Y"),
                         exit_reason = as.factor(exit_reason),
                         program = as.factor(program),
                         apptdate = as.Date(apptdate,"%d/%m/%Y"),
                         deathdate = as.Date(deathdate,"%d/%m/%Y"),
                         death = ifelse(is.na(deathdate),0,1),
                         age_numeric = floor((entrydate-birthdate)/365),
                         age_category = as.factor(ifelse(age_numeric<15,"<15",
                                                         ifelse(age_numeric<46,"15-
45",ifelse(age_numeric<56,"46-55",ifelse(age_numeric<66,"56-65",

ifelse(age_numeric<76,"66-75",ifelse(age_numeric<86,"76-85",ifelse(age_numeric>85,"85+","NA")))))))),
                         waitingtime = exitdate-entrydate,
                         waitingtime_category = as.factor(ifelse(waitingtime<31,"0-30",
                                                                 ifelse(waitingtime<61,"31-
60",ifelse(waitingtime<181,"61-180",

ifelse(waitingtime>180,"181+",NA))))),
                         waitingtime_category2 =
as.factor(ifelse(waitingtime<90,"<90",ifelse(waitingtime<121,"90-120",">120"))))

summary(dirty1)

## rural urban
dirty1$rural_urban[which(is.na(dirty1$rural_urban))]<-"other"
dirty1$rural_urban<-as.factor(dirty1$rural_urban)
## WT and Age
dirty1$waitingtime_category<-ordered(dirty1$waitingtime_category,levels=c("0-30","31-60","61-180","181+"))
dirty1$waitingtime_category2<-ordered(dirty1$waitingtime_category2,levels=c("<90","90-120",">120"))
dirty1$age_category<-ordered(dirty1$age_category,levels=c("<15","15-45","46-55","56-65","66-75","76-
85","85+"))
```

```
## areaofcare
dirty1$areaofcare<-gsub("Ã-","i",dirty1$areaofcare)
dirty1$areaofcare<-gsub("Ã¡","a",dirty1$areaofcare)
dirty1$areaofcare<-gsub("Ã³","o",dirty1$areaofcare)
dirty1$areaofcare<-gsub("Ã©","e",dirty1$areaofcare)
dirty1$areaofcare<-gsub("Ã⍰","I",dirty1$areaofcare)
dirty1$areaofcare<-gsub("Ã⍰","O",dirty1$areaofcare)
dirty1$areaofcare<-str_to_title(dirty1$areaofcare)
dirty1$areaofcare<-as.factor(dirty1$areaofcare)
## Exit Reason
levels(dirty1$exit_reason)<-c("Ges","Completed Cases","Informed Procedure??","Medical
Revaluation","Completed Cases Externally??","Health Coverage Change",
                              "Patient Reject","Patient Spontaneous Recovery","Patient
Missed","Death","Duplicated Request??","Completed Case by Resolutivity",
                              "Completed Case by Telemedicine","Clinical Case Change","Contact Doesn't
Match??","Surgery Not Necessary","Transfer","No pertinencia??",
                              "Digitation Error")
summary(dirty1)
cleanv1<-dirty1
## exclude missing exitdate
cleanv1<-cleanv1[which(!is.na(cleanv1$exitdate)),] # NO
## exclude Indetermined Sex
cleanv1<-cleanv1[which(!(cleanv1$sex%in%c("Indetermined","Unknown"))),]
## exclude negative age
cleanv1<-cleanv1[which(cleanv1$age_numeric>=0),]
write.csv(cleanv1,"./dataset during cleaning/cleanv1.csv",row.names = F)
#####################
cleanv1<-read.csv("./dataset during cleaning/cleanv1.csv")
clean_ref_to<-read.xlsx("accepting center cleaned.xlsx",cols=c(1,3,4,5,6))
clean_ref_from<-read.xlsx("referring center cleaned.xlsx",cols = c(1,3,4,5,6))
clean_areaofcare<-read.xlsx("type of service mapping for Rodrigo v6.xlsx",cols = c(1,2,4))
colnames(clean_ref_from)<-
c("referring","ref_from_level","ref_from_name","ref_from_type","ref_from_location")
colnames(clean_ref_to)<-c("accepting","ref_to_level","ref_to_name","ref_to_type","ref_to_location")
colnames(clean_areaofcare)<-c("location","areaofcare","specialty")
clean_ref_from$referring<-as.factor(clean_ref_from$referring)
clean_ref_to$accepting<-as.factor(clean_ref_to$accepting)
clean_areaofcare<-clean_areaofcare[which(clean_areaofcare$location=="Osorno"),]
clean_areaofcare$areaofcare<-as.character(clean_areaofcare$areaofcare)
clean_areaofcare$specialty<-as.factor(clean_areaofcare$specialty)
str(cleanv1)
## join
cleanv2<-left_join(cleanv1,clean_ref_from,by = "referring")
cleanv2<-left_join(cleanv2,clean_ref_to,by = "accepting")
write.xlsx(unique(cleanv2$areaofcare),"need clean.xlsx",row.names=F)
cleanv2$areaofcare<-as.character(cleanv2$areaofcare)
cleanv2<-left_join(cleanv2,clean_areaofcare,by = "areaofcare")
## check if there is any case miss cleaning
sum(is.na(cleanv2$ref_from_type))
sum(is.na(cleanv2$ref_to_type))
sum(is.na(cleanv2$specialty))
## clean new variable & exclude specialty=="EXCLUDE"
str(cleanv2)
cleanv2<-cleanv2 %>% mutate(id_orig = as.character(id_orig),
                            accepting_location = as.factor(ref_to_location),
                            referring_location = as.factor(ref_from_location),
                            accepting_type = as.factor(ref_to_level),
                            referring_type = as.factor(ref_from_level),
                            referring_subtype = as.factor(ref_from_type),
                            accepting_subtype = as.factor(ref_to_type),
                            program = as.factor(program),
                            year_entry = as.factor(year(entrydate)),
                            county = as.factor(county),
                            health_service = as.factor(health_service),
                            death = as.factor(ifelse(is.na(deathdate),0,1)),
                            specialty = as.factor(specialty)) %>% filter(!(specialty=="EXCLUDE"))
str(cleanv2)
levels(cleanv2$referring_subtype)<-c("0","rural primary center","urban primary center","health
service","tertiary high complexity",
```

```
                                     "tertiary low complexity","health service","rural basic primary
center")

levels(cleanv2$accepting_subtype)<-c("0","rural primary center","urban primary center","tertiary high
complexity","tertiary low complexity","rural basic primary care")
levels(cleanv2$referring_type)<-c("No Matched","primary","tertiary")
levels(cleanv2$accepting_type)<-c("primary","tertiary")

cleanv2<-cleanv2[which(cleanv2$accepting_subtype!="0"),]
cleanv2<-cleanv2[which(cleanv2$referring_subtype!="0"),]
cleanv2<-cleanv2[which(cleanv2$referring_type!="No Matched"),]
summary(cleanv2)
cleanv2$healthdept<-"Osorno"
write.csv(cleanv2,file = "cleanv2.csv",row.names = F)
####################
cleanv2<-read.csv("cleanv2.csv")
str(cleanv2)
cleanv2$entrydate<-as.Date(cleanv2$entrydate,"%Y-%m-%d")
sum(cleanv2$entrydate<as.Date("01/01/2008","%d/%m/%Y"))
cleanv2<-cleanv2[which(cleanv2$entrydate>=as.Date("01/01/2008","%d/%m/%Y")),]
cleanv2$deathtime<-ifelse(is.na(cleanv2$deathdate),as.Date("2017-12-31")-
as.Date(cleanv2$entrydate),as.Date(cleanv2$deathdate)-as.Date(cleanv2$entrydate))
cleanv3<- cleanv2 %>%
select(c(id_orig,age_category,age_numeric,sex,waitingtime_category,waitingtime_category2,waitingtime,rural
_urban,year_entry,specialty,death,exit_reason,healthdept,insurance,referring_type,referring_subtype,referr
ing_location,

accepting_type,accepting_subtype,accepting_location,deathtime,accepting,entrydate,exitdate,deathdate))
colnames(cleanv3)<-
c("id_orig","age_category","age_numeric","sex","waitingtime_category","waitingtime_category2","waitingtime
_numeric","rural_urban","year_entry","specialty",

"death","exit_reason","healthdept","insurance","referring_type","referring_subtype","referring_location","
accepting_type","accepting_subtype","accepting_locaton","deathtime","accepting",
                     "entrydate","exitdate","deathdate")
summary(cleanv3)
cleanv3$healthdept<-"Osorno"
Osorno<-cleanv3[which(cleanv3$deathtime>=0),]
str(Osorno)
write.csv(Osorno,"C:/Users/hzhan/Dropbox/TRANSPARENCY DATA/Data Processing/Cleaned Data/Osorno
Cleaned.csv",row.names = F)
```
