# Supplementary material for: Prolonged wait time is associated with increased mortality for Chilean waiting list patients with non-prioritized conditions
Source: BMC Public Health. 2019 Feb 26;19:233. doi: 10.1186/s12889-019-6526-6 (PMC6390314; doi:10.1186/s12889-019-6526-6)
Supplement: Supplementary file 3 — Data Analysis Procedures. (PDF 163 kb) [file 12889_2019_6526_MOESM3_ESM.pdf]

Prolonged Wait Time is Associated with Increased Mortality for Chilean Waiting List Patients with Non-Prioritized Conditions

Diego A. Martinez, PhD,[1] Haoxiang Zhang, MSc,[1] Magdalena Bastias, PhD,[2] Felipe Feijoo,[3] PhD, Jeremiah Hinson, MD, PhD,[1] Rodrigo Martinez, MD,[2] Jocelyn Dunstan,[2] PhD, Scott Levin, PhD,[1] Diana Prieto, PhD[1]

[1]Johns Hopkins University
[2]University of Chile
[3]Pontifical Catholic University of Valparaíso

Additional File 3: Data Analysis Procedures

```
####0. SCRIPT PARAMETERS####
#set working directory
setwd("C:/XXX")
getwd()
#specify the location of the cleaned datasets
link1<-"C:/XXX/Atacama Cleaned.csv"
link2<-"C:/XXX/Valparaiso Cleaned.csv"
link3<-"C:/XXX/Osorno Cleaned.csv"
link1<-read.csv(file.choose(), header = TRUE)#"Atacama Cleaned.csv"
link2<-read.csv(file.choose(), header = TRUE)#"Valparaiso Cleaned.csv"
link3<-read.csv(file.choose(), header = TRUE)#"Osorno Cleaned.csv"
#####################################################################################################################################
##########################################################################################
```

```
####1. LOAD PACKAGES####
library(openxlsx)
library(survival)
library(dplyr)
library(ggplot2)
library(survminer)
library(rms)
library(coxme)
library(Rmisc)
library(ggfortify)
#####################################################################################################################################
##########################################################################################
```

```
####2. LOAD CLEANED DATASETS####
Atadata<-link1
Atadata<-Atadata %>%
  mutate(deathtime = as.numeric(deathtime),
         age_category = as.factor(ifelse(age_category=="<15",ifelse(age_numeric<=3,"0-
3",ifelse(age_numeric<=7,"4-7",ifelse(age_numeric<=11,"8-11","12-14"))),as.character(age_category))),
         death2y = ifelse(death==0,0,ifelse(deathtime<=365*2,1,0)),
         time_to_event2y = ifelse(death2y==1,deathtime,2*365),
         death2.5y = ifelse(death==0,0,ifelse(deathtime<=floor(365*2.5),1,0)),
         time_to_event2.5y = ifelse(death2y==1,deathtime,floor(365*2.5)),
         death3y = ifelse(death==0,0,ifelse(deathtime<=365*3,1,0)),
         time_to_event3y = ifelse(death2y==1,deathtime,3*365)
         )
Atadata$age_category<-ordered(Atadata$age_category,levels=c("0-3","4-7","8-11","12-14","15-45","46-
55","56-65","66-75","76-85","85+"))
str(Atadata)
Valdata<-link2
Valdata<-Valdata %>%
  mutate(deathtime = as.numeric(deathtime),
         age_category = as.factor(ifelse(age_category=="<15",ifelse(age_numeric<=3,"0-
3",ifelse(age_numeric<=7,"4-7",ifelse(age_numeric<=11,"8-11","12-14"))),as.character(age_category))),
         death2y = ifelse(death==0,0,ifelse(deathtime<=365*2,1,0)),
         time_to_event2y = ifelse(death2y==1,deathtime,2*365),
         death2.5y = ifelse(death==0,0,ifelse(deathtime<=floor(365*2.5),1,0)),
         time_to_event2.5y = ifelse(death2y==1,deathtime,floor(365*2.5)),
         death3y = ifelse(death==0,0,ifelse(deathtime<=365*3,1,0)),
         time_to_event3y = ifelse(death2y==1,deathtime,3*365)
         )
Valdata$age_category<-ordered(Valdata$age_category,levels=c("0-3","4-7","8-11","12-14","15-45","46-
55","56-65","66-75","76-85","85+"))
str(Valdata)
Osodata<-link3
Osodata<-Osodata %>%
  mutate(deathtime = as.numeric(deathtime),
         age_category = as.factor(ifelse(age_category=="<15",ifelse(age_numeric<=3,"0-
3",ifelse(age_numeric<=7,"4-7",ifelse(age_numeric<=11,"8-11","12-14"))),as.character(age_category))),
         death2y = ifelse(death==0,0,ifelse(deathtime<=365*2,1,0)),
         time_to_event2y = ifelse(death2y==1,deathtime,2*365),
         death2.5y = ifelse(death==0,0,ifelse(deathtime<=floor(365*2.5),1,0)),
         time_to_event2.5y = ifelse(death2y==1,deathtime,floor(365*2.5)),
         death3y = ifelse(death==0,0,ifelse(deathtime<=365*3,1,0)),
         time_to_event3y = ifelse(death2y==1,deathtime,3*365)
         )
Osodata$age_category<-ordered(Osodata$age_category,levels=c("0-3","4-7","8-11","12-14","15-45","46-
55","56-65","66-75","76-85","85+"))
#####################################################################################################
#####################################################################################

####3. DATASET PREPARATION####
all<-function(data){return(subset(data,data$age_numeric>=0))}
id<-Valdata[which(Valdata$healthdept=="Atacama"),]$id_orig
Atadata[which(Atadata$id_orig%in%id),]
Valdata[which(Valdata$healthdept=="Atacama"),]
Valdata2<-Valdata[-which(Valdata$healthdept=="Atacama"),]
```

```
data<-rbind(Atadata,Valdata2,Osodata)
summary(data$healthdept)
data$healthdept<-as.character(data$healthdept)
data$healthdept<-ifelse(data$healthdept%in%c("Vaparaiso-
SanAntonio","Atacama","Osorno"),data$healthdept,"Other")
data$healthdept<-as.factor(data$healthdept)
data_all<-data

##SET REFERENCE LEVELS AND GROUP MEDICAL SPECIALTIES WITH LOW N
#AGE
data_all$age_category<-factor(data_all$age_category, ordered=FALSE)
data_all$age_category<-relevel(data_all$age_category, ref="15-45")
#RESIDENCE
data_all$rural_urban<-relevel(data_all$rural_urban, ref="rural")
#INSURANCE
data_all$insurance<-ifelse(data_all$insurance=="public","public","private")
data_all$insurance<-factor(data_all$insurance)
data_all$insurance<-relevel(data_all$insurance, ref="public")
#SPECIALTY
#re-label those with low N and no contribution to mortality status death=yes, and group specialties with
low n
data_all$specialty2<-as.character(data_all$specialty)
data_all$specialty3<-ifelse(data_all$healthdept=="Atacama" & data_all$specialty2=="Anesthesiology",
"Other", # n=1, no death within 2 years
                            ifelse(data_all$healthdept=="Atacama" & data_all$specialty2=="Breast
Surgery","Other", # n=2, no death within 2 years
                                   ifelse(data_all$specialty2=="Occupational Health","Other", # n=28, no
death within 2 years
                                          ifelse(data_all$healthdept=="Atacama" &
data_all$specialty2=="Physical Medicine and Rehabilitation","Other",# n=1, no death within 2 years
                                                 ifelse(data_all$healthdept=="Atacama" &
data_all$specialty2=="Rheumatology","Other", # n=6, no death within 2 years
                                                        ifelse(data_all$healthdept=="Osorno" &
data_all$specialty2=="Infectious Disease","Other",# n=20, no death within 2 years
                                                               ifelse(data_all$specialty2=="Cardiac
Surgery","Cardiovascular Surgery", # n=32, registrants from Valparaiso

ifelse(data_all$specialty2=="Vascular Surgery", "Cardiovascular Surgery", # 2512 from Atacama, 7107 from
Osorno, 5849 from Valparaiso

ifelse(data_all$specialty2=="Geriatrics", "Other",#geriatrics N = 1

ifelse(data_all$specialty2=="Thorax Surgery","Adult Surgery",#thorax surgery n=210

ifelse(data_all$specialty2=="Abdominal Surgery","Adult Surgery",#abdominal surgery n=51

ifelse(data_all$specialty2=="Pediatric Surgery","Pediatrics",data_all$specialty2)))))))))))#pediatric
surgery n=167 from Valparaiso
#setting ref levels
data_all$specialty4<-as.factor(data_all$specialty3)
data_all$specialty4<-relevel(data_all$specialty4, ref="Internal Medicine")
#REFERRING LOCATION
data_all$referring_location<-relevel(data_all$referring_location, ref="Urban")
#ACCEPTING
data_all$accepting_type<-relevel(data_all$accepting_type, ref="tertiary")
#REFERRING SUBTYPE
data_all$referring_subtype2<-as.character(data_all$referring_subtype)
data_all$referring_subtype2<-ifelse(data_all$referring_subtype2=="mobile unit", "urban primary center",
data_all$referring_subtype2)
data_all$referring_subtype2<-as.factor(data_all$referring_subtype2)
data_all$referring_subtype2<-relevel(data_all$referring_subtype2, ref="tertiary high complexity")
#ACCEPTING SUBTYPE
data_all$accepting_subtype<-relevel(data_all$accepting_subtype, ref="tertiary high complexity")
#YEAR OF WAITLIST ENTRY
data_all$year_entry2<-as.factor(data_all$year_entry)
data_all$year_entry2<-relevel(data_all$year_entry2, ref="2008")
#ACCEPTING LEVEL OF CARE
data_all$accepting_type<-as.factor(data_all$accepting_type)
data_all$accepting_type<-relevel(data_all$accepting_type, ref="primary")
```

```
#REFERRING LEVEL OF CARE
data_all$referring_type<-as.factor(data_all$referring_type)
data_all$referring_type<-relevel(data_all$referring_type, ref="primary")

##inclusion/exclusion criteria based on "health department"
#we excluded 4740 out of 1044528 (0.45%) registrants listed in health departments other than atacama,
valparaiso, and osorno
data_all<-data_all[which(data_all$healthdept!="Other"),]

##inclusion/exclusion criteria based on "reason of exit from the waitlist"
#include: Death, Completed Case by Resolutivity, Completed Case by Telemedicine, Completed Cases,
Completed Cases Externally??, Health Coverage Change, Informed Procedure??, Medical Revaluation, Patient
Missed,  Patient Reject, Patient Spontaneous Recovery, Surgery Not Necessary, Clinical Case Change
#exclude: Contact Doesn't Match??, Digitation Error, Duplicated Request??, Ges, No pertinencia??
#we excluded 26222 out of 987497 (2.66%) registrants because follow up was not possible, i.e., "Contact
Doesn't Match" (N=25,572), "Digitation Error" (N=650)
#and we also excluded 15643 out of 987497 (1.58%) registrants because they were GES, i.e., "GES"
(N=13782), "No Pertinencia" (N=1861)
data_all<-data_all[which(data_all$exit_reason!="Contact Doesn't Match??" &
                           data_all$exit_reason!="Digitation Error" &
                           data_all$exit_reason!="Duplicated Request??" &
                           data_all$exit_reason!="Ges" &
                           data_all$exit_reason!="No pertinencia??"),]
##subsetting based on three outcomes of interest: 2-, 2.5- and 3-year mortality
myvars2 <-
c("age_category","sex","rural_urban","specialty4","healthdept","insurance","referring_type","accepting_typ
e","time_to_event2y","death2y","waitingtime_numeric", "accepting")
myvars2.5 <-
c("age_category","sex","rural_urban","specialty4","healthdept","insurance","referring_type","accepting_typ
e","time_to_event2.5y","death2.5y","waitingtime_numeric", "accepting")
myvars3 <-
c("age_category","sex","rural_urban","specialty4","healthdept","insurance","referring_type","accepting_typ
e","time_to_event3y","death3y","waitingtime_numeric", "accepting")
data_all2y<-data_all[myvars2]
data_all2.5y<-data_all[myvars2.5]
data_all3y<-data_all[myvars3]
#############################################################################################################
##########################################################################################
```

```
####3. STATISTICAL ANALYSIS: INDIVIDUAL-LEVEL SURVIVAL MODELING####
##SURVIVAL OVERALL####
all_RandomEffects2<-coxme(Surv(time_to_event2y, death2y)~
                            age_category+
                            sex+
                            rural_urban+
                            specialty4+
                            insurance+
                            referring_type+
                            (1|healthdept/accepting_type),
                          data=data_all2y)
```

```
##SURVIVAL OVERALL 2-, 2.5-, AND 3-YEAR MORTLITY####
all_RandomEffects2<-coxme(Surv(time_to_event2y, death2y)~
                            age_category+
                            sex+
                            rural_urban+
                            specialty4+
                            insurance+
                            referring_type+
                            (1|healthdept/accepting_type),
                          data=data_all2y)
all_RandomEffects2.5<-coxme(Surv(time_to_event2.5y, death2.5y)~
                              age_category+
                              sex+
                              rural_urban+
                              specialty4+
                              insurance+
                              referring_type+
                              (1|healthdept/accepting_type),
                            data=data_all2.5y)
all_RandomEffects3<-coxme(Surv(time_to_event3y, death3y)~
                            age_category+
                            sex+
                            rural_urban+
                            specialty4+
                            insurance+
                            referring_type+
                            (1|healthdept/accepting_type),
                          data=data_all3y)
##SURVIVAL ATACAMA####
all_RandomEffects2_atacama<-coxph(Surv(time_to_event2y, death2y)~
                            age_category+
                            sex+
                            rural_urban+
                            specialty4+
                            insurance+
                            referring_type+
                            accepting_type,
                          data=data_all2y[which(data_all2y$healthdept=="Atacama"),])
##SURVIVAL VALPARAISO####
all_RandomEffects2_valparaiso<-coxph(Surv(time_to_event2y, death2y)~
                            age_category+
                            sex+
                            rural_urban+
                            specialty4+
                            insurance+
                            referring_type+
                            accepting_type,
                          data=data_all2y[which(data_all2y$healthdept=="Vaparaiso-SanAntonio"),])
##SURVIVAL OSORNO####
all_RandomEffects2_osorno<-coxph(Surv(time_to_event2y, death2y)~
                            age_category+
                            sex+
                            rural_urban+
                            specialty4+
                            insurance+
                            referring_type+
                            accepting_type,
                          data=data_all2y[which(data_all2y$healthdept=="Osorno"),])
##SURVIVAL ONCOLOGY####
#ggsurvplot(survfit(Surv(time_to_event2y, death2y)~specialty4, data=data_all2y))
summary(as.factor(data_all2y$age_category))
oncology<-data_all2y[data_all2y$specialty4 == "Oncology",]
table(data_all2y$age_category,data_all2y$death2y)
oncology<-oncology[oncology$age_category == "85+"|
                     oncology$age_category =="76-85"|
                     oncology$age_category =="66-75"|
                     oncology$age_category =="56-65"|
                     oncology$age_category =="46-55"|
                     oncology$age_category =="15-45",]
```

```
summary(as.factor(data_all2y$age_category))
summary(as.factor(oncology$insurance))
ggsurvplot(survfit(Surv(time_to_event2y, death2y)~age_category, data=oncology))
fit_onc<-survfit(Surv(time_to_event2y, death2y)~1, data=oncology)
autoplot(fit_onc,censor.shape = '*', censor.size = 5)
summary(as.factor(oncology$rural_urban))
list(names(oncology))
str(oncology)
oncology$age_category<-as.factor(oncology$age_category)
oncology$sex<-as.factor(oncology$sex)
oncology$rural_urban<-as.factor(oncology$rural_urban)
oncology$healthdept<-as.factor(oncology$healthdept)
oncology$insurance<-as.factor(oncology$insurance)
oncology$referring_type<-as.factor(oncology$referring_type)
oncology$accepting_type<-as.factor(oncology$accepting_type)
summary(oncology)

oncology$referring_type[oncology$referring_type == "secondary"]<-"primary"
oncology$referring_type<-as.character(oncology$referring_type)
oncology$referring_type<-as.factor(oncology$referring_type)
table(oncology$sex,oncology$death2y)

#insurance, referring_type. accepting_type
oncology_ph<-coxph(Surv(time_to_event2y, death2y)~
                     age_category+
                     sex+
                     age_category:age_category+
                     rural_urban+
                     #insurance+
                     referring_type+
                     healthdept+
                     #accepting_type+
                     age_category:sex+
                     age_category:rural_urban+
                     age_category:healthdept+
                     age_category:referring_type+
                     #age_category:insurance+
                     age_category:rural_urban,
                   data=oncology)
summary(oncology_ph)
anova(oncology_ph)
fit<-survfit(oncology_ph,data=oncology)
obs<-survfit(Surv(time_to_event2y, death2y)~1, data=oncology)
KmPh <- list(Model = fit, KM = obs)
ggsurvplot(KmPh, data = oncology, combine = TRUE, # Combine curves
           risk.table = TRUE,                  # Add risk table
           conf.int = FALSE,                    # Add confidence interval
           conf.int.style = "step",            # CI style, use "step" or "ribbon"
           censor = FALSE,                     # Remove censor points
           tables.theme = theme_cleantable(),  # Clean risk table
           palette = "jco",ylim=c(0,1),xlim=c(0,1000))

resd<-resid(oncology_ph,"mart")
dev.off()
plot(resd)
hist(resd)
oncology2<-oncology
oncology2$resd<-resd
oncology2$ID <- seq.int(nrow(oncology2))
names(oncology2)
ggplot(oncology2, aes(x=ID, y=resd))+ geom_point(aes(colour = factor(age_category)))

#residual plots
par(mfrow=c(2,4))
plot(resd~as.factor(oncology$age_category))
plot(resd~as.factor(oncology$sex))
plot(resd~as.factor(oncology$rural_urban))
plot(resd~as.factor(oncology$specialty4))
plot(resd~as.factor(oncology$insurance))
```

```
plot(resd~as.factor(oncology$referring_type))
plot(resd~as.factor(oncology$healthdept))
plot(resd~as.factor(oncology$accepting_type))

par(mfrow=c(3,2))
interaction.plot(as.factor(oncology$age_category), as.factor(oncology$sex), resd)
interaction.plot(as.factor(oncology$age_category), as.factor(oncology$rural_urban), resd)
interaction.plot(as.factor(oncology$age_category), as.factor(oncology$specialty4), resd)
interaction.plot(as.factor(oncology$age_category), as.factor(oncology$insurance), resd)
interaction.plot(as.factor(oncology$age_category), as.factor(oncology$referring_type), resd)
interaction.plot(as.factor(oncology$age_category), as.factor(oncology$healthdept), resd)

summary(as.factor(oncology_2$death2y))
oncology2<-oncology[oncology$death2y=="0",]
oncology_2<-oncology[oncology$death2y=="1",]
oncology3<-sample_n(oncology2, 2236*1.5)
oncology4<-rbind(oncology3,oncology_2)
names(oncology4)
oncology4$age_category<-as.character(oncology4$age_category)
oncology4$age_category<-as.factor(oncology4$age_category)
oncology4$sex<-as.character(oncology4$sex)
oncology4$sex<-as.factor(oncology4$sex)
oncology4$rural_urban<-as.character(oncology4$rural_urban)
oncology4$rural_urban<-as.factor(oncology4$rural_urban)
oncology4$healthdept<-as.character(oncology4$healthdept)
oncology4$healthdept<-as.factor(oncology4$healthdept)
oncology4$referring_type<-as.character(oncology4$referring_type)
oncology4$referring_type<-as.factor(oncology4$referring_type)

oncology_ph<-coxph(Surv(time_to_event2y, death2y)~
                     age_category+
                     sex+
                     age_category:age_category+
                     rural_urban+
                     #insurance+
                     referring_type+
                     healthdept+
                     #accepting_type+
                     age_category:sex+
                     age_category:rural_urban+
                     age_category:healthdept+
                     age_category:referring_type+
                     #age_category:insurance+
                     age_category:rural_urban+
                     #sex:rural_urban+
                     sex:healthdept,
                   #sex:referring_type,
                   #healthdept:rural_urban,
                   #referring_type:rural_urban,
                   data=oncology4)
summary(oncology_ph)
anova(oncology_ph)
summary(oncology4$rural_urban)

fit<-survfit(oncology_ph,data=oncology4)
obs<-survfit(Surv(time_to_event2y, death2y)~1, data=oncology4)
KmPh <- list(Model = fit, KM = obs)
ggsurvplot(KmPh, data = oncology4, combine = TRUE, # Combine curves
           risk.table = TRUE,                  # Add risk table
           conf.int = FALSE,                    # Add confidence interval
           conf.int.style = "step",            # CI style, use "step" or "ribbon"
           censor = FALSE,                     # Remove censor points
           tables.theme = theme_cleantable(),  # Clean risk table
           palette = "jco",ylim=c(0,1),xlim=c(0,1000))

resd<-resid(oncology_ph,"mart")
oncology4$resd<-resd
par(mfrow=c(2,4))
plot(resd~as.factor(oncology4$age_category))
```

```
plot(resd~as.factor(oncology4$sex))
plot(resd~as.factor(oncology4$rural_urban))
plot(resd~as.factor(oncology4$specialty4))
plot(resd~as.factor(oncology4$insurance))
plot(resd~as.factor(oncology4$referring_type))
plot(resd~as.factor(oncology4$healthdept))
plot(resd~as.factor(oncology4$accepting_type))

par(mfrow=c(3,2))
interaction.plot(as.factor(oncology4$age_category), as.factor(oncology4$sex), resd)
interaction.plot(as.factor(oncology4$age_category), as.factor(oncology4$rural_urban), resd)
interaction.plot(as.factor(oncology4$age_category), as.factor(oncology4$specialty4), resd)
interaction.plot(as.factor(oncology4$age_category), as.factor(oncology4$insurance), resd)
interaction.plot(as.factor(oncology4$age_category), as.factor(oncology4$referring_type), resd)
interaction.plot(as.factor(oncology4$age_category), as.factor(oncology4$healthdept), resd)

par(mfrow=c(3,2))
interaction.plot(as.factor(oncology4$sex), as.factor(oncology4$rural_urban), resd)
interaction.plot(as.factor(oncology4$sex), as.factor(oncology4$specialty4), resd)
interaction.plot(as.factor(oncology4$sex), as.factor(oncology4$insurance), resd)
interaction.plot(as.factor(oncology4$sex), as.factor(oncology4$referring_type), resd)
interaction.plot(as.factor(oncology4$sex), as.factor(oncology4$healthdept), resd)

##figures oncology
tiff("Residuals versus age category for Oncology area.tiff", width = 6, height = 6, pointsize = 1/300, 
units = 'in', res = 300)
ggplot(oncology4, aes(x=age_category, y=resd, fill=age_category)) +
  geom_boxplot(alpha=.7)+
  theme_bw()+
  ylim(-3,3) +
  xlab("Age group") +
  ylab("Residuals")+
  theme(legend.title=element_blank()) + scale_color_grey()
dev.off()

tiff("Survival curve (Kaplan Meier) for Oncology.tiff", width = 6, height = 6, pointsize = 1/300, units = 
'in', res = 300)
obs<-survfit(Surv(time_to_event2y, death2y)~1, data=oncology4)
ggsurvplot(obs, data = oncology4, combine = TRUE, # Combine curves
           risk.table = FALSE,                  # Add risk table
           conf.int = TRUE,                    # Add confidence interval
           conf.int.style = "step",            # CI style, use "step" or "ribbon"
           censor = FALSE,                     # Remove censor points
           #tables.theme = theme_cleantable(),  # Clean risk table
           palette = "jco",ylim=c(0,1),xlim=c(0,800))
dev.off()

tiff("Survival curve (Kaplan Meier) and fitted Proportional Hazard model (Oncology).tiff", width = 6, 
height = 4, pointsize = 1/300, units = 'in', res = 300)
fit<-survfit(oncology_ph,data=oncology4)
obs<-survfit(Surv(time_to_event2y, death2y)~1, data=oncology4)
KmPh <- list(Model = fit, KM = obs)
ggsurvplot(KmPh, data = oncology4, combine = TRUE, # Combine curves
           risk.table = FALSE,                  # Add risk table
           conf.int = FALSE,                    # Add confidence interval
           conf.int.style = "step",            # CI style, use "step" or "ribbon"
           censor = FALSE,                     # Remove censor points
           tables.theme = theme_cleantable(),  # Clean risk table
           palette = "jco",ylim=c(0,1),xlim=c(0,800))
dev.off()

#KM CURVES FOR ONCOLOGY
tiff("Survival curve (Kaplan Meier) for sex group (Oncology).tiff", width = 6, height = 6, pointsize = 
1/300, units = 'in', res = 300)
obs<-survfit(Surv(time_to_event2y, death2y)~sex, data=oncology4)
sex_plot<-ggsurvplot(obs, data = oncology4, combine = TRUE, # Combine curves
                     risk.table = FALSE,                  # Add risk table
                     conf.int = TRUE,                    # Add confidence interval
                     conf.int.style = "step",            # CI style, use "step" or "ribbon"
```

```
                     censor = FALSE,                      # Remove censor points
                     #tables.theme = theme_cleantable(),  # Clean risk table
                     palette = "jco",ylim=c(0,1),xlim=c(0,800),legend.title = "Covariate: Sex 
Levels:",
                     legend.labs = c("Female", "Male"), font.legend = c(14, "plain", "black"))
dev.off()

tiff("Survival curve (Kaplan Meier) for age group (Oncology).tiff", width = 6, height = 6, pointsize = 
1/300, units = 'in', res = 300)
summary(oncology4$age_category)
obs<-survfit(Surv(time_to_event2y, death2y)~age_category, data=oncology4)
age_plot<-ggsurvplot(obs, data = oncology4, combine = TRUE, # Combine curves
                     risk.table = FALSE,                  # Add risk table
                     conf.int = FALSE,                    # Add confidence interval
                     conf.int.style = "step",            # CI style, use "step" or "ribbon"
                     censor = FALSE,                     # Remove censor points
                     #tables.theme = theme_cleantable(),  # Clean risk table
                     palette = "jco",ylim=c(0,1),xlim=c(0,800),legend.title = "Covariate: Age 
Levels:",
                     legend.labs = c("14-45", "46-55","56-65","66-75","76-85", "85+"), font.legend = c(14, 
"plain", "black"))
dev.off()

summary(oncology4$rural_urban)
tiff("Survival curve (Kaplan Meier) for area of recidence (Oncology).tiff", width = 6, height = 6, 
pointsize = 1/300, units = 'in', res = 300)
obs<-survfit(Surv(time_to_event2y, death2y)~rural_urban, data=oncology4)
Residence_plot<-ggsurvplot(obs, data = oncology4, combine = TRUE, # Combine curves
                           risk.table = FALSE,                  # Add risk table
                           conf.int = FALSE,                    # Add confidence interval
                           conf.int.style = "step",            # CI style, use "step" or "ribbon"
                           censor = FALSE,                     # Remove censor points
                           #tables.theme = theme_cleantable(),  # Clean risk table
                           palette = "jco",ylim=c(0,1),xlim=c(0,800),legend.title = "Covariate: Residence 
Levels:",
                           legend.labs = c("Other", "Rural","Urban"), font.legend = c(14, "plain", 
"black"))
dev.off()


summary(oncology4$healthdept)
tiff("Survival curve (Kaplan Meier) for Health service (Oncology).tiff", width = 8, height = 6, pointsize 
= 1/300, units = 'in', res = 300)
obs<-survfit(Surv(time_to_event2y, death2y)~healthdept, data=oncology4)
healthdept_Plot<-ggsurvplot(obs, data = oncology4, combine = TRUE, # Combine curves
                            risk.table = FALSE,                  # Add risk table
                            conf.int = FALSE,                    # Add confidence interval
                            conf.int.style = "step",            # CI style, use "step" or "ribbon"
                            censor = FALSE,                     # Remove censor points
                            #tables.theme = theme_cleantable(),  # Clean risk table
                            palette = "jco",ylim=c(0,1),xlim=c(0,800),legend.title = "Covariate: Health 
Service        Levels:",
                            legend.labs = c("Atacama", "Osorno","Valparaiso-San Antonio"), font.legend = 
c(14, "plain", "black"))+ guides(colour = guide_legend(nrow = 2))

dev.off()


require(gridExtra)
require("survminer")
splots <- list()
splots[[1]] <- sex_plot
splots[[2]] <- healthdept_Plot
splots[[3]] <- age_plot
splots[[4]] <- Residence_plot

tiff("Survival curves (Kaplan Meier) for covariates in Oncology specialty Cox proportional model.tiff", 
width = 14, height = 14, pointsize = 1/300, units = 'in', res = 300)
arrange_ggsurvplots(splots, print = TRUE,
```

```
                    ncol = 2, nrow = 2, risk.table.height = 0.4)
dev.off()

oncology_ph$xlevels

#KM CURVES ALL DATA
tiff("Survival curve (Kaplan Meier) for sex group (Oncology).tiff", width = 6, height = 6, pointsize =
1/300, units = 'in', res = 300)
obs<-survfit(Surv(time_to_event2y, death2y)~sex, data=data_all2y)
sex_plot<-ggsurvplot(obs, data = oncology4, combine = TRUE, # Combine curves
                     risk.table = FALSE,                  # Add risk table
                     conf.int = TRUE,                    # Add confidence interval
                     conf.int.style = "step",            # CI style, use "step" or "ribbon"
                     censor = FALSE,                     # Remove censor points
                     #tables.theme = theme_cleantable(),  # Clean risk table
                     palette = "jco",ylim=c(0.75,1),xlim=c(0,800),legend.title = "Covariate: Sex
Levels:",
                     legend.labs = c("Female", "Male"), font.legend = c(14, "plain", "black"))
dev.off()

tiff("Survival curve (Kaplan Meier) for age group (Oncology).tiff", width = 6, height = 6, pointsize =
1/300, units = 'in', res = 300)
summary(data_all2y$age_category)
obs<-survfit(Surv(time_to_event2y, death2y)~age_category, data=data_all2y)
age_plot<-ggsurvplot(obs, data = oncology4, combine = TRUE, # Combine curves
                     risk.table = FALSE,                  # Add risk table
                     conf.int = FALSE,                    # Add confidence interval
                     conf.int.style = "step",            # CI style, use "step" or "ribbon"
                     censor = FALSE,                     # Remove censor points
                     #tables.theme = theme_cleantable(),  # Clean risk table
                     palette = "jco",ylim=c(0.5,1),xlim=c(0,800),legend.title = "Covariate: Age
Levels:",
                     legend.labs = c("14-45","0-3","4-7","8-11","12-14","46-55","56-65","66-75","76-85",
"85+"), font.legend = c(14, "plain", "black"))
dev.off()

summary(data_all2y$rural_urban)
tiff("Survival curve (Kaplan Meier) for area of recidence (Oncology).tiff", width = 6, height = 6,
pointsize = 1/300, units = 'in', res = 300)
obs<-survfit(Surv(time_to_event2y, death2y)~rural_urban, data=data_all2y)
Residence_plot<-ggsurvplot(obs, data = oncology4, combine = TRUE, # Combine curves
                           risk.table = FALSE,                  # Add risk table
                           conf.int = FALSE,                    # Add confidence interval
                           conf.int.style = "step",            # CI style, use "step" or "ribbon"
                           censor = FALSE,                     # Remove censor points
                           #tables.theme = theme_cleantable(),  # Clean risk table
                           palette = "jco",ylim=c(0.75,1),xlim=c(0,800),legend.title = "Covariate:
Residence        Levels:",
                           legend.labs = c("Rural", "Other","Urban"), font.legend = c(14, "plain",
"black"))
dev.off()

summary(data_all2y$healthdept)
tiff("Survival curve (Kaplan Meier) for Health service (Oncology).tiff", width = 8, height = 6, pointsize
= 1/300, units = 'in', res = 300)
obs<-survfit(Surv(time_to_event2y, death2y)~healthdept, data=data_all2y)
healthdept_Plot<-ggsurvplot(obs, data = oncology4, combine = TRUE, # Combine curves
                            risk.table = FALSE,                  # Add risk table
                            conf.int = FALSE,                    # Add confidence interval
                            conf.int.style = "step",            # CI style, use "step" or "ribbon"
                            censor = FALSE,                     # Remove censor points
                            #tables.theme = theme_cleantable(),  # Clean risk table
                            palette = "jco",ylim=c(0.75,1),xlim=c(0,800),legend.title = "Covariate: Health
Service        Levels:",
                            legend.labs = c("Atacama", "Osorno","Valparaiso-San Antonio"), font.legend =
c(14, "plain", "black"))+ guides(colour = guide_legend(nrow = 2))
dev.off()

require(gridExtra)
```

```
require("survminer")
splots <- list()
splots[[1]] <- sex_plot
splots[[2]] <- healthdept_Plot
splots[[3]] <- age_plot
splots[[4]] <- Residence_plot

tiff("Survival curves (Kaplan Meier) for covariates Cox proportional model.tiff", width = 14, height = 14,
pointsize = 1/300, units = 'in', res = 300)
arrange_ggsurvplots(splots, print = TRUE,
                    ncol = 2, nrow = 2, risk.table.height = 0.4)
dev.off()

fit<-survfit(all_RandomEffects2,data=data_all2y)
obs<-survfit(Surv(time_to_event2y, death2y)~1, data=data_all2y)
KmPh <- list(Model = fit, KM = obs)
ggsurvplot(KmPh, data = data_all2y, combine = TRUE, # Combine curves
           risk.table = TRUE,                  # Add risk table
           conf.int = TRUE,                    # Add confidence interval
           conf.int.style = "step",            # CI style, use "step" or "ribbon"
           censor = FALSE,                     # Remove censor points
           tables.theme = theme_cleantable(),  # Clean risk table
           palette = "jco",ylim=c(0.9,1))
resd<-resid(all_RandomEffects2,"mart")
plot(resd)
hist(resd)
#residual plots
dev.off()
plot(resd~as.factor(data_all2y$age_category))
plot(resd~as.factor(data_all2y$sex))
plot(resd~as.factor(data_all2y$rural_urban))
plot(resd~as.factor(data_all2y$specialty4))
plot(resd~as.factor(data_all2y$insurance))
plot(resd~as.factor(data_all2y$referring_type))
plot(resd~as.factor(data_all2y$healthdept))
plot(resd~as.factor(data_all2y$accepting_type))
#interaction plots
par(mfrow=c(2,3))
interaction.plot(as.factor(data_all2y$age_category), as.factor(data_all2y$sex), resd)
interaction.plot(as.factor(data_all2y$age_category), as.factor(data_all2y$rural_urban), resd)
interaction.plot(as.factor(data_all2y$age_category), as.factor(data_all2y$specialty4), resd)
interaction.plot(as.factor(data_all2y$age_category), as.factor(data_all2y$insurance), resd)
interaction.plot(as.factor(data_all2y$age_category), as.factor(data_all2y$referring_type), resd)
interaction.plot(as.factor(data_all2y$age_category), as.factor(data_all2y$healthdept), resd)
#Update model with the interaction
all_fixedEffect_Interactions<-coxph(Surv(time_to_event2y, death2y)~
                                      age_category+
                                      sex+
                                      rural_urban+
                                      specialty4+
                                      insurance+
                                      referring_type+
                                      healthdept+
                                      accepting_type+
                                      age_category:sex,
                                    #age_category:healthdept,
                                    data=data_all2y)
#Checking model results
summary(all_RandomEffects2)
fit_interactions<-survfit(all_fixedEffect_Interactions,data=data_all2y)
obs<-survfit(Surv(time_to_event2y, death2y)~1, data=data_all2y)
KmPh <- list(Model = fit, KM = obs, Interactions=fit_interactions)
ggsurvplot(KmPh, data = data_all2y, combine = TRUE, # Combine curves
           risk.table = TRUE,                  # Add risk table
           conf.int = TRUE,                    # Add confidence interval
           conf.int.style = "step",            # CI style, use "step" or "ribbon"
           censor = FALSE,                     # Remove censor points
           tables.theme = theme_cleantable(),  # Clean risk table
           palette = "jco",ylim=c(0.9,1))
```

```
summary(as.factor(data_all2y$death2y))
sum(data_all2y$death2y)/length(data_all2y$death2y)*100
#####################################################################################################
#####################################################################################
```

```
#4. STATISTICAL ANALYSIS: MEDICAL CENTER-LEVEL CORRELATION ANALYSIS####
##CORRELATION ALL MEDICAL CENTERS####
all_RandomEffects2_Center<-coxph(Surv(time_to_event2y, death2y)~
                                   age_category+
                                   sex+
                                   rural_urban+
                                   specialty4+
                                   insurance+
                                   referring_type+
                                   accepting,
                                 data=data_all2y)
#median waiting
waiting<-c(0,
           120,
           139.5,
           77,
           145,
           100,
           36,
           86,
           104,
           8,
           0,
           92.5,
           92,
           98.5,
           101,
           97,
           48,
           56,
           1,
           7,
           139,
           43,
           104,
           174.5,
           143,
           59,
           91,
           89,
           0,
           0,
           0,
           51,
           60,
```

```
           70,
           71,
           71,
           71,
           71,
           71,
           122,
           36)
RR<-c(0.000003084,
      0.000003134,
      0.00000317,
      0.000003211,
      0.000003498,
      0.000003525,
      0.000003541,
      0.000003552,
      0.000003642,
      0.000004914,
      0.000005047,
      0.000005419,
      0.000005788,
      0.00000635,
      0.000006628,
      0.1808,
      0.2187,
      0.271,
      0.3797,
      0.425,
      0.4325,
      0.4612,
      0.4637,
      0.5638,
      0.6166,
      0.6514,
      0.6578,
      0.7457,
      0.7579,
      0.7788,
      0.8503,
      0.9584,
      0.9629,
      1.047,
      1.426,
      1.656,
      1.656,
      1.656,
      1.656,
      1.962,
      2.008)
cor.test(waiting, RR, method = "kendall", alternative = "two.sided")
#average waiting
waiting<-c(125.186667,
           150.470588,
           195.630435,
           131.619835,
           254.258065,
           152.516129,
           50.492918,
           158.858025,
           194.753894,
           24.819905,
           91.61194,
           112.005155,
           97.548387,
           113.948276,
           96.165062,
           91.72833,
           85.210247,
           81.541219,
```

```
          95.55538,
          14.457803,
          189.232812,
          89.450768,
          125.263687,
          227.617849,
          215.618705,
          70.026905,
          117.051944,
          150.290498,
          1.178868,
          140.631579,
          172.370968,
          122.705908,
          87.800578,
          125.612245,
          174.643837,
          174.643837,
          174.643837,
          174.643837,
          174.643837,
          148.955882,
          167.762992)
cor.test(waiting, RR, method = "kendall", alternative = "two.sided")
#SD of waiting
waiting<-c(187.7244,
          150.3531,
          202.88161,
          141.50396,
          225.29077,
          126.67935,
          45.49408,
          179.6641,
          270.54994,
          42.72976,
          193.46811,
          123.0942,
          36.42603,
          33.71054,
          55.27824,
          43.06713,
          103.88298,
          81.7793,
          202.4768,
          19.10705,
          169.99146,
          121.14803,
          100.82476,
          205.20558,
          243.34717,
          79.85872,
          104.0488,
          171.04455,
          22.96973,
          226.86953,
          252.37172,
          170.69587,
          140.98386,
          170.56448,
          258.49471,
          258.49471,
          258.49471,
          258.49471,
          258.49471,
          104.54485,
          330.03614)
cor.test(waiting, RR, method = "kendall", alternative = "two.sided")

##CORRELATION STRATIFIED BY LEVEL OF CARE####
```

```
##TERTIARY AND SECONDARY CARE####
#median
waiting<-c(36,
           71,
           71,
           71,
           71,
           89,
           51)
RR<-c(2.008,
      1.426,
      1.656,
      1.656,
      1.656,
      0.7457,
      0.9584)
cor.test(waiting, RR, method = "kendall", alternative = "two.sided", exact=FALSE)
#average waiting
waiting<-c(167.762992,
           174.643837,
           174.643837,
           174.643837,
           174.643837,
           150.290498,
           122.705908)
cor.test(waiting, RR, method = "kendall", alternative = "two.sided", exact=FALSE)
#SD of waiting
waiting<-c(330.03614,
           258.49471,
           258.49471,
           258.49471,
           258.49471,
           171.04455,
           170.69587)
cor.test(waiting, RR, method = "kendall", alternative = "two.sided", exact=FALSE)
##PRIMARY CARE####
#median waiting
waiting<-c(104,
           71,
           0,
           143,
           0,
           145,
           174.5,
           139.5,
           1,
           0,
           0,
           86,
           70,
           139,
           120,
           77,
           60,
           100,
           92.5,
           43,
           122,
           91,
           48,
           104,
           56,
           59,
           101,
           36,
           97,
           8,
           92,
           98.5,
```

```
           0,
           7)
RR<-c(0.000003642,
      1.656,
      0.8503,
      0.6166,
      0.7788,
      0.000003498,
      0.5638,
      0.00000317,
      0.3797,
      0.000005047,
      0.000003084,
      0.000003552,
      1.047,
      0.4325,
      0.000003134,
      0.000003211,
      0.9629,
      0.000003525,
      0.000005419,
      0.4612,
      1.962,
      0.6578,
      0.2187,
      0.4637,
      0.271,
      0.6514,
      0.000006628,
      0.000003541,
      0.1808,
      0.000004914,
      0.000005788,
      0.00000635,
      0.7579,
      0.425)
cor.test(waiting, RR, method = "kendall", alternative = "two.sided", exact=FALSE)
#average waiting
waiting<-c(194.753894,
           174.643837,
           172.370968,
           215.618705,
           140.631579,
           254.258065,
           227.617849,
           195.630435,
           95.55538,
           91.61194,
           125.186667,
           158.858025,
           125.612245,
           189.232812,
           150.470588,
           131.619835,
           87.800578,
           152.516129,
           112.005155,
           89.450768,
           148.955882,
           117.051944,
           85.210247,
           125.263687,
           81.541219,
           70.026905,
           96.165062,
           50.492918,
           91.72833,
           24.819905,
           97.548387,
```

```
           113.948276,
           1.178868,
           14.457803)
cor.test(waiting, RR, method = "kendall", alternative = "two.sided", exact=FALSE)
#SD of waiting
waiting<-c(270.54994,
           258.49471,
           252.37172,
           243.34717,
           226.86953,
           225.29077,
           205.20558,
           202.88161,
           202.4768,
           193.46811,
           187.7244,
           179.6641,
           170.56448,
           169.99146,
           150.3531,
           141.50396,
           140.98386,
           126.67935,
           123.0942,
           121.14803,
           104.54485,
           104.0488,
           103.88298,
           100.82476,
           81.7793,
           79.85872,
           55.27824,
           45.49408,
           43.06713,
           42.72976,
           36.42603,
           33.71054,
           22.96973,
           19.10705)
cor.test(waiting, RR, method = "kendall", alternative = "two.sided", exact=FALSE)
##CORRELATION FOR RISKY MEDICAL CENTERS:WAITING AND HAZARD RATIO >50th PERCENTILE####
myfun1 <- function(x){c(m=mean(x), s=sd(x), N=length(x))}
Mortality<-as.data.frame(summaryBy(death2y ~ specialty4,data=data_all2y, FUN=myfun1))
Waiting<-as.data.frame(summaryBy(waitingtime_numeric ~ specialty4,data=data_all2y, FUN=myfun1))
MortalityAndWaiting<-merge(Mortality,Waiting,by="specialty4")
Mortality<-NULL
Waiting<-NULL
HighMortality_HighWaitingVariability<-
as.data.frame(MortalityAndWaiting[which(MortalityAndWaiting$death2y.m>summary(MortalityAndWaiting$death2y.
m)[3] &
MortalityAndWaiting$waitingtime_numeric.s>summary(MortalityAndWaiting$waitingtime_numeric.s)[3]),])
#Overall - mixed-effects Cox model (these are the ones used in main manuscript Fig 3 [sensitivity analysis
including high mortality and high waiting time variability])
all_RandomEffects2_Center_RiskyAndVariable<-coxph(Surv(time_to_event2y, death2y)~
                                                    age_category+
                                                    sex+
                                                    rural_urban+
                                                    specialty4+
                                                    insurance+
                                                    referring_type+
                                                    accepting,
                                                  data=data_all2y[which(data_all2y$specialty4 %in%
HighMortality_HighWaitingVariability$specialty4),])
summary(all_RandomEffects2_Center_RiskyAndVariable)
#median waiting
waiting<-c(36,
           104,
           71,
           71,
```

```
           71,
           71,
           0,
           143,
           0,
           1,
           0,
           0,
           86,
           89,
           51,
           70,
           60,
           59,
           97)
RR<-c(1.945,
      0.000007315,
      1.71,
      1.71,
      1.71,
      1.71,
      0.00001038,
      1.157,
      0.000004933,
      0.3098,
      0.000004252,
      0.000007654,
      0.00001181,
      0.6848,
      0.7975,
      0.00001039,
      0.000004422,
      0.000006312,
      0.00002732)
cor.test(waiting, RR, method = "kendall", alternative = "two.sided", exact=FALSE)
#average waiting
waiting<-c(167.762992,
           194.753894,
           174.643837,
           174.643837,
           174.643837,
           174.643837,
           172.370968,
           215.618705,
           140.631579,
           95.55538,
           91.61194,
           125.186667,
           158.858025,
           150.290498,
           122.705908,
           125.612245,
           87.800578,
           70.026905,
           91.72833)
cor.test(waiting, RR, method = "kendall", alternative = "two.sided", exact=FALSE)
#SD of waiting
waiting<-c(330.03614,
           270.54994,
           258.49471,
           258.49471,
           258.49471,
           258.49471,
           252.37172,
           243.34717,
           226.86953,
           202.4768,
           193.46811,
           187.7244,
```

```
            179.6641,
            171.04455,
            170.69587,
            170.56448,
            140.98386,
            79.85872,
            43.06713)
cor.test(waiting, RR, method = "kendall", alternative = "two.sided", exact=FALSE)


##COMPARING WAIT TIMES BY SPECIALTY AND MORTALITY STATUS
wilcox.test(data_all2y[which(data_all2y$specialty4=="Internal
Medicine"),]$waitingtime_numeric~data_all2y[which(data_all2y$specialty4=="Internal Medicine"),]$death2y)
wilcox.test(data_all2y[which(data_all2y$specialty4=="Adult
Surgery"),]$waitingtime_numeric~data_all2y[which(data_all2y$specialty4=="Adult Surgery"),]$death2y)
wilcox.test(data_all2y[which(data_all2y$specialty4=="Anesthesiology"),]$waitingtime_numeric~data_all2y[whi
ch(data_all2y$specialty4=="Anesthesiology"),]$death2y)
wilcox.test(data_all2y[which(data_all2y$specialty4=="Breast
Surgery"),]$waitingtime_numeric~data_all2y[which(data_all2y$specialty4=="Breast Surgery"),]$death2y)
wilcox.test(data_all2y[which(data_all2y$specialty4=="Broncopulmonary"),]$waitingtime_numeric~data_all2y[wh
ich(data_all2y$specialty4=="Broncopulmonary"),]$death2y)
wilcox.test(data_all2y[which(data_all2y$specialty4=="Cardiology"),]$waitingtime_numeric~data_all2y[which(d
ata_all2y$specialty4=="Cardiology"),]$death2y)
wilcox.test(data_all2y[which(data_all2y$specialty4=="Cardiovascular
Surgery"),]$waitingtime_numeric~data_all2y[which(data_all2y$specialty4=="Cardiovascular
Surgery"),]$death2y)
wilcox.test(data_all2y[which(data_all2y$specialty4=="Dentistry"),]$waitingtime_numeric~data_all2y[which(da
ta_all2y$specialty4=="Dentistry"),]$death2y)
wilcox.test(data_all2y[which(data_all2y$specialty4=="Dermatology"),]$waitingtime_numeric~data_all2y[which(
data_all2y$specialty4=="Dermatology"),]$death2y)
wilcox.test(data_all2y[which(data_all2y$specialty4=="Endocrinology"),]$waitingtime_numeric~data_all2y[whic
h(data_all2y$specialty4=="Endocrinology"),]$death2y)
wilcox.test(data_all2y[which(data_all2y$specialty4=="Family
Medicine"),]$waitingtime_numeric~data_all2y[which(data_all2y$specialty4=="Family Medicine"),]$death2y)
wilcox.test(data_all2y[which(data_all2y$specialty4=="Gastroenterology"),]$waitingtime_numeric~data_all2y[w
hich(data_all2y$specialty4=="Gastroenterology"),]$death2y)
wilcox.test(data_all2y[which(data_all2y$specialty4=="Genetics"),]$waitingtime_numeric~data_all2y[which(dat
a_all2y$specialty4=="Genetics"),]$death2y)
wilcox.test(data_all2y[which(data_all2y$specialty4=="Hematology"),]$waitingtime_numeric~data_all2y[which(d
ata_all2y$specialty4=="Hematology"),]$death2y)
wilcox.test(data_all2y[which(data_all2y$specialty4=="Infectious
Disease"),]$waitingtime_numeric~data_all2y[which(data_all2y$specialty4=="Infectious Disease"),]$death2y)
wilcox.test(data_all2y[which(data_all2y$specialty4=="Maxillofacial
Surgery"),]$waitingtime_numeric~data_all2y[which(data_all2y$specialty4=="Maxillofacial
Surgery"),]$death2y)
wilcox.test(data_all2y[which(data_all2y$specialty4=="Neonatology"),]$waitingtime_numeric~data_all2y[which(
data_all2y$specialty4=="Neonatology"),]$death2y)
wilcox.test(data_all2y[which(data_all2y$specialty4=="Nephrology"),]$waitingtime_numeric~data_all2y[which(d
ata_all2y$specialty4=="Nephrology"),]$death2y)
wilcox.test(data_all2y[which(data_all2y$specialty4=="Neurology"),]$waitingtime_numeric~data_all2y[which(da
ta_all2y$specialty4=="Neurology"),]$death2y)
wilcox.test(data_all2y[which(data_all2y$specialty4=="Neurosurgery"),]$waitingtime_numeric~data_all2y[which
(data_all2y$specialty4=="Neurosurgery"),]$death2y)
wilcox.test(data_all2y[which(data_all2y$specialty4=="Nutrition"),]$waitingtime_numeric~data_all2y[which(da
ta_all2y$specialty4=="Nutrition"),]$death2y)
wilcox.test(data_all2y[which(data_all2y$specialty4=="Obstetrics and
Gynecology"),]$waitingtime_numeric~data_all2y[which(data_all2y$specialty4=="Obstetrics and
Gynecology"),]$death2y)
wilcox.test(data_all2y[which(data_all2y$specialty4=="Oncology"),]$waitingtime_numeric~data_all2y[which(dat
a_all2y$specialty4=="Oncology"),]$death2y)
wilcox.test(data_all2y[which(data_all2y$specialty4=="Ophtalmology"),]$waitingtime_numeric~data_all2y[which
(data_all2y$specialty4=="Ophtalmology"),]$death2y)
wilcox.test(data_all2y[which(data_all2y$specialty4=="Other"),]$waitingtime_numeric~data_all2y[which(data_a
ll2y$specialty4=="Other"),]$death2y)
wilcox.test(data_all2y[which(data_all2y$specialty4=="Otorhinolaryngology"),]$waitingtime_numeric~data_all2
y[which(data_all2y$specialty4=="Otorhinolaryngology"),]$death2y)
wilcox.test(data_all2y[which(data_all2y$specialty4=="Pediatrics"),]$waitingtime_numeric~data_all2y[which(d
ata_all2y$specialty4=="Pediatrics"),]$death2y)
```

```
wilcox.test(data_all2y[which(data_all2y$specialty4=="Physical Medicine and
Rehabilitation"),]$waitingtime_numeric~data_all2y[which(data_all2y$specialty4=="Physical Medicine and
Rehabilitation"),]$death2y)
wilcox.test(data_all2y[which(data_all2y$specialty4=="Plastic
Surgery"),]$waitingtime_numeric~data_all2y[which(data_all2y$specialty4=="Plastic Surgery"),]$death2y)
wilcox.test(data_all2y[which(data_all2y$specialty4=="Proctological
Surgery"),]$waitingtime_numeric~data_all2y[which(data_all2y$specialty4=="Proctological
Surgery"),]$death2y)
wilcox.test(data_all2y[which(data_all2y$specialty4=="Psychiatry"),]$waitingtime_numeric~data_all2y[which(d
ata_all2y$specialty4=="Psychiatry"),]$death2y)
wilcox.test(data_all2y[which(data_all2y$specialty4=="Rheumatology"),]$waitingtime_numeric~data_all2y[which
(data_all2y$specialty4=="Rheumatology"),]$death2y)
wilcox.test(data_all2y[which(data_all2y$specialty4=="Sexual Transmitted
Disease"),]$waitingtime_numeric~data_all2y[which(data_all2y$specialty4=="Sexual Transmitted
Disease"),]$death2y)
wilcox.test(data_all2y[which(data_all2y$specialty4=="Traumatology"),]$waitingtime_numeric~data_all2y[which
(data_all2y$specialty4=="Traumatology"),]$death2y)
wilcox.test(data_all2y[which(data_all2y$specialty4=="Urology"),]$waitingtime_numeric~data_all2y[which(data
_all2y$specialty4=="Urology"),]$death2y)
```
